# Chiral induction in covalent organic frameworks

**DOI:** 10.1038/s41467-018-03689-9

**Published:** 2018-04-03

**Authors:** Xing Han, Jie Zhang, Jinjing Huang, Xiaowei Wu, Daqiang Yuan, Yan Liu, Yong Cui

**Affiliations:** 1 School of Chemistry and Chemical Engineering and State Key Laboratory of Metal Matrix Composites, Shanghai Jiao Tong University, Shanghai, 200240 China; 2 State Key Laboratory of Structural Chemistry, Fujian Institute of Research on the Structure of Matter, Chinese Academy of Sciences, Fuzhou, 350002 China; 3 Collaborative Innovation Center of Chemical Science and Engineering, Tianjin, 300072 China

## Abstract

Synthetic control over chirality and function is the crowning achievement for metal-organic frameworks, but the same level of control has not been achieved for covalent organic frameworks (COFs). Here we demonstrate chiral COFs (CCOFs) can be crystallized from achiral organic precursors by chiral catalytic induction. A total of nine two-dimensional CCOFs are solvothermally prepared by imine condensations of the *C*_3_-symmetric 1,3,5-triformylphloroglucinol (Tp) with diamine or triamine linkers in the presence of catalytic amount of (*R*)- or (*S*)-1-phenylethylamine. Homochirality of these CCOFs results from chiral catalyst-induced immobilization of threefold-symmetric tris(*N*-salicylideneamine) cores with a propeller-like conformation of one single handedness during crystallization. The CCOF-TpTab showed high enantioselectivity toward chiral carbohydrates in fluorescence quenching and, after postsynthetic modification of enaminone groups located in chiral channels with Cu(II) ions, it can also be utilized as a heterogeneous catalyst for the asymmetric Henry reaction of nitroalkane with aldehydes.

## Abstract (web summary)

Controlling chirality and function in metal organic frameworks has been an achievement, but very difficult to carry out in covalent organic frameworks. Here the authors show chiral covalent organic frameworks that are crystallized from achiral precursors by chiral catalytic induction.

## Introduction

Chirality is an essential feature of life and plays a vital role in various chemical and biological processes. Control of chirality in solids is of significant importance as there are many potential applications such as asymmetric catalysis^1,2^, chiral separations^3,4^, sensing^5^, magnetism^6^, and nonlinear optics^7,8^. Two typical strategies have been exploited to crystallize homochiral materials: the first utilizes chiral building blocks^9–11^ to make chiral frameworks while the second requires an enantiopure agent to induce the chiral product without entering the structure^12^. Most homochiral materials prepared thus far consist of optical active building blocks that translate their inherent chirality to the resulting crystals by a chirality conservation process. The globally homochiral crystallization from achiral precursors is an uncommon and yet highly desirable process. There are a few of excellent reports demonstrating the phenomenon of chiral induction to fabricate homochiral metal-organic materials^13–16^ and inorganic crystals such as NaClO_3_^17^, but the method remains elusive due to the unpredictability of a suitable chiral additive for a given set of achiral building blocks.

Covalent organic frameworks (COFs) are an emerging class of crystalline polymers that allow precise integration of organic building blocks into two-dimensional (2D)^18–25^ or three-dimensional (3D)^26–29^ networks with tunability of composition, structure, and function, similar to those found in metal-organic frameworks (MOFs)^30,31^. Synthetic control over chirality and function is the crowning achievement for MOFs; however, the same level of control has not been achieved for COFs. COF crystallization is inherently less controllable than MOFs crystallization, and there are only several CCOFs with chiral pyrrolidine^32–34^ and tartaric acid derivatives as linking^35^ or pendant groups^36^ having been prepared. No CCOFs has been reported to be crystallized by chiral induction from achiral organic monomers.

**Fig. 1:**
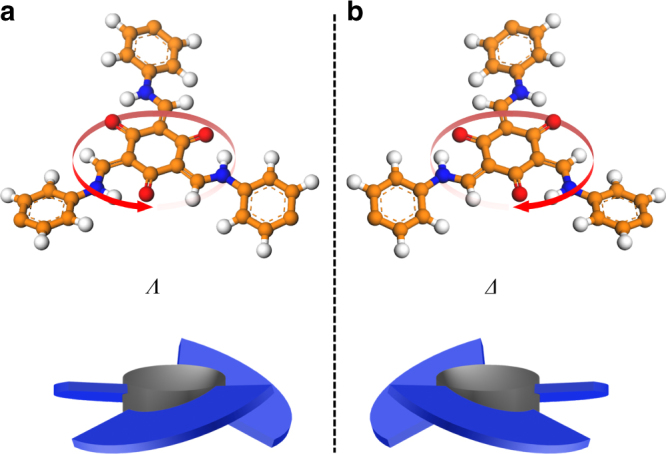
The modeling of propeller-shaped TASN-based conjugated structures. **a** and **b** are (*Λ*)*-* and (*Δ*)-isomers of TASN-based conjugated structures in COFs, respectively

Self-organization of an achiral or a dynamically racemic molecule into well-defined helical structures with controllable handedness is one of the exciting topics in supramolecular chirality^37^. Unidirectional tilting of three bulky groups such as aromatic rings around a *C*_3_-symmetric core can provide equal probability either a (*Λ*)- or (*Δ*)-isomer of a three-bladed propeller geometry (Fig. 1). Chiral induction, however, can potentially bias this dynamic process to increase the population of one helix sense over the other. The *C*_3_-symmetric tris(*N*-salicylideneamine) [TASN, C_6_O_3_(CHNH)_3_] derivatives have one central ring surrounded by three hydrogen-bonded six-membered rings that trace the shape of trisubstituted triphenylenes^38^. Conformational switching of TASN-derived molecules could be driven by organizing and disrupting mutually reinforcing hydrogen bonding networks, thereby providing good opportunities to prepare homochiral materials by chiral induction^39^ (Fig. 1). Recently, 1,3,5-triformylphloroglucinol (Tp) has been used to construct robust 2D COFs where the concept of proton tautomerism that has given exceptional stability to the framework.

**Fig. 2:**
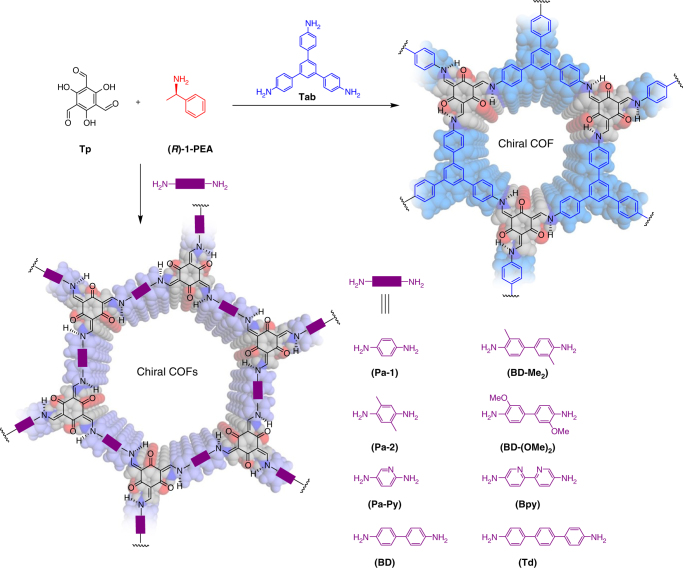
Schematic representation of the synthesis of CCOFs. These CCOFs are formed from achiral precursors by chiral catalytic induction

Here we demonstrated homochiral crystallization of a total of nine CCOFs with controlled handedness by imine condensations of Tp with achiral diamine or triamine linkers in the presence of chiral catalyst (Fig. 2). We showed that the CCOF-TpTab could be utilized as a chiral solid fluorescent receptor for a wide range of disaccharides with high binding affinity and enantioselectivity, and after metallation, as a heterogeneous catalyst for asymmetric Henry reactions.

## Results

### Synthesis and characterization of CCOFs

We prepared Tp-based CCOFs under conditions mimicking those used for the synthesis of the achiral analogs^40–44^. Thus, the series of (*Λ*)- and (*Δ*)-CCOFs were synthesized from a 2:3:2 mixture of Tp, diamine, and (*S*)- or (*R*)-1-PEA or 1:1:1 mixture of Tp, triamine, and (*S*)- or (*R*)-1-PEA in mesitylene, dioxane, and 6 M aqueous acetic acid at 120 °C for 3 days, affording crystalline solids in 50–70% yields (Supplementary Fig. 5). Both the Fourier transform infrared (FT-IR) spectra (Supplementary Fig. 6) and the solid-state ^13^C cross-polarization/magic angle spinning nuclear magnetic resonance (CP-MAS NMR) spectra (Supplementary Fig. 9) of as-prepared CCOFs are very similar to those of the achiral Tp-based COFs. In particular, the absence of the characteristic C−H bending band of the methyl group of 1-PEA at 1370 cm^−1^ suggested that this template was not incorporated in the product (Supplementary Fig. 7), as also revealed by ^13^C CP-MAS NMR, in which carbon signals due to 1-PEA were not detected (Supplementary Fig. 9). Notably, the model compound tris(*N*-salicylideneaniline) was synthesized by the reaction of Tp with aniline under similar conditions to those COFs.

**Fig. 3:**
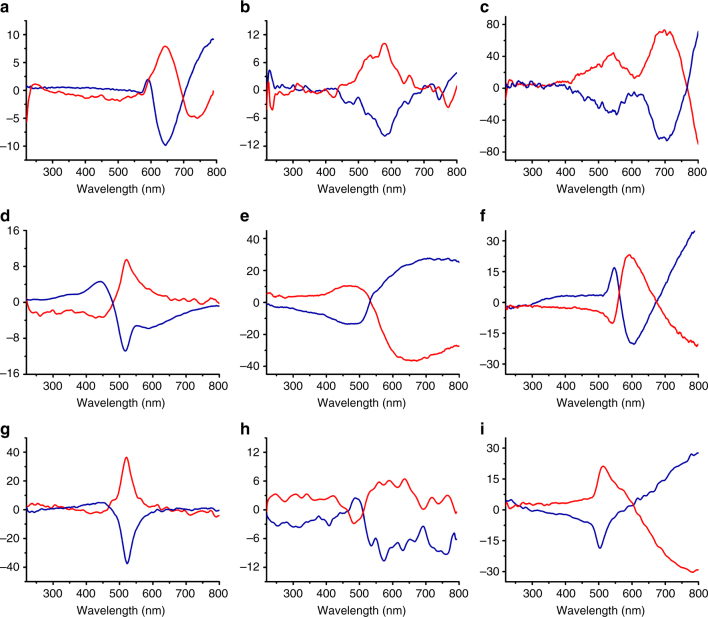
The solid-state CD spectra of CCOFs. **a** TpPa-1, **b** TpPa-2, **c** TpPa-Py, **d** TpBD, **e** TpBD-Me_2_, **f** TpBD-(OMe)_2_, **g** TpBpy, **h** TpTd, and **i** TpTab, respectively. CCOFs induced by (*R*)-1-PEA (red line) and by (*S*)-1-PEA (blue line)

All CCOFs prepared in the presence of (*S*)- or (*R*)-1-PEA were optically active and exhibited Cotton effects in the wavelength range 350−800 nm in the solid-state circular dichroism (CD) spectra (Fig. 3). The CD spectra, which were consistent with the corresponding UV–vis spectra (Supplementary Fig. 10), were mirror imaged of each other, indicative of their enantiomeric nature. By contrast, the COFs prepared in the absence of (*S*)- or (*R*)-1-PEA were optically inactive, as evidenced by the CD spectra. Meanwhile, the Schiff-base complexes prepared from a 1:3 mixture of Tp and (*S*)- or (*R*)-1-PEA (Supplementary Figs. 1–2) showed a negative and positive Cotton effect at 370 nm, respectively (Supplementary Fig. 12). The above results demonstrate the asymmetric catalytic effect of (*S*)- and (*R*)-1-PEA in the crystallization of CCOFs under the particular conditions used, which gives rise to the predominance of one (*Λ*)- or (*Δ*)-form over the other one in the bulk sample.

The crystalline structures of the nine CCOFs were evaluated by using powder X-ray diffraction (PXRD) analysis. PXRD indicated all of these CCOFs were highly crystalline and each had a similar pattern to the related achiral COF (Supplementary Fig. 17). To explore the source of the chirality, a perfectly defect-free crystal domain of *P*3 or *P*6 symmetry would be built up by TASN-based units that all have the same direction of rotation as the introduction of even a single TASN-based unit of the ‘wrong’ propeller enantiomer would cause considerable strain in the network, thereby synchronizing the molecular conformation of all the TASN-based units throughout a COF domain appears to be of great importance^45–47^. Consequently, we optimized the eclipsed tabular model to the chiral propeller stacking that adopted the *P*3 space group for (*Λ*)-TpPa-1, (*Λ*)-TpPa-2, (*Λ*)-TpPa-Py, (*Λ*)-TpBpy, (*Λ*)-TpTab, and the *P*6 space group for (*Λ*)-TpBD, (*Λ*)-TpBD-Me_2_, (*Λ*)-TpBD-(OMe)_2_, (*Λ*)-TpTd. The two space groups were the highest symmetry predicted from the DFT modeling. Because of the enol- to keto tautomerism in the TASN units, hydroxyl (−OH) and imine (C = N) groups disappeared and new C = C, C–N, and N–H bonds were generated. A rotation of the new C–N bond twisted the enamine group, leading to the propeller-like TASN core with a (*Λ*)-conformation. We therefore assumed that the enol to keto transformation was most likely a key step during chiral induction. This point was further supported by the fact that, under otherwise identical synthetic conditions, only achiral COFs can be crystallized from 1,3,5-triformylbenzene and Pa-1 or BD in the presence of (*R*)-1-PEA (Supplementary Fig. 13). The torsional motions about the C–N bonds around the *C*_3_-symmetric TASN core gave rise to the COFs with homochirality, which is more accurate than fully eclipsed tabular interaction for describing the 2D structures. We selected CCOFs TpPa-1, TpBD, TpBD-Me_2_, and TpTab as representative examples to describe the crystal structures.

**Fig. 4:**
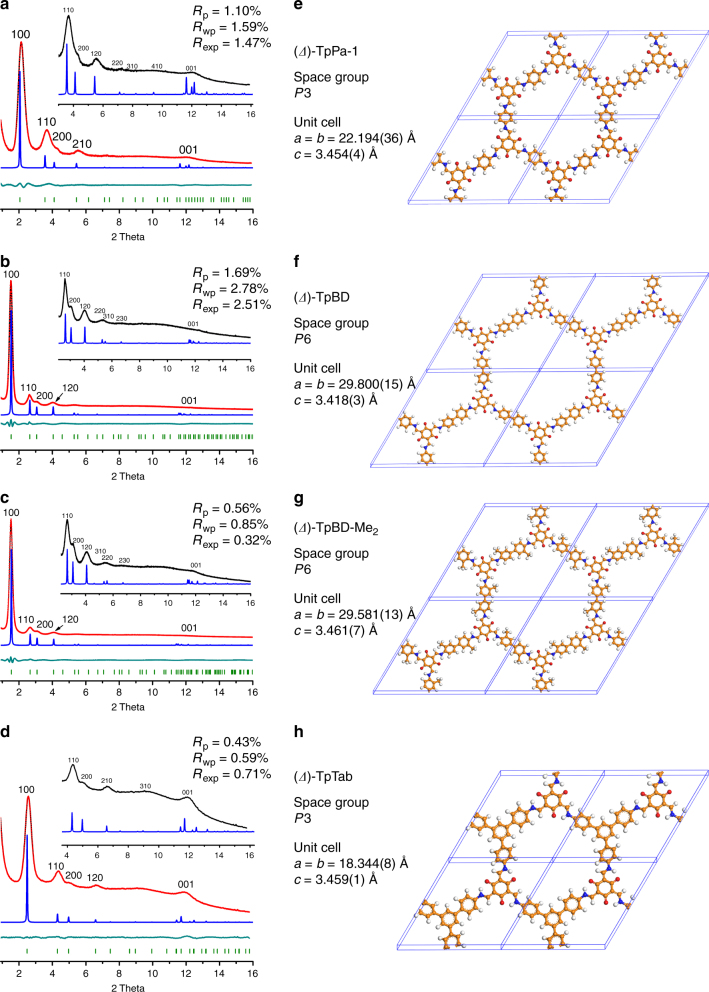
Synchrotron PXRD patterns and the corresponding Rietveld refined structures. **a–d** Experimental synchrotron PXRD data (black lines), simulated pattern (blue lines), and Rietveld refinements (red lines) of the CCOFs. Simultaneous Rietveld refinements of the high-resolution (red lines) provide good fits to the experimental data with only minimal differences (the dark cyan lines show the difference plots between the experimental PXRD patterns and those obtained by Rietveld refinements); Bragg positions are indicated by light green ticks. *λ* = 0.689 Å for the high-resolution data. TpPa-1 (**a**), TpBD (**b**), TpBD-Me_2_ (**c**), and TpTab (**d**). **e–h** The corresponding Rietveld refined structures of the TpPa-1 (**e**), TpBD (**f**), TpBD-Me_2_ (**g**), and TpTab (**h**)

High-resolution powder diffraction experiments were recorded on Beamline BL14B1 at Shanghai Synchrotron Radiation Facility (SSRF)^48,49^ (Fig. 4). Guided by the predicted structure, the crystal structures of CCOFs were built by Materials Studio 7.0. According to the synchrotron PXRD, Le Bail refinement was carried out using EXPO-2014^50^, and then the unit cell parameters were determined (Supplementary Fig. 18 and Supplementary Table 3). We performed density functional theory (DFT) geometry optimizations based on the obtained unit cell parameters using the CASTEP module. The Rietveld method refines the atom positions of these models to find the best match between the theoretical pattern and the experimental data. Refinements were undertaken in the *P*6 space group for TpBD and TpBD-Me_2_ or the *P*3 for TpPa-1 and TpTab, as they were the highest symmetries predicted from the DFT modelings. These structures were constrained to the DFT-optimized values as the rigid bodies and the optimized models provide an excellent fit to the measured diffraction intensities, as shown in Fig. 4 and Supplementary Tables 4–7. For example, the predicted structure of TpBD was validated with Rietveld refinement against PXRD. It gave optimized unit cell parameters of *a* = *b* = 29.80 Å, *c* = 3.42 Å, *α* = *β* = 90°, *γ* = 120° with space group *P*6, which provided good agreement factors (*R*_p_ = 1.69%, *R*_wp_ = 2.78%, *R*_exp_ = 2.51%) (Fig. 4b). Refinement results for other CCOFs are given in Fig. 4. The lattice parameters *a* and *b* varied from 18.34 Å for TpTab to 29.80 Å for TpBD, while the *c*-axis parameters fell into a rather narrow range of 3.42–3.46 Å, indicative of a similar 2D layer stacking in all cases despite the differences in stiffness and electronic interactions between different building blocks.

The simulated PXRD were generated by the Reflex tools in Materials Studio software. It can be revealed that there was no apparent difference in the simulated PXRD pattern between fully eclipsed tabular stacking and chiral propeller torsion stacking (Supplementary Fig. 17). As a result, such torsions are difficult to observe in powder diffraction data, as they would generally affect only the relative intensity and width of certain diffraction peaks and peak positions at small d-spacings^45^.

A comparison of the final total energy between the propeller torsion and fully eclipsed tabular structures is given in Supplementary Table 2. The propeller structures were found to be energetically more favorable than the fully eclipsed tabular structures. According to our simulations, the aromatic rings around the TASN core assumed a propeller-like arrangement. The dihedral angles between the tris(*N*-salicylideneamine) and the enamine planes are 11.7°, 5.3°, 1.4°, and 15.1° for TpPa-1, TpBD, TpBD-Me_2_, and TpTab, respectively, and those between the TASN and the adjacent aromatic planes are 17.4°, 17.9°, 20.1°, and 21.4°. The chiral propeller-like configuration of the TASN nodes in combination with the linear or triangular linkers thus allows the formation of a corrugated sheet network lying in the *ab* plane with 1D open channels of 13–24 Å. Such grids stacked on top of each other in a AA model along the *c*-axis to give porous lamellar solids, with interlayer separations of 3.42–3.46 Å. Unlike the MOFs, these COFs are not single crystals, ruling out the possibility of studying their structures using single crystal XRD. Because of the limits of data quality and lack of precision indicators, only reasonable atomic coordinates can be estimated from the PXRD data like the previous works although the residuals are very low. The chiral feature of TASN core in discrete molecular structure has been verified by single crystal XRD^51^.

**Fig. 5:**
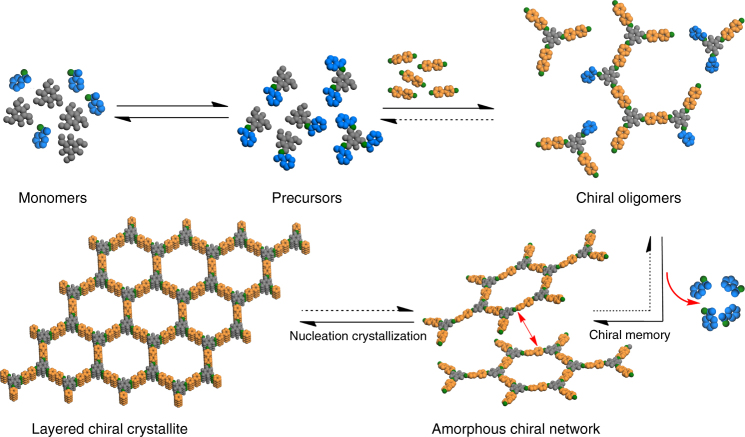
Proposed models of CCOF-TpBD growth. The enantiomer of 1-PEA was replaced by BD from the precursors, and the chiral memory left in the original position, thereby led to a torsion structure. After then the amorphous polymer crystallized and the CCOF formed

The CCOFs formation may like the homogeneous conditions of achiral COF growth^52,53^. The early stages of CCOFs growth presumably involve monomers condensing into soluble oligomers, similar to a step-growth polymerization. Subsequent nucleation affords CCOF crystallites of unknown structure and size, which might be as small as a single macrocyclic hexagon^52^ leading to a model for CCOF-TpBD formation, as shown in Fig. 5. Here, 1-PEA was utilized as a truncated equivalent of diamine or triamine, which reversibly formed imine with Tp that were nonproductive for the COF polymerization. The competitor 1-PEA may lengthen the induction period, inhibit precipitation, and add a certain chiral steric hindrance in their packing process. This point was supported the fact that no precipitation was observed when two equiv. of 1-PEA were used for CCOF synthesis. After the chiral 1-PEA was replaced by the diamine or triamine to grow frameworks, a chiral memory left in the original position, thereby leading to a torsion structure. Monitoring the crystallization process by ^1^H NMR further confirmed dynamic amine exchange occurred among 1-PEA and oligomers. After hydrolyzing the samples of TpPa-1 prepared by different crystallization time (2, 4, 12, 24, and 72 h), ^1^H NMR spectra showed that the ratios of 1,4-diaminobenzene to 1-PEA increased with time and no 1-PEA was detected in the final product (Supplementary Figs. 3–4). In addition, the optical rotation and solid-state CD spectra indicated that no thermally induced racemization of 1-PEA and the COF occurred under crystallization conditions (Supplementary Table 1 and Supplementary Figs. 12 and 14). Nevertheless, the nucleation and growth processes of the CCOFs are still poorly understood and further study is needed to elucidate the mechanism. The chiral nature of the product is controlled by the handedness of 1-PEA, as evidenced by that (*Λ*)-CCOF was induced by (*S*)-1-PEA and (*Δ*)-CCOF was induced by (*R*)-1-PEA^54^.

**Fig. 6:**
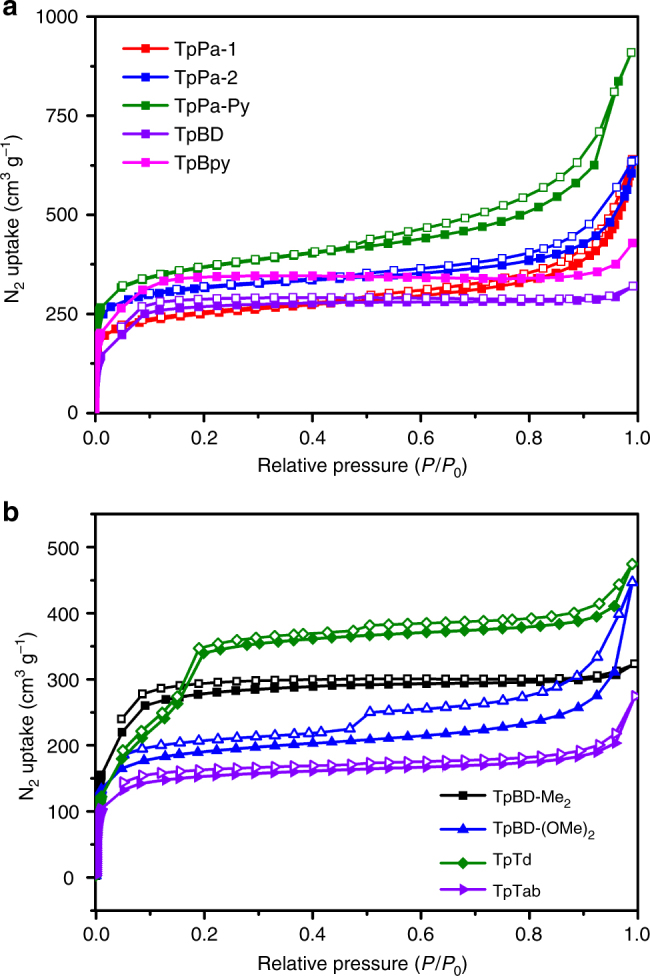
N_2_ adsorption and desorption isotherms of CCOFs. **a** TpPa-1, TpPa-2, TpPa-Py, TpBD, and TpBpy; **b** TpBD-Me_2_, TpBD-(OMe)_2_, TpTd, and TpTab

Scanning electron microscopy (SEM) images showed that these CCOFs exhibited spherical, flowerlike or irregular morphology, all of which can be considered as the fluffy aggregation of sheet-like structures (Supplementary Fig. 19). Transmission electron microscope (TEM) images show sheet-like morphologies for the polymer crystallites (Supplementary Fig. 20). These CCOFs can keep their crystalline state and chirality in the acid/base (Supplementary Figs. 15–16) and thermogravimetric analyses (TGA) indicated that all of these CCOFs were thermally stable up to about 350 °C (Supplementary Fig. 11). The permanent porosity of the nine CCOFs was examined by measuring N_2_ adsorption–desorption at 77 K on the activated samples (Fig. 6). All materials exhibited the type I or type IV reversible isotherms, which is the characteristic of porous materials. The Brunauer−Emmett− Teller (BET) surface areas for the CCOFs are 832.4 m^2^ g^−1^ (TpPa-1), 1077.4 m^2^ g^−1^ (TpPa-2), 1178.1 m^2^ g^−1^ (TpPa-Py), 849.3 m^2^ g^−1^ (TpBD), 878.1 m^2^ g^−1^ (TpBD-Me_2_), 602.8 m^2^ g^−1^ (TpBD-(OMe)_2_), 1073.4 m^2^ g^−1^ (TpBpy), 1217.8 m^2^ g^−1^(TpTd), and 482.6 m^2^ g^−1^ (TpTab), respectively. These BET values for CCOFs are comparable to or higher than those of the corresponding achiral COFs obtained without adding 1-PEA (Supplementary Table 8 and Supplementary Figs. 21–22). The pore size distributions for these CCOFs were found to be between 1.1 and 2.51 nm, calculated on the basis of non-local density functional theory (NLDFT), which were in agreement with the theoretical values predicted from their crystal structures (Supplementary Fig. 23).

### Enantioselective sensing of saccharides

The Tp series of CCOFs are highly fluorescent, especially TpTab showed a strong emission maximum at 540 nm excited by a *λ* = 387 nm with a lifetime of 0.79 ns (Supplementary Fig. 35). We examined the interaction of the two enantiomers of TpTab bearing enaminone groups with a variety of saccharides (Supplementary Table 9 and Supplementary Fig. 38). The sample of TpTab was crushed and suspended in phosphate-buffered saline (PBS) solution (pH = 7.35), which simulated the acid and alkali environment of human body fluids to prepare a stock suspension of TpTab (with the Tp building block at a concentration of 0.1 mM). When buffer suspensions of TpTab was treated with aliquots d-cellobiose, the emission at 540 nm was decreased by the disaccharide, but the rate of change with (*Λ*)-TpTab was faster than that with (*Δ*)-TpTab, implying enantioselectivity in the fluorescent recognition.

**Fig. 7:**
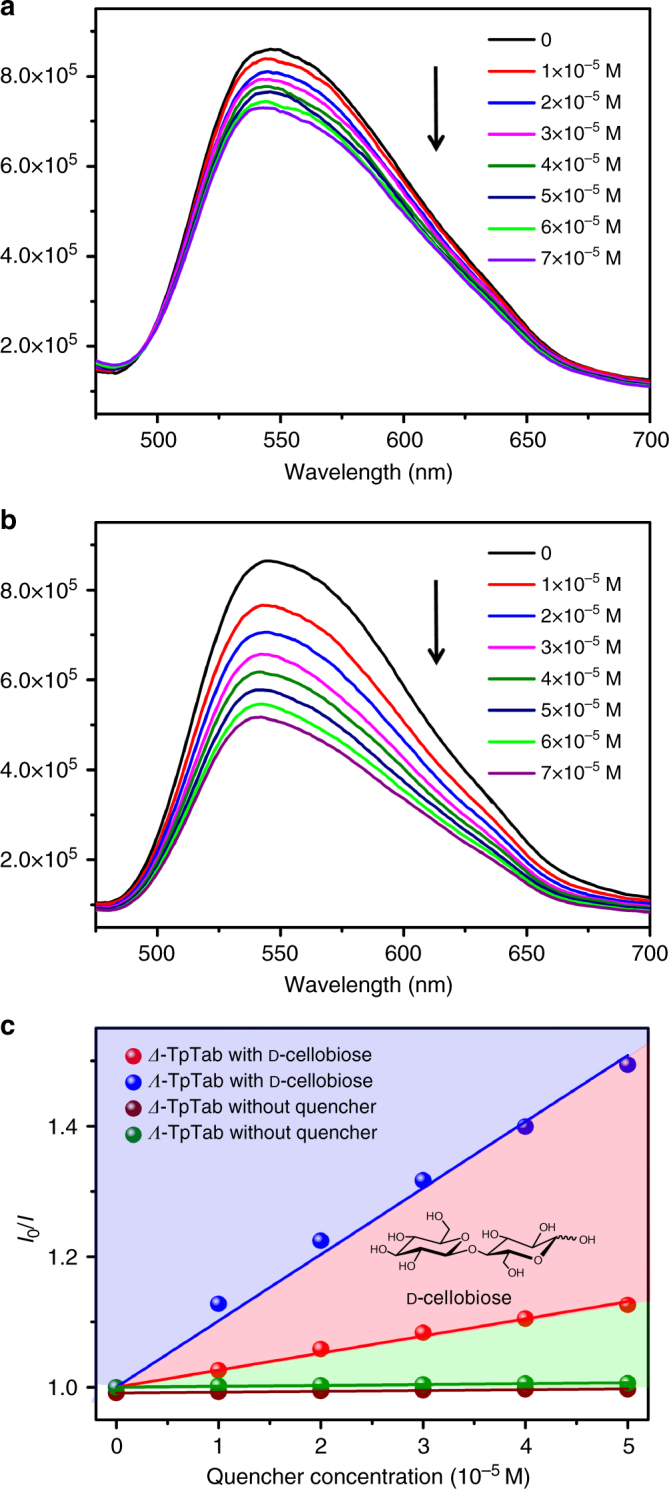
The fluorescence quench spectra of CCOFs TpTab. **a**, **b** Fluorescence emission spectra of (*Λ*)/(*Δ*)-TpTab with increasing concentration of the d-cellobiose quencher in solution: 0, 1, 2, 3, 4, 5, 6, and 7 × 10^−5^ M d-cellobiose from top to bottom. **c** SV plots of the fluorescence emissions of (*Λ*)/(*Δ*)-TpTab quenched by d-cellobiose. The control experiment with TpTab was performed under the conditions without addition of the quencher

The fluorescence signals of the TpTab suspensions with different amounts of disaccharide quenchers were measured. The quenching of fluorescence was highly efficient and followed Stern−Völmer (SV) behavior in the 0–7 × 10^−5^ mM concentration range (Fig. 7). The association constants (*K*_SV_) were calculated as 13,086 ± 1000 M^−1^ with (*Λ*)-TpTab and 3806 ± 200 M^−1^ with (*Δ*)-TpTab, giving a quenching ratio [QR = *K*_SV_(*Λ*-TpTab)/*K*_SV_(*Δ*-TpTab) of 3.44 ± 0.26:1. The fluorescence decrease may be due to static enhancement upon formation of a CCOF-saccharide adduct (Supplementary Fig. 8). Elemental analysis suggested the formation of the host–guest complex, which can be formulated as [TpTab·cellobiose] (Supplementary Table 10). Changes in the framework structure of the emitting species such as distortion of the conformation may be induced, along with excimer formation^55^. The supramolecular interactions of TpTab with saccharides form different diastereomeric complexes, giving rise to a distinct fluorescence decrement. The static nature of the complexation is suggested by consistent fluorescence lifetimes of TpTab before and after titration with d-cellobiose (0.79 vs 0.77 ns) (Supplementary Fig. 35). Moreover, the quantum yield decreased from 2.00% to 1.12% (Supplementary Figs. 36–37). After the titration, the peak position and shape of the UV-vis spectra of TpTab remained unchanged (Supplementary Fig. 39), indicative of the framework stability, as also supported by the PXRD pattern of TpTab after exposure to d-cellobiose, which matched well with the pattern of the pristine sample (Supplementary Fig. 40).

Interestingly, this TpTab CCOF is enantioselective towards a wide range of saccharides, including d-glucose, d-mannitol, d-sucrose, d-lactose, d-maltose, d-sorbitol, d-fructose, d-gentiobiose, d-lactobionic acid, d-glucuronic acid, and d-gluconic acid. In all cases, the rates of fluorescence decrement for (*Δ*)-TpTab caused by d-saccharide were slower than those for (*Λ)*-TpTab. The QR values were determined as 1.56 ± 0.2, 2.18 ± 0.4, 2.43 ± 0.16, 1.59 ± 0.25, 3.62 ± 0.16, 2.24 ± 0.19, 2.49 ± 0.21, 2.66 ± 0.1, 2.00 ± 0.30, 1.32 ± 0.41, and 2.03 ± 0.28, respectively (Supplementary Figs. 24–34). Notably, the QR values for the examined saccharides are of the same order of magnitude as those obtained for the well-known binol–bisboronic-acid-based fluorescence sensors for sugar acids and sugar alcohols^56,57^.

### Heterogeneous asymmetric catalysis

**Fig. 8:**
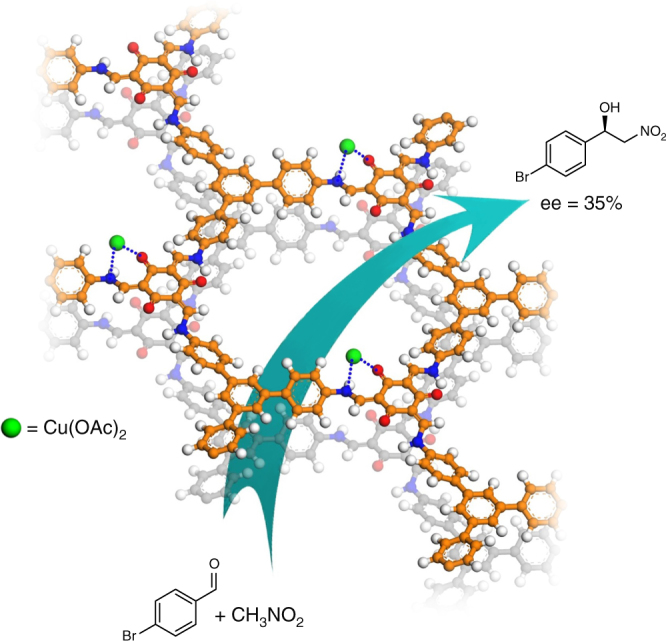
Henry reaction. The reaction  between nitroalkane with aldehyde was catalyzed by CCOF-TpTab-Cu

We have employed the CCOFs for heterogeneous asymmetric catalysis by taking advantage of the enaminone groups exposed in the chiral channels. The presences of the carbonyl group connected by carbon–carbon double bond and an *N*-substituted amine in enaminones allow being acted as bidentate ligands for metal ions. Cu(OAc)_2_ reacted with one enaminone in the presence of base to give a [R’C(O)CHC(NAr)R]Cu(OAc) compound which can be used as a Lewis acidic catalyst (Fig. 8). We resorted to XAFS to determine the Cu coordination environment in TpTab-Cu. The scattering path distances and degeneracies derived from these fits are consistent with tetrahedral coordination of the Cu centers with two O atoms of the acetate, one O and one N atoms of CCOF-TpTab (Supplementary Fig. 47). Treatment of TpTab with excess Cu(OAc)_2_ in the presence of *N*,*N*-diisopropylethylamine (DIEA, 0.4 equiv.) indeed led to a catalyst (designated as TpTab-Cu) for the reaction of nitroalkane with aldehydes (Henry reaction), which is one of the most important routes for C–C bond formation. In the X-ray photoelectron spectroscopy (XPS) spectra of TpTab-Cu, the Cu 2p_3/2_ line appeared at 933 eV, indicating the Cu species is in +2 oxidation state (Supplementary Fig. 46). ICP-OES results reveal that the Cu loading amount in CCOF-TpTab-Cu is 1.2 wt%. As shown in Fig. 8, the addition of nitromethane to 4-bromobenzaldehyde was catalyzed by the in situ prepared (*Δ*)-TpTab-Cu (0.5 equiv.) in the presence of DIEA (0.4 equiv.) in mesitylene at −10 °C for 48 h, affording the corresponding β-nitroalcohol with 10% conversion of aldehyde and 35% enantiomeric excess (ee). The chiral nature of the alcohol product is determined by the handedness of the solid catalyst, as shown by that the addition by (*Λ*)-TpTab-Cu gave the *S-*enantiomer over the *R*-enantiomer (31% ee) (Supplementary Figs. 41–44). When more DIEA, an additive to accelerate the Henry reaction, was used, the conversion of aldehyde could increase up to 90% in 48 h, but with very low or no enantioselctivity (Supplementary Table. 11) because elevated levels of DIEA may destroy the crystallinity of CCOF-TpTab. The reason for low ee% may be partly ascribed to the modest enantiomeric purity of the COF TpTab^12^. Besides, the chirality of these COFs originates from helix-induced characteristics of *C*_3_-symmetric tris(*N*-salicylideneamine) cores and the CCOFs do not have any typical chiral active sites, which may be also responsible for low enantioselectivity observed in catalysis^2,3^. In contrast, despite a good conversion (>85%), the mode compound tris(*N*-salicylideneaniline) after binding to copper ions showed no enantioselectivity in the same reaction under identical conditions. Control experiments showed that the Henry reaction could not occur in the absence of TpTab and/or DIEA.

Moreover, the reaction of nitromethane with a more sterically demanding substrate, coronenyl aldehyde, could not happen, even with four equivalents of DIEA. Such size selectivity implied that the catalysis mainly occurred in the channels. The TpTab catalyst was readily recovered by centrifugation and reused at least for three times without loss of enantioselectivity (~10% conversion and 35, 35, and 31% ee for 1–3 run, respectively). The PXRD pattern of the recycled catalyst demonstrated that the crystallinity and covalent bonding were maintained (Supplementary Fig. 45).

The catalytic activity and enantioselectivity of this reaction are lower than those observed for molecular catalysts of chiral copper complexes^58^. It is also worth noting that, among the reported chiral framework catalysts including MOFs and COFs, the most efficient examples all contain isolated privileged chiral catalysts or ligands such as BINOL-, metallosalen-, and organocatalyst-based derivatives^3,11,32–35^. Although modest, the enantioselectivity of the present process is noteworthy because the investigation into chiral porous solids consisting of only achiral building blocks for asymmetric catalysis, enantioselective sensing and separation has thus far remained relatively unexplored^3,12^. Further studies on enantioselective catalytic activities of chiral-induced COFs, including a search for reactions and substrates that may lead to higher enantiomeric excess values, are currently underway.

## Discussion

We have demonstrated homochiral crystallization of CCOFs from achiral precursors by chiral catalysis. By using (*R*)- or (*S*)-1-PEA as a catalyst, a total of nine 2D CCOFs with controlled handedness were solvothermally synthesized by imine condensations of Tp with diamine or triamine linkers. Chiral catalysis induced fixation of propeller-like TASN cores in homochiral conformations during crystallization. The CCOF-TpTab exhibited high enantioselectivity toward chiral carbohydrates in fluorescence quenching in the simulated conditions of human body fluids and, after postsynthetic modification of the enaminone groups with Cu(II) ions, the solid was a recyclable heterogeneous catalyst for the asymmetric Henry reaction of nitroalkane with aldehydes. Further studies on homochiral crystallization of more COFs from achiral precursors and understanding the enantioselective processes are underway. The present study may greatly expand the scope of materials design and crystal engineering of new functional porous materials.

## Methods

### Synthesis of the CCOFs

The synthetic procedures are similar for all CCOFs. Typically, a mixture of Tp (32 mg, 0.15 mmol) and (*S*)- or (*R*)-1-PEA (18 mg, 0.15 mmol) in 3 mL of mesitylene/dioxane (1:1 v/v) (DMA/o-DCB (2:1 v/v) for Bpy) in a small vial was sonicated for 5 min to give a clear solution and then the diamine (Pa-1, Pa-2, Pa-Py, BD, BD-Me_2_, BD-(OMe)_2_, Bpy, or Td, 0.225 mmol) or the triamine Tab (0.15 mmol) was added. After stirring for 5 min, 0.6 mL aqueous acetic acid (6 mol L^−^^1^) was added, and the mixture was sonicated to afford a homogeneous dispersion. The solution was transferred into a Pyrex tube and degassed by three freeze–pump–thaw cycles. The tube was sealed off and heated at 120 °C for 3 days. The precipitate was collected by centrifugation and washed with anhydrous THF (6 × 4 mL) and acetone (3 × 4 mL). The powders were dried at 80 °C under vacuum overnight to afford the CCOFs in 50–70% isolated yields.

### Fluorescence titration experiments

1.7 mg of (*Δ*)- or (*Λ*)-TpTab (0.01 mmol) was placed in a vial. 10 mL of PBS (pH = 7.35) was added and the crystals were mechanically crushed by vigorous stirring with a stir bar overnight. The resulting cloudy suspension was diluted with buffer solution to 100 mL and 10 mg of CTAB was added as a stabilizer. Titration experiments were carried out by adding aliquots (20 μL) of the carbohydrate quencher in PBS (1.0 × 10^-3^ mol L^−^^1^) to 2 mL of the CCOF stock solution (1 × 10^−4^ mol L^−1^) at intervals of 10 min. Fluorescence spectra were recorded after addition of the quencher. Each scan collected intensity data for a duration of 3 s, while stirring, to minimize light exposure and prevent photodegradation. The excitation wavelength was 387 nm and the slit width was set at 4 nm × 4 nm.

### Asymmetric Henry reaction

To a dry sample of CCOF-TpTab (8.5 mg, 0.05 mmol) in 3 mL of anhydrous ethanol was added Cu(OAc)_2_·H_2_O (4 mg, 0.02 mmol) and the mixture was stirred for 24 h at 50 °C. The resulting solid was washed with anhydrous DMF and ethanol, and then dried under vacuum at 60 °C for 12 h. The residue was degassed and 4-bromobenzaldehyde (18.5 mg, 0.1 mmol), nitromethane (12 mg, 0.2 mmol), DIEA (5 mg, 0.04 mmol), and mesitylene (1 mL) were added. The reaction mixture was stirred at −10 °C for 2 days and the supernatant was concentrated under vacuum. The concentrate was analyzed by ^1^H NMR to give the conversion and by high-performance liquid chromatography (HPLC) to give the ee value.

### Characterization

The IR (KBr pellet) spectra were recorded (400–4000 cm^−^^1^ region) on a Nicolet Magna 750 FT-IR spectrometer. The solid-state UV spectra were recorded by using pressed BaSO_4_ matrix in the range of 200–800 nm at room temperature with a UV/Vis/NIR Spectrometer Lambda 750S (Perkin Elmer, Inc., USA). The solid-state CD spectra were recorded on a J-800 spectropolarimeter (Jasco, Japan). The optical rotation was recorded by an automatic polarimeter (PMS/JASCO P-2000) in dioxane using a sodium lamp (wavelength 589 nm, 150 W) at 23 °C. PXRD were collected on a D8 Advance diffractometer using Cu Kα radiation. The high-resolution synchrotron XRD patterns were measured at BL14B1 of the SSRF. ICP-OES was performed on Optima 7300DV ICP-OES (Perkin Elmer Corporation, USA). XPS was performed on AXIS UltraDLD. The X-ray absorption fine structure (XAFS) spectrum at CCOF-TpTab-Cu (*E*_0_ = 8979 eV) edge was performed at BL14W1 beamline of SSRF operated at 3.5 GeV under “top-up” mode with a constant current of 260 mA. SEM images were performed on NOVA NanoSEM NPE218 and Sirion 200 instruments. The TEM images were obtained with Talos F200X. NMR experiments were carried out on a MERCURY plus 400 spectrometer operating at resonance frequencies of 400 MHz. Solid ^13^C NMR experiments were carried out on a Bruker Avance III 400 MHz. The porous properties of the COFs were investigated by nitrogen adsorption and desorption at 77.3 K using ASAP 2020 V4.00 (V4.00H) Serial #: 1674 from USA. The pore-size-distribution curves were obtained from the adsorption branches using NLDFT method. ES-MS were recorded on a Finnigan LCQ mass spectrometer using dichloromethane–methanol as mobile phase. Elemental analyses of C, H, and N were performed with an EA1110 CHNS-0 CE elemental analyzer. TGA were carried out in an N_2_ atmosphere with a heating rate of 10 °C min^−1^ on a STA449C integration thermal analyzer. Analytical HPLC was performed on a YL-9100 HPLC with UV detection. Analytical CHIRALCEL OD-H column (4.6 mm × 25 cm) from Daicel was used. See Supplementary Methods for the procedures and characterization data of compounds not listed in this part.

### Data availability

The X-ray crystallographic coordinates for the structures reported in this article have been deposited at the Cambridge Crystallographic Data Centre (CCDC), under deposition number CCDC 1826392-1826395. These data can be obtained free of charge from The Cambridge Crystallographic Data Centre via www.ccdc.cam.ac.uk/data_request/cif. The authors declare that all the data supporting the findings of this study are available within the article (and Supplementary Information Files), or available from the corresponding author on reasonable request.

## Electronic supplementary material

Supplementary Information(PDF 5755 kb)
