# Supplementary material for: Chiral induction in covalent organic frameworks
Source: Nat Commun. 2018 Apr 3;9:1294. doi: 10.1038/s41467-018-03689-9 (PMC5882852; doi:10.1038/s41467-018-03689-9)
Supplement: Supplementary file 1 — Supplementary Information(PDF 5755 kb) [file 41467_2018_3689_MOESM1_ESM.pdf]

Chiral Induction in Covalent Organic Frameworks

Han et al

Supplementary Methods

Synthesis of the (*R*)-Tp-1-PEA

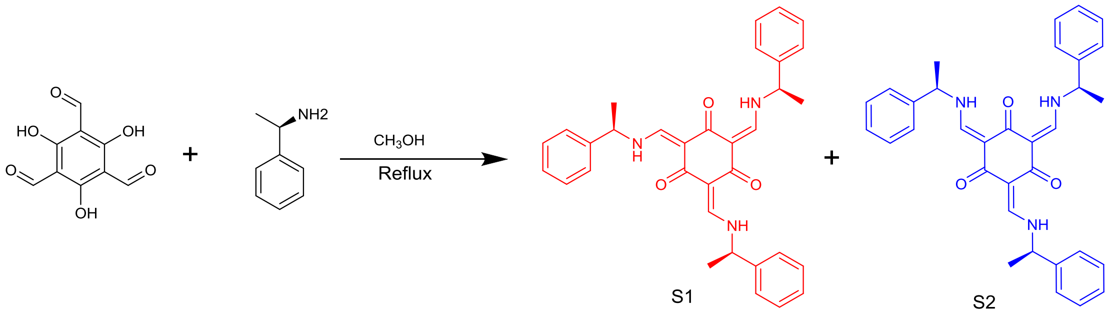

To a 25 mL round bottom flask, Tp (210 mg, 1 mmol), (*R*)-1-phenylethylamine (363 mg, 3 mmol) and CH_3OH (10mL) were added. The mixture was stirred and heated to reflux. After 24h, the reaction was cooled to room temperature, and the solvent was removed under vacuum to provide pale red oil. The oil was purified by column chromatography on silica gel with hexane/ethyl acetate (5:1 v/v) to give (*R*)-Tp-1-PEA as a mixture of isomers (**1** and **2**) (480 mg, 86% yield). ^{1}H NMR (400 MHz, $CDCl_3$) δ 12.00-11.16 (m, 3H), 8.48-8.01 (m, 3H), 7.52-7.07 (m, 5H), 4.88-4.42 (m, 3H), 1.80-1.49 (m, 9H). ^{13}C NMR (101 MHz, $CDCl_3$) δ 188.02, 185.33, 182.66, 156.59, 156.01, 155.68, 154.86, 141.95, 128.99, 127.91, 126.10, 104.78, 59.05, 23.10. ESI-MS: m/z 518.2438 (Calcd m/z 519.2522 for $[M-H]^-$).

Synthesis of model compound

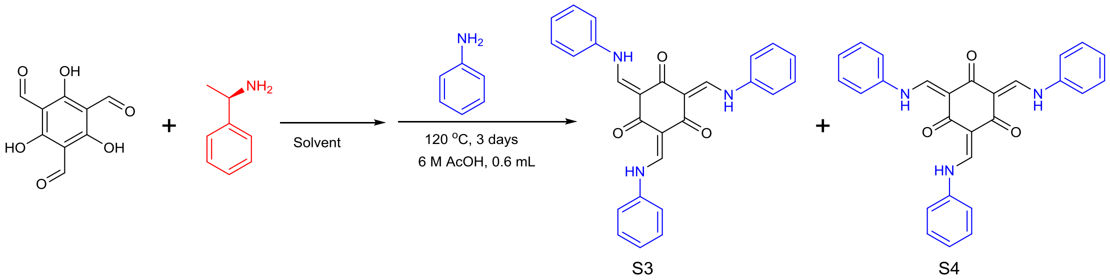

A mixture of Tp (32 mg, 0.15 mmol) and (*S*)- or (*R*)-1-PEA (18 mg, 0.15 mmol) was dissolved in 3 mL of mesitylene/dioxane (1:1 v/v) in a small vial and then the aniline (0.45 mmol) was added. After stirring for 15 min, 0.6 mL aqueous acetic acid (6M) was added, and the mixture was sonicated to afford a homogeneous dispersion. The solution was transferred into a Pyrex tube and degassed by three freeze-pump-thaw cycles. The tube was sealed off and heated at 120 °C for 3 days. After it was cooled to room temperture, the solution was concentrated under vacuum and 10 mL EtOH was added. The yellow precipitate was collected by centrifugation, washed with anhydrous THF (4 × 4 mL), and dried under vacuum to give model compound (47% yields). Characterization matched that reported in the literature[1].

Hydrolyzation of chiral COFs.

A mixture of the CCOF-TpPa-1 prepared by different crystallization time (2, 4, 12, 24 and 72 h) (12 mg), NaOH (40 mg), DMSO-*d*6 (1.0 mL) and D_2O (0.1 mL) was heated at 60 ^{o}C for 12 h under a nitrogen atmosphere. After cooling to room temperature, the solution was subjected to ^{1}H NMR spectroscopy analysis.

Cu Kα radiation X-ray diffraction and structure simulation.

Powder X-ray diffraction (PXRD) data were collected on a Bruker D8 Advance diffractometer using Cu Kα radiation at 40 kV, 40 mA power. Samples were observed using a continuous 2θ scan from 2-35 º. Because of their high symmetrical 2D layered structure, they have small number of discrete peaks which mainly appeared in $2\theta < 30^o$. No peaks could be resolved for $2\theta > 30^o$ and was therefore not considered for further analysis. The Pawley refinement of the experimental PXRD was conducted by the Reflux module in the Materials Studio 7.0. The simulated PXRD patterns were determined by the Reflex module. And the unit cell and the structure were optimized by Forcite module under molecular mechanics calculation using Dreiding as the forcefield to give the relative total energy.

High-resolution Synchrotron X-ray diffraction and structure simulation.

The high-resolution synchrotron X-ray diffraction (XRD) patterns were measured at BL14B1 of the Shanghai Synchrotron Radiation Facility (SSRF). The powdered samples were loaded into a 0.8 mm quartz capillary, which was continuously spinning during X-ray data collection. The Mythen 1K linear detector was adapted for the data collection in transmission mode. The λ is 0.689 Å and the step size is 0.0036°. The wavelength of the X-ray was calibrated using LaB_6 standard from NIST(660b). The measurement of the positions of the diffraction peaks was carried out using Galactic PeakSolveTM program, where experimental diffractograms are fitted using Gaussian shaped peaks. The crystal structure of CCOFs was simulated by Material Studio 7.0 according to the high-resolution PXRD.

The Tp-based COF is a crystal which structure is similar to the form of graphite layers. The formation of the eclipsed structure originates from the strong tendency for the hexagonal units to form coplanar aggregates which could stabilize the π-π stacking interactions between adjacent layers. Its regularity must be less than the single crystal that grown in the ideal environment. The tolerance of long range accumulation can

easily cause the peak to broaden. Moreover, most peaks underwent some broadening because COF crystallites have nanometer dimensions.

The initial structure models of the COFs were built using the Forcite module of the Materials Studio 7.0 software package. We applied the space group with the highest possible symmetry, i.e. *P*6, taking into account the propeller-like conformation of the TASN cores. Using this coarse model we determined the unit cell parameters via Le Bail refinement of our PXRD data. In order to obtain a more realistic picture of the local molecular arrangement, we then performed DFT geometry optimizations based on the refined unit cell parameters using the CASTEP module. These models with rigid bodies were suitable for subsequent Rietveld refinements.

The propeller-like structure of COFs has been proved by the synchrotron PXRD data in the literature. Besides, the chiral feature of TASN core has also been verified by the single crystal and DFT calculations. We believe the new results are enough to prove the chiral feature exist in those COFs and make the atomic coordinate extremely approach to the real value.

Time resolved decay traces of CCOF-TpTab in the presence of the D-cellobiose quencher.

2 mL of TpTab stock solution from the above was added to a quartz cuvette. Upon excitation by a λ = 387 nm laser source, emission decay at λ = 540 nm was recorded. The measurement was repeated after each addition of the quencher solution to give a series of emission decay traces. Fluorescence lifetime was measured using a lifetime spectrometer of Edinburgh Instrument (lifespec-red F900). The quantum yields of (Λ)-TpTab in PBS before and after addition of 140 μL of D-cellobiose (1.0×10^{-3} mol/L) were calculated by comparing the integrated photoluminescence intensities and the absorbency values of (Λ)-TpTab with the references quinine sulfate. Quinine sulfate (literature φ = 0.55) was dissolved in 0.5 M H_2SO_4 (refractive index (η) of 1.33). The (Λ)-TpTab samples were dispersed in PBS (η =1.33). A quartz cuvette with a path length of 1.00 cm was used to contain the samples during the UV-Vis and PL experiments. Excitation slit width of 4 nm and an emission slit width of 4 nm was used to excite the (Λ)-TpTab samples at 387 nm to record their photoluminescence spectra. The absorbency was controlled < 0.05 for the samples and quinine sulfate. The quantum yield was calculated using the below equation:

$$\varphi_S = \varphi_{ST}(0.55)(A_{ST}/A_S)(F_S/F_{ST})(\eta_S^2 / \eta_{ST}^2)$$

Where **φ** is the quantum yield, **A** is the absorbency, **F** is the integrated photoluminescence intensity, **η** is the refractive index of the solvent, **S** is sample, **ST**

is the standard of Quinine Sulfate (φ= 0.55). Based on the equation, the quantum yields are calculated as 2.00 % and 1.12% for (*Λ*)-TpTab before and after addition of 140 μL of D-cellobiose (1.0 $\times 10^{-3}$ mol/L), respectively.

The X-ray absorption fine structure (XAFS**).**

For the XAFS part, the Fourier transformed (FT) data in *R* space were analyzed by Cu-O/N contributions. The parameters describing the electronic properties (e.g., correction to the photoelectron energy origin, E_0) and local structure environment including *CN*, bond distance (*R*) and Debye-Waller (*D.W.*) factor around the absorbing atoms were allowed to vary during the fit process. The scattering path distances and degeneracies derived from these fits are consistent with tetrahedral coordination of the Cu centers with two O atoms of the acetate, one O and one N atoms of chiral TpTab.

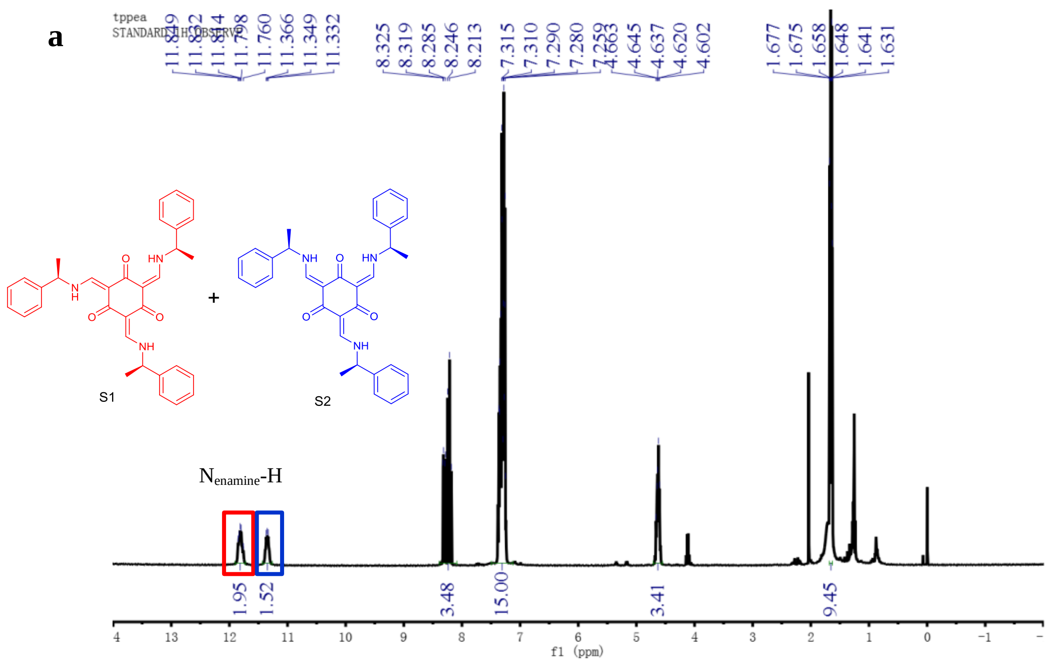

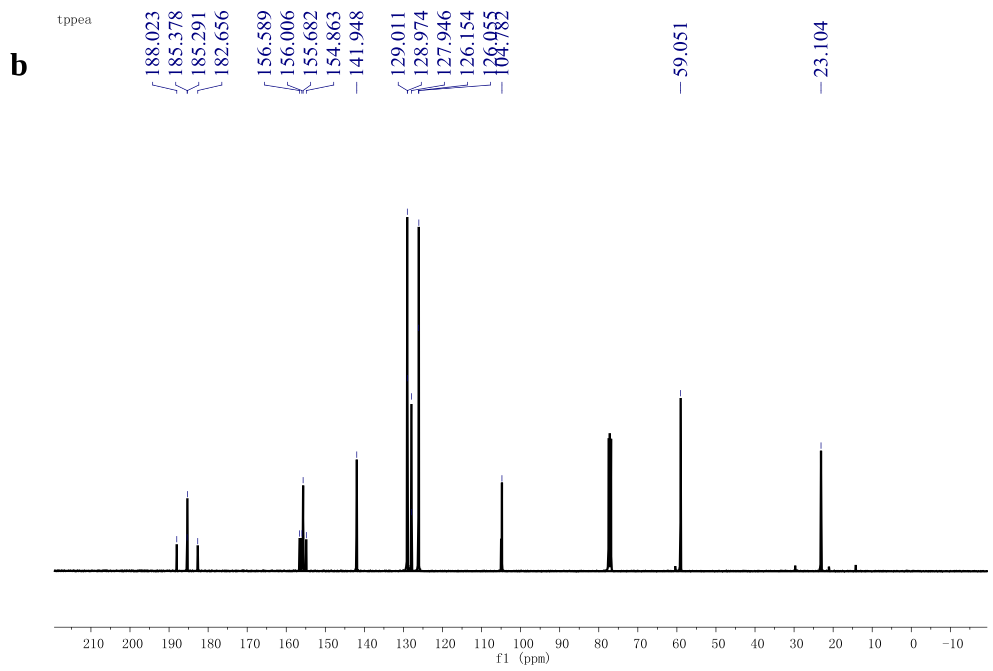

Supplementary Figure 1. NMR spectra. a. ^{1}H NMR spectrum of the (*R*)-Tp-1-PEA. **b.** ^{13}C NMR spectrum of the (*R*)-Tp-1-PEA.

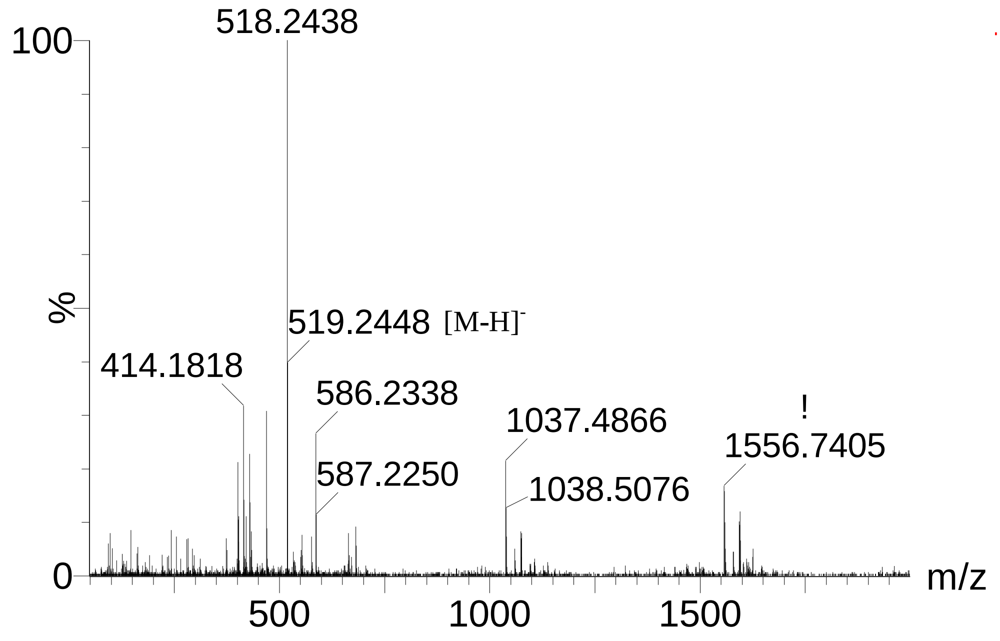

Supplementary Figure 2. ESI-MS of (*R)*-Tp-1-PEA.

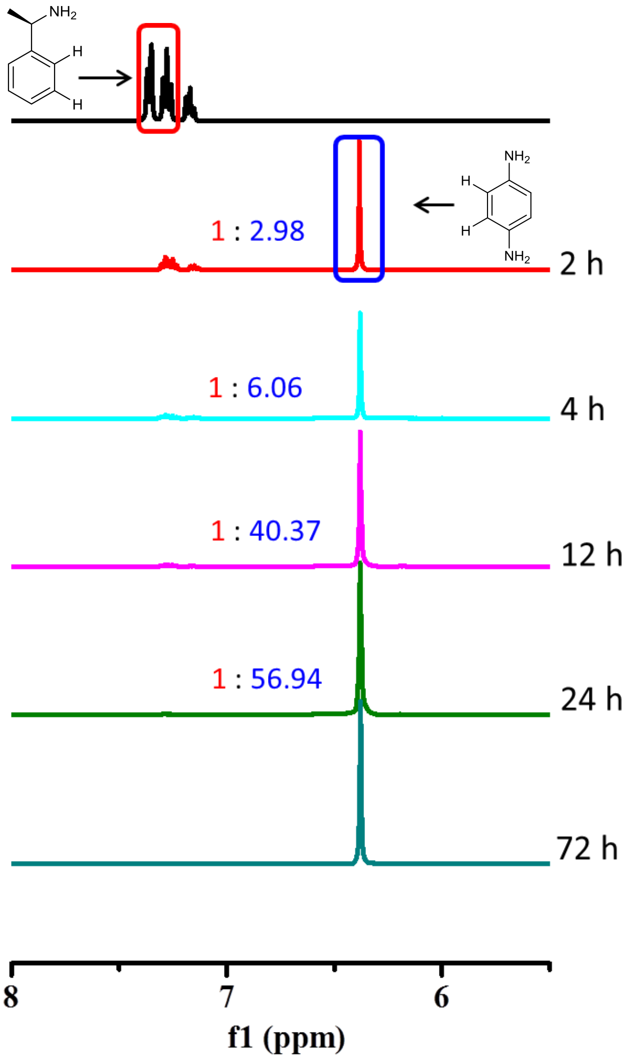

Supplementary Figure 3. **Partial ^{1}H NMR (400 MHz, DMSO-*d6*) spectra of the hydrolyzed samples.**

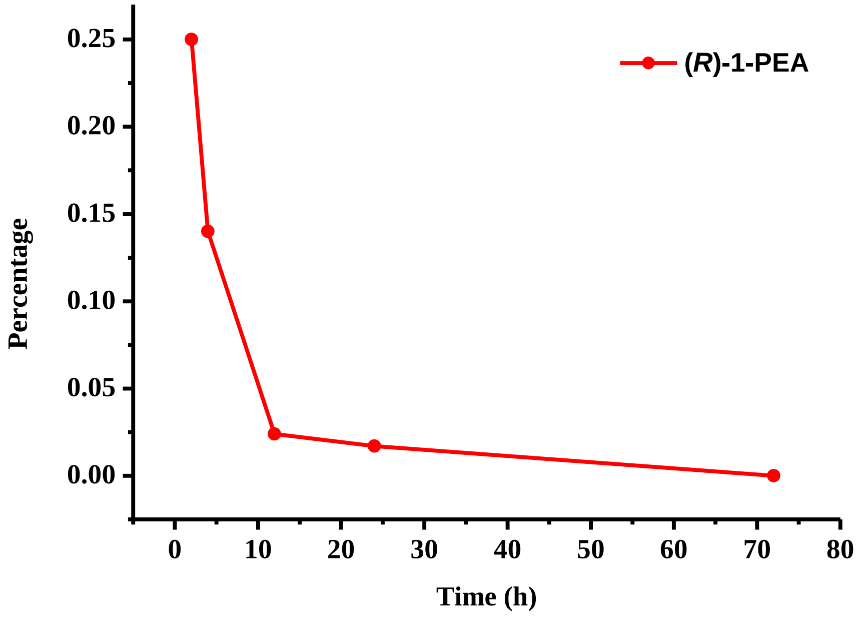

Supplementary Figure 4. Plot of molar percentages of (*R*)-1-PEA in the insoluble materials obtained at different transformation time intervals.

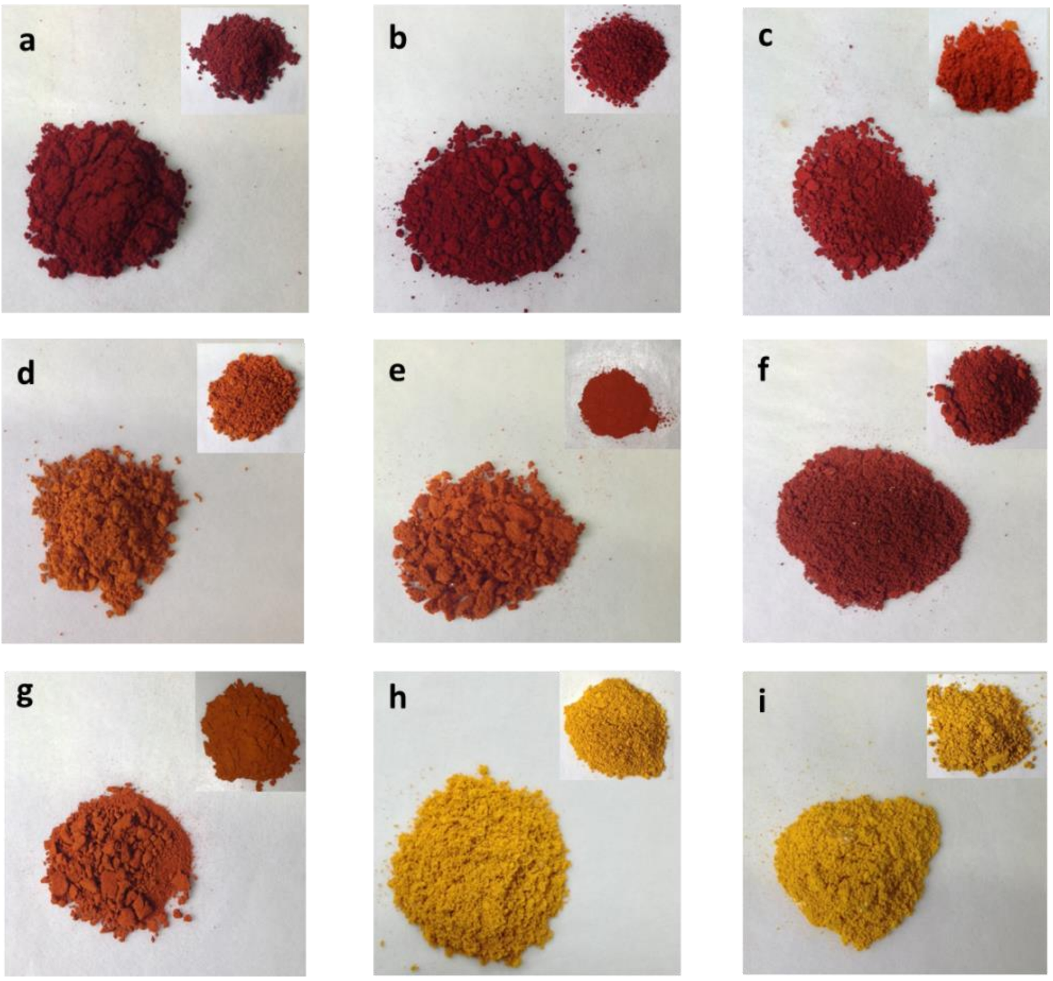

Supplementary Figure 5. a-i, The as-synthesized samples of CCOFs TpPa-1, TpPa-2, TpPa-Py, TpBD, TpBD-Me_2, TpBD-$(OMe)_2$, TpBpy ,TpTd and TpTab, respectively. Insert: the corresponding achiral COFs.

a

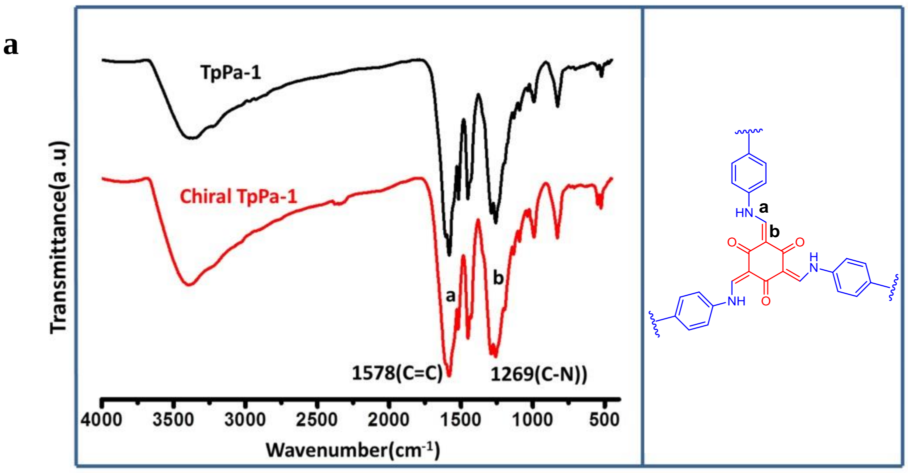
TpPa-1
Chiral TpPa-1
a
b
1578(C=C)
1269(C-N))
Transmittance(a.u)
4000
3500
3000
2500
2000
1500
1000
500
Wavenumber(cm-1)
HN
NH
O

b

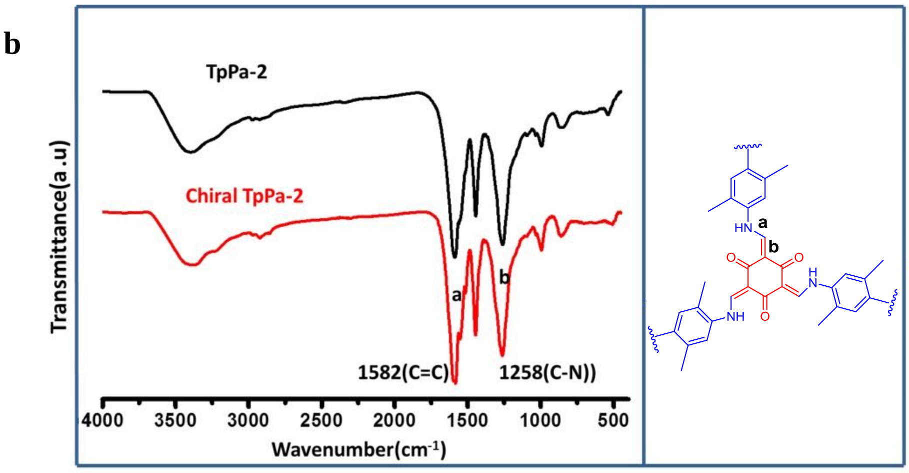
TpPa-2
Chiral TpPa-2
a
b
1582(C=C)
1258(C-N))
Transmittance(a.u)
4000
3500
3000
2500
2000
1500
1000
500
Wavenumber(cm-1)
HN
NH
O

c

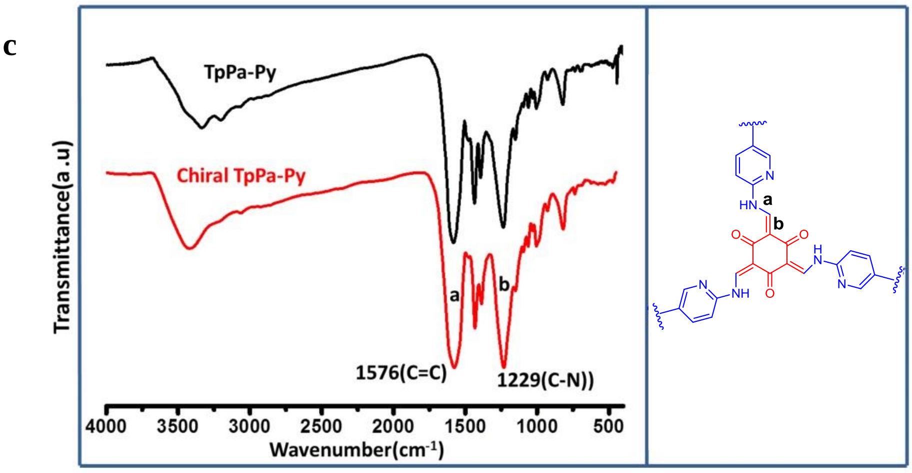
TpPa-Py
Chiral TpPa-Py
a
b
1576(C=C)
1229(C-N))
Transmittance(a.u)
4000
3500
3000
2500
2000
1500
1000
500
Wavenumber(cm-1)
HN
NH
N
O

d

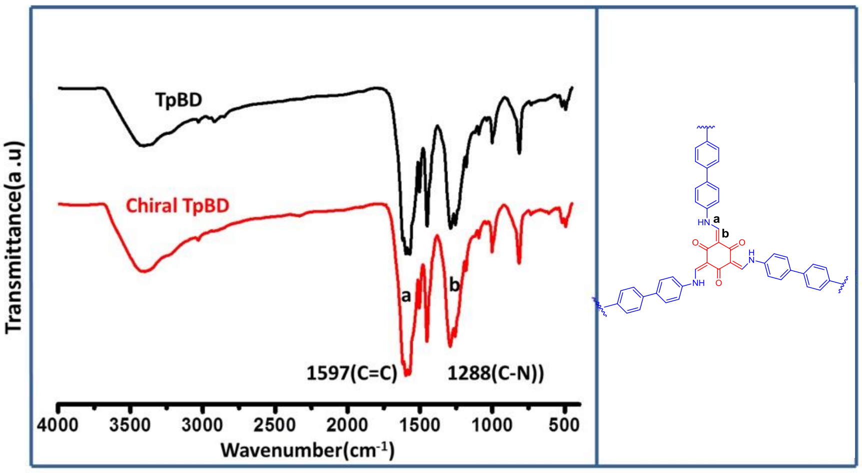
TpBD
Chiral TpBD
Transmittance(a.u)
a
b
1597(C=C)
1288(C-N))
4000 3500 3000 2500 2000 1500 1000 500
Wavenumber(cm-1)
HN
NH
a
b

e

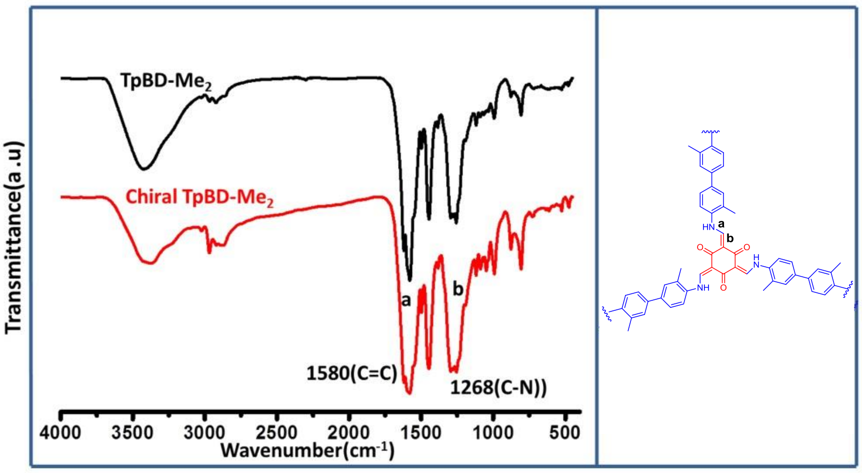
TpBD-Me2
Chiral TpBD-Me2
Transmittance(a.u)
a
b
1580(C=C)
1268(C-N))
4000 3500 3000 2500 2000 1500 1000 500
Wavenumber(cm-1)
HN
NH
a
b

f

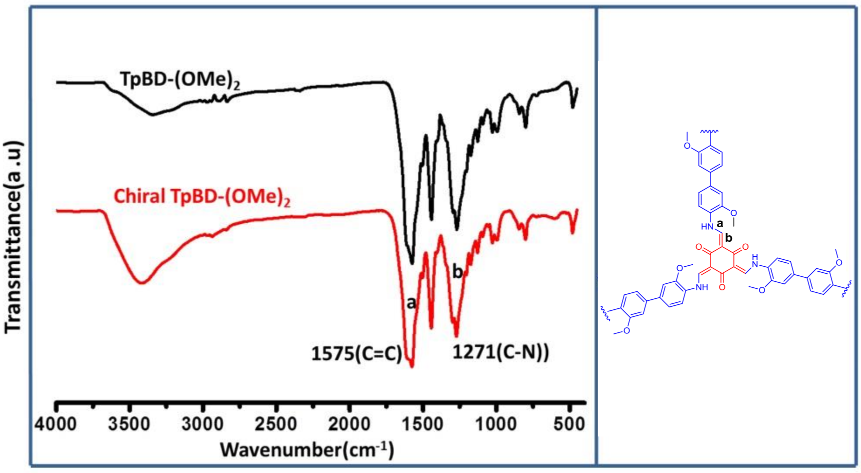
TpBD-(OMe)2
Chiral TpBD-(OMe)2
Transmittance(a.u)
a
b
1575(C=C)
1271(C-N))
4000 3500 3000 2500 2000 1500 1000 500
Wavenumber(cm-1)
HN
NH
a
b

g

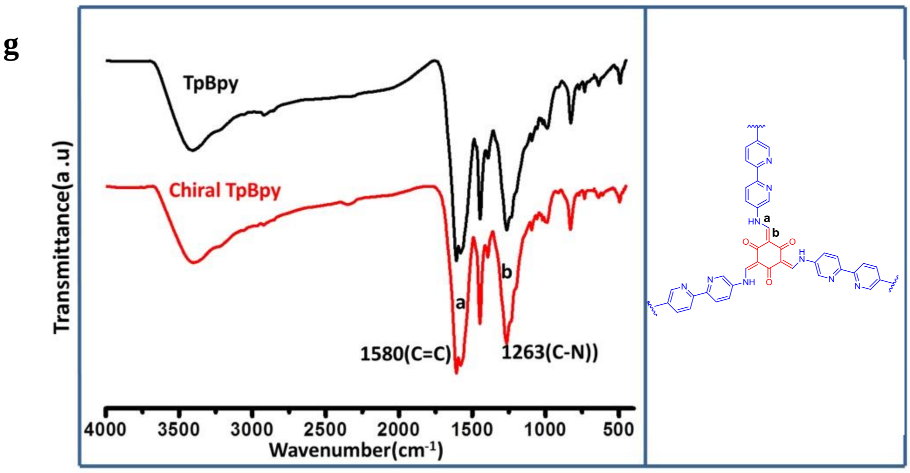

h

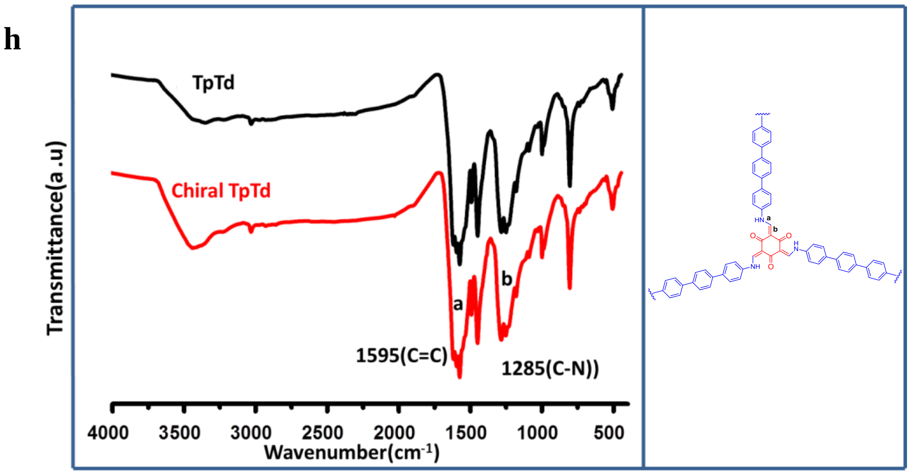

i

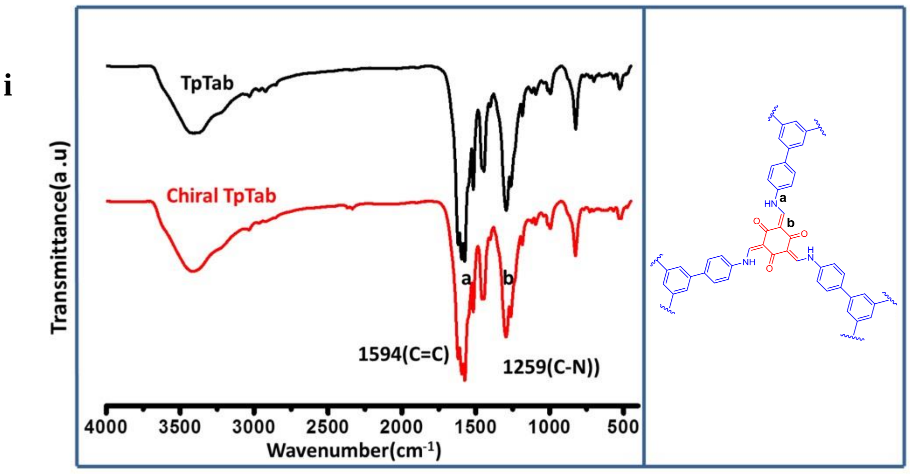

Supplementary Figure 6. FT-IR spectra of COFs. a-i, The IR spectra of chiral and achiral TpPa-1, TpPa-2, TpPa-Py, TpBD, TpBD-Me_2, TpBD-$(OMe)_2$, TpBpy ,TpTd and TpTab, respectively.

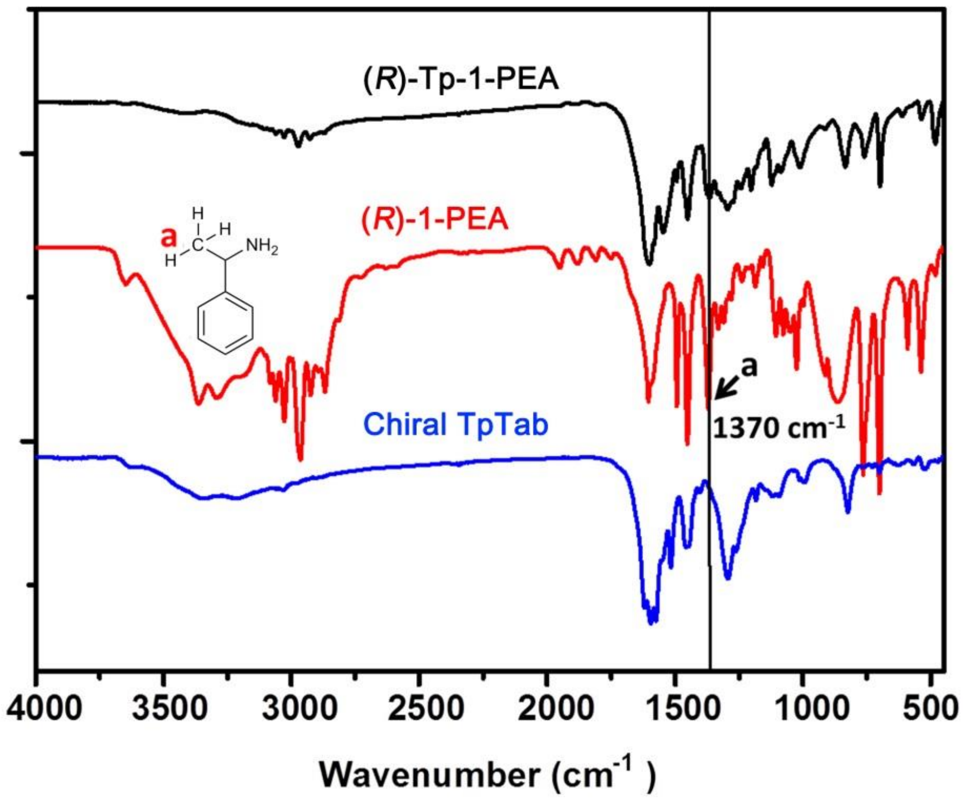

Supplementary Figure 7. FT-IR spectra of (*R*)-Tp-1-PEA, (*R*)-1-PEA and chiral TpTab.

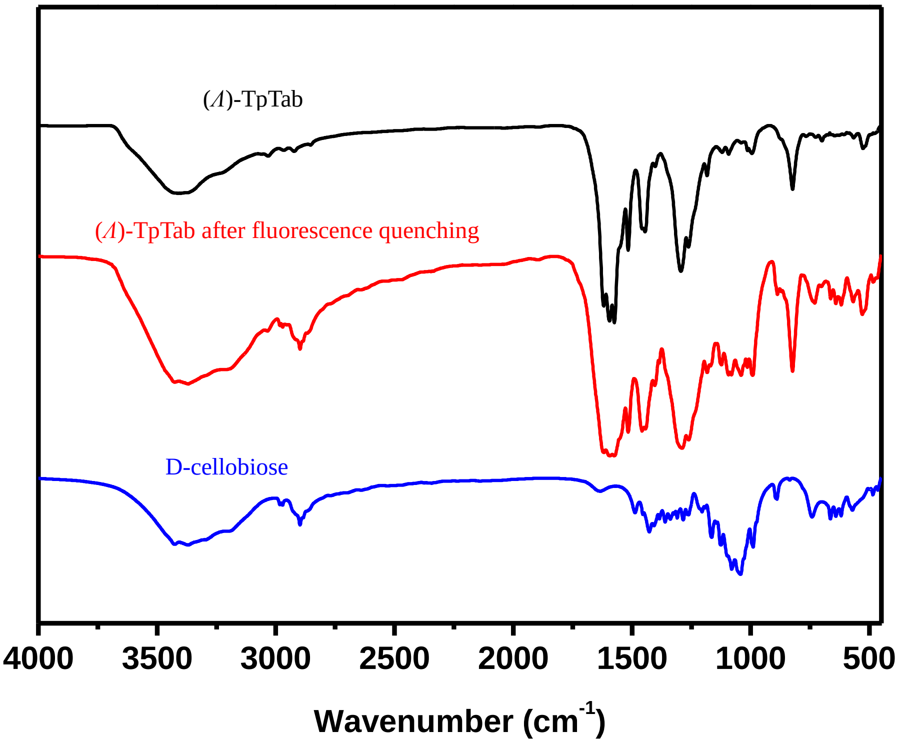

Supplementary Figure 8. FT-IR spectra of (Λ)-TpTab after fluorescence quenching experiments.

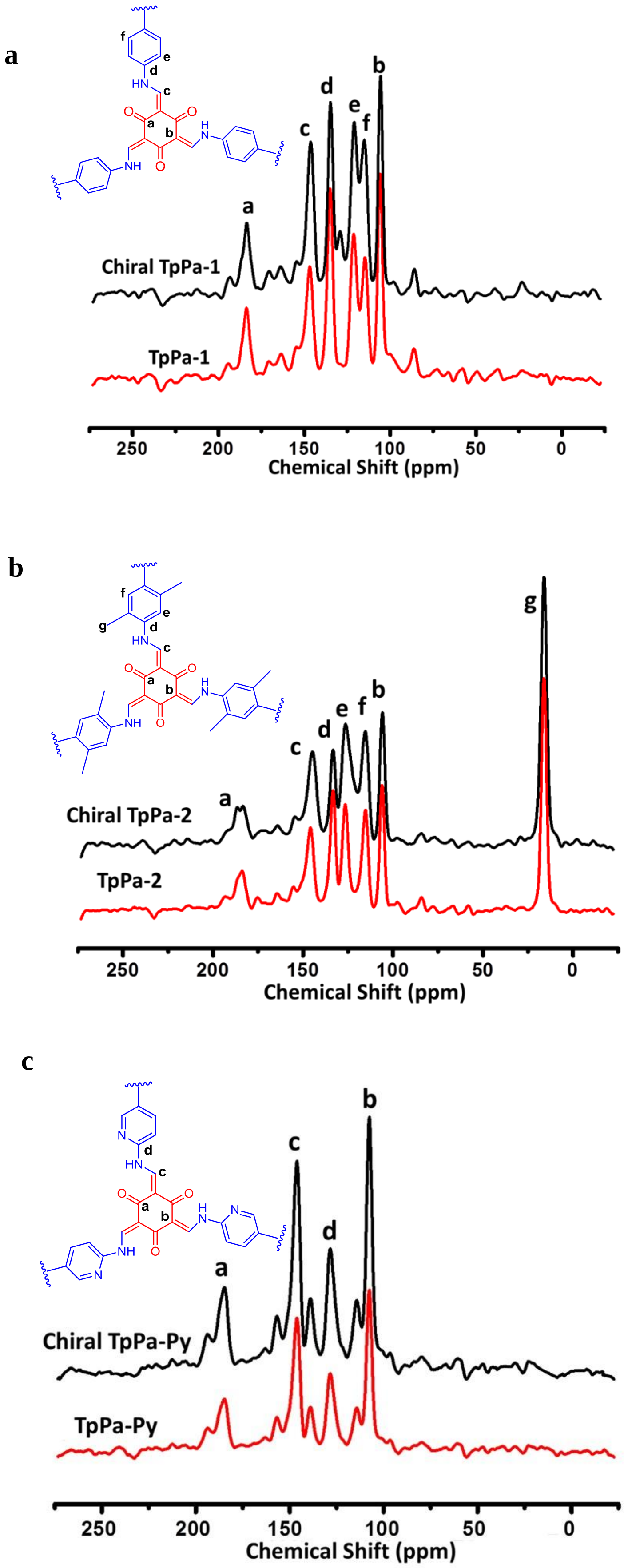
a
Chiral TpPa-1
TpPa-1
Chemical Shift (ppm)
b
Chiral TpPa-2
TpPa-2
Chemical Shift (ppm)
c
Chiral TpPa-Py
TpPa-Py
Chemical Shift (ppm)

d

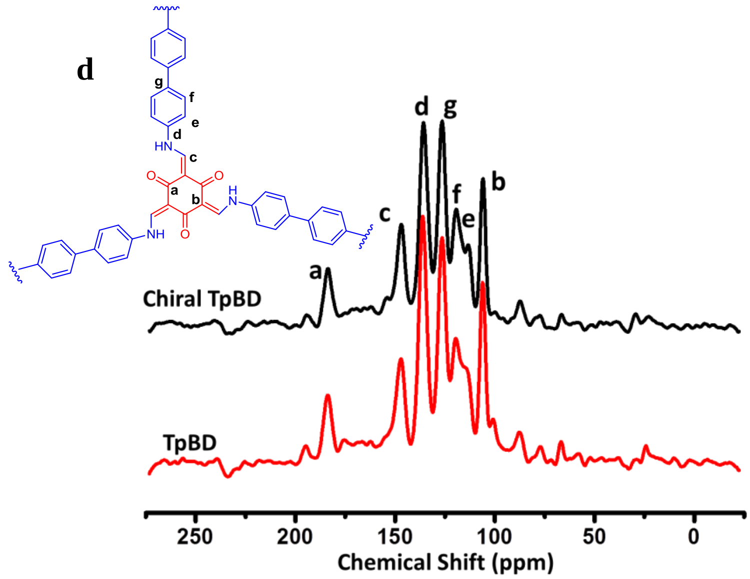
Chiral TpBD
TpBD
Chemical Shift (ppm)

e

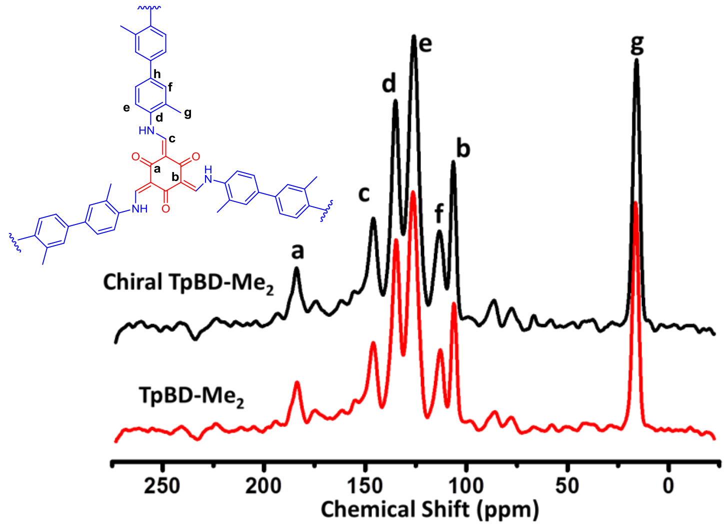
Chiral TpBD-Me2
TpBD-Me2
Chemical Shift (ppm)

f

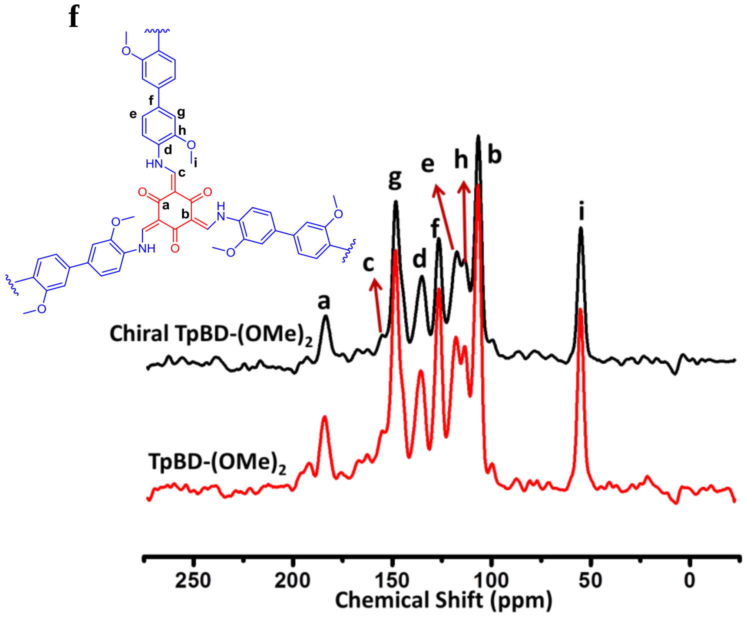
Chiral TpBD-(OMe)2
TpBD-(OMe)2
Chemical Shift (ppm)

g

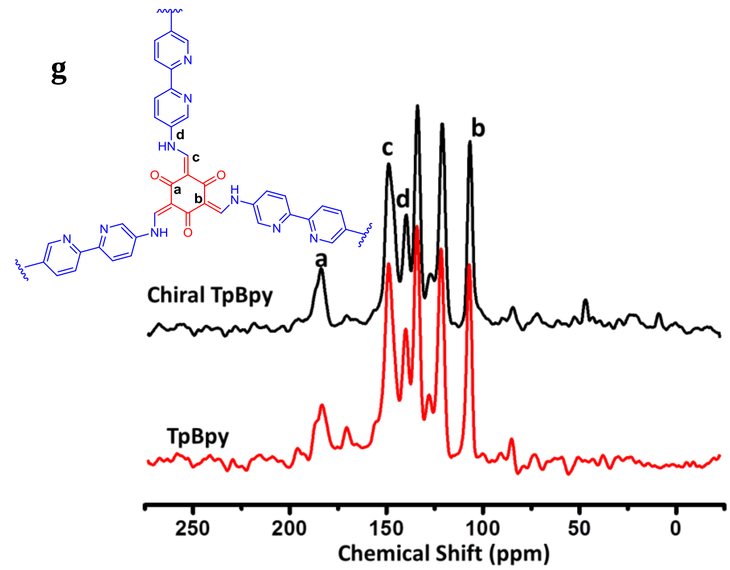

c
d
b
a
Chiral TpBpy
TpBpy
250
200
150
100
50
0
Chemical Shift (ppm)

h

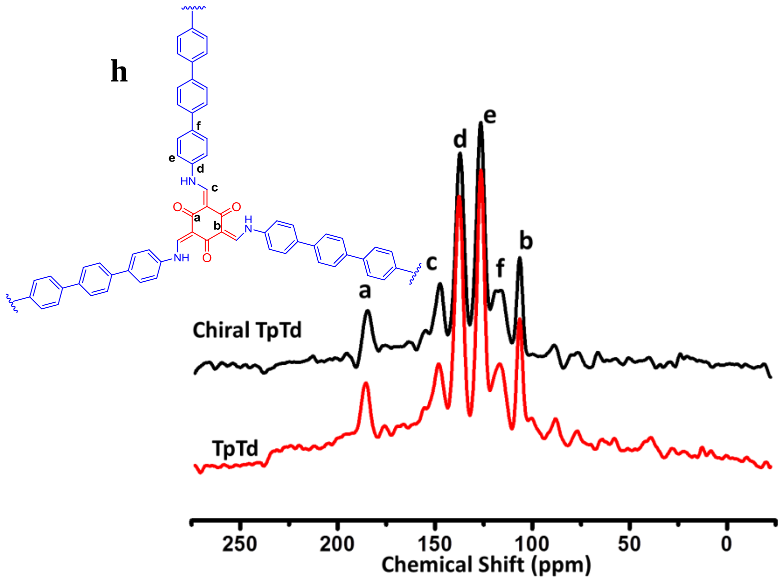

d
e
c
f
b
a
Chiral TpTd
TpTd
250
200
150
100
50
0
Chemical Shift (ppm)

i

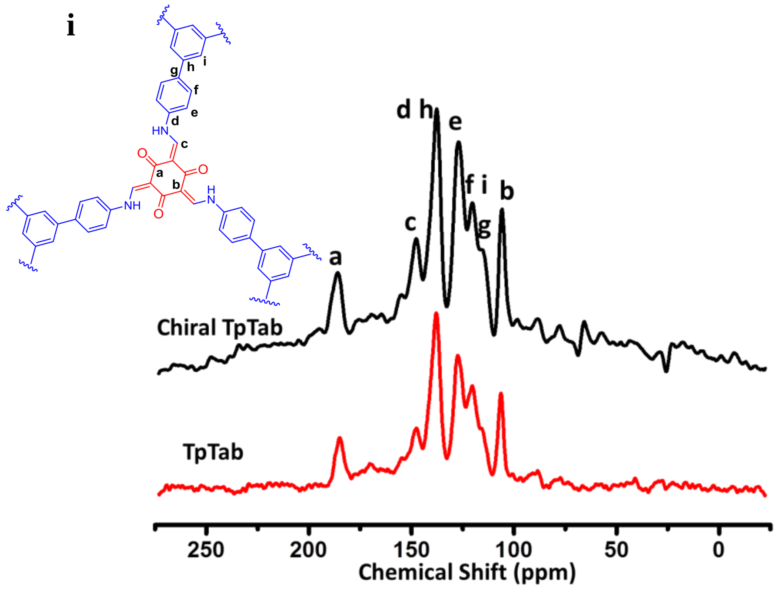

d h
e
f i
g
b
c
a
Chiral TpTab
TpTab
250
200
150
100
50
0
Chemical Shift (ppm)

j

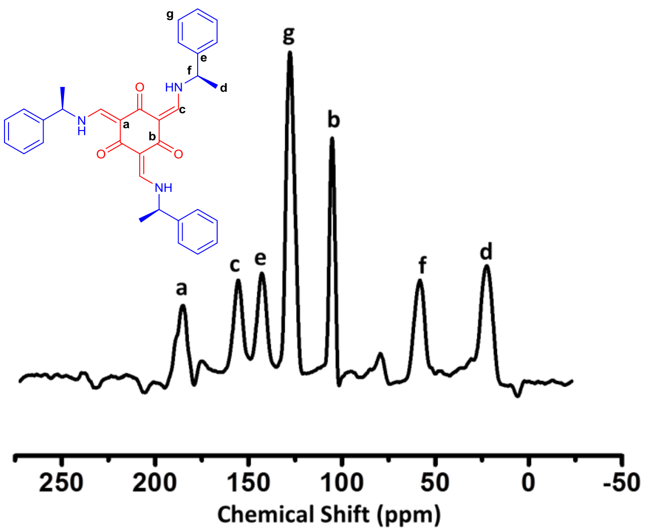

Supplementary Figure 9. ^{13}**C CP-MAS spectra. a-i**, The ^{13}C CP-MAS spectra of chiral and achiral TpPa-1, TpPa-2, TpPa-Py, TpBD, TpBD-Me_2, TpBD-$(OMe)_2$, TpBpy ,TpTd and TpTab, respectively. **j**, The ^{13}C CP-MAS spectrum of Tp-1-PEA.

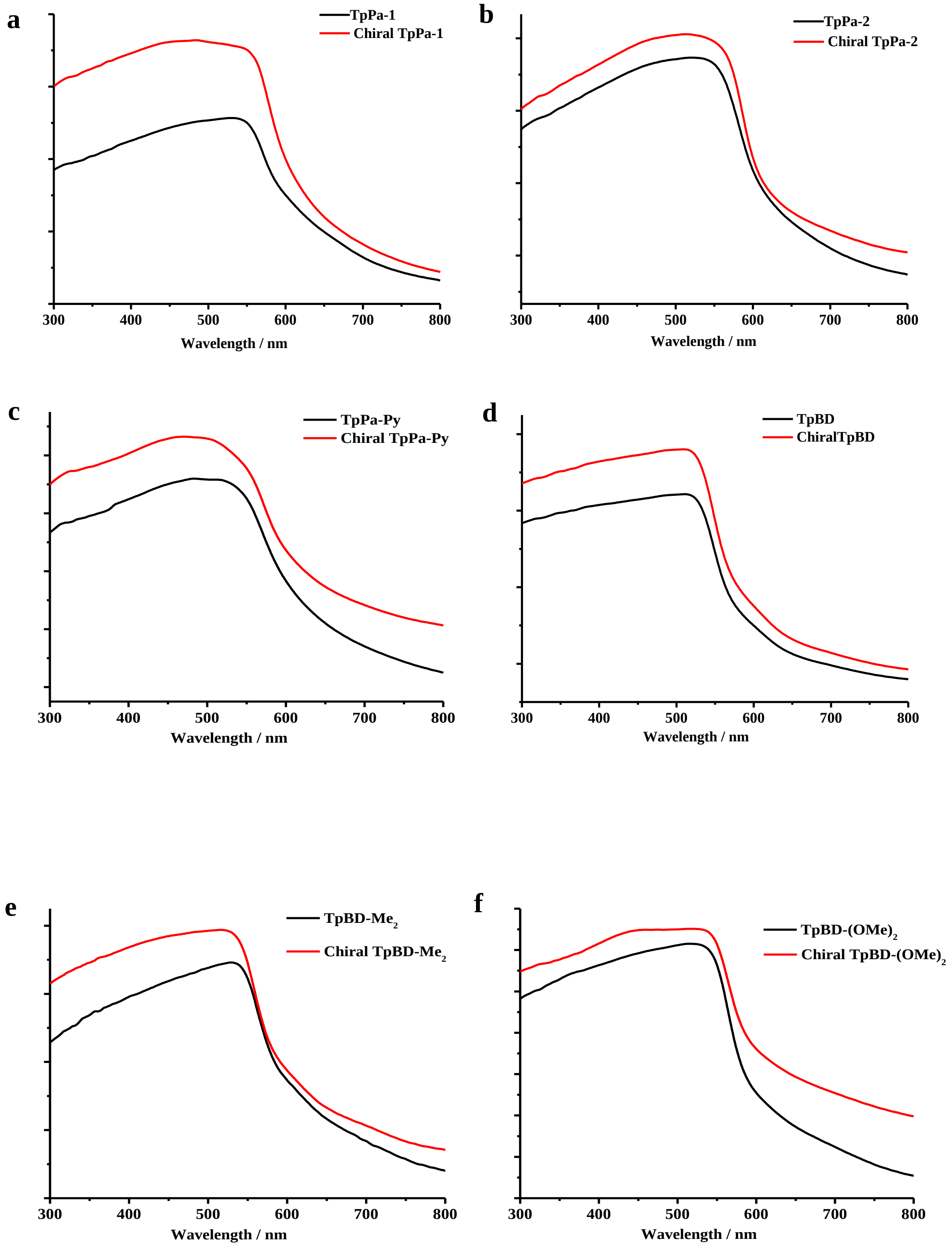
a
TpPa-1
Chiral TpPa-1
300 400 500 600 700 800
Wavelength / nm
b
TpPa-2
Chiral TpPa-2
300 400 500 600 700 800
Wavelength / nm
c
TpPa-Py
Chiral TpPa-Py
300 400 500 600 700 800
Wavelength / nm
d
TpBD
ChiralTpBD
300 400 500 600 700 800
Wavelength / nm
e
TpBD-Me_2
Chiral TpBD-Me_2
300 400 500 600 700 800
Wavelength / nm
f
TpBD-$(OMe)_2$
Chiral TpBD-$(OMe)_2$
300 400 500 600 700 800
Wavelength / nm

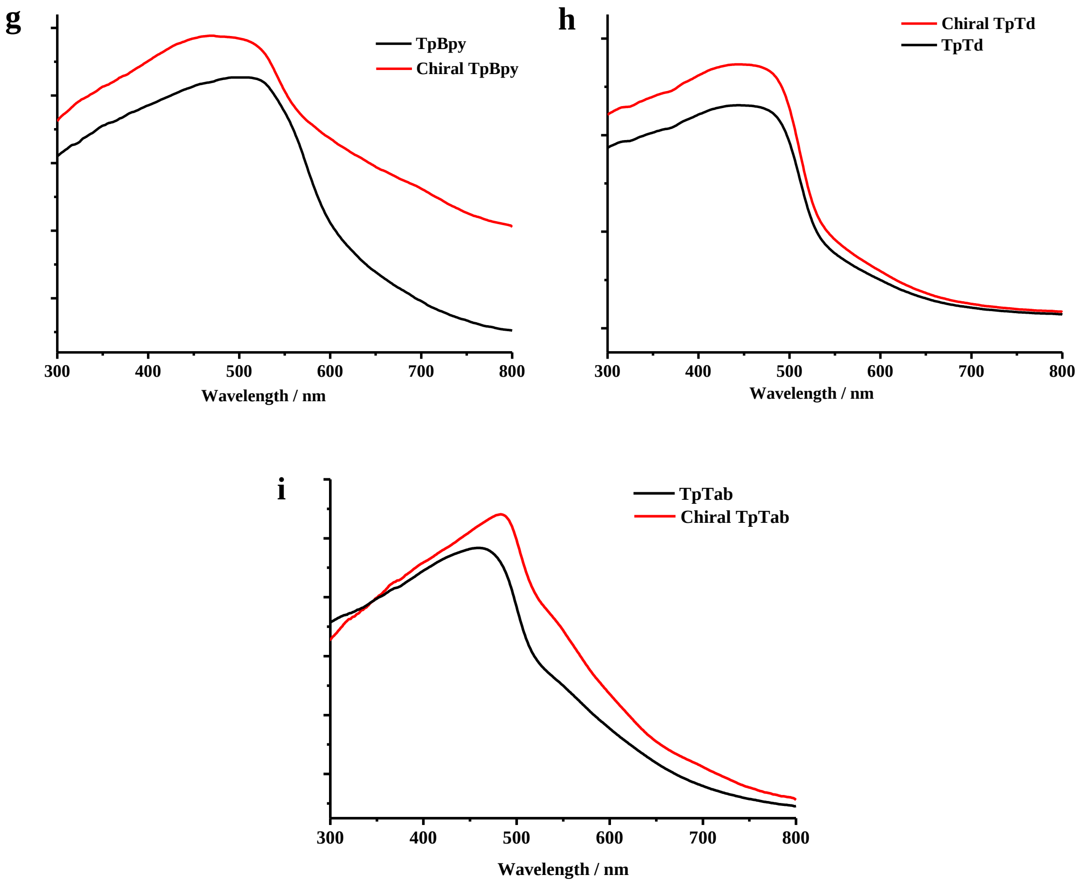

Supplementary Figure 10. The solid-state UV spectra of achiral and chiral COFs. The solid-state UV spectra of chiral and achiral TpPa-1, TpPa-2, TpPa-Py, TpBD, TpBD-Me_2, TpBD-$(OMe)_2$, TpBpy ,TpTd and TpTab, respectively.

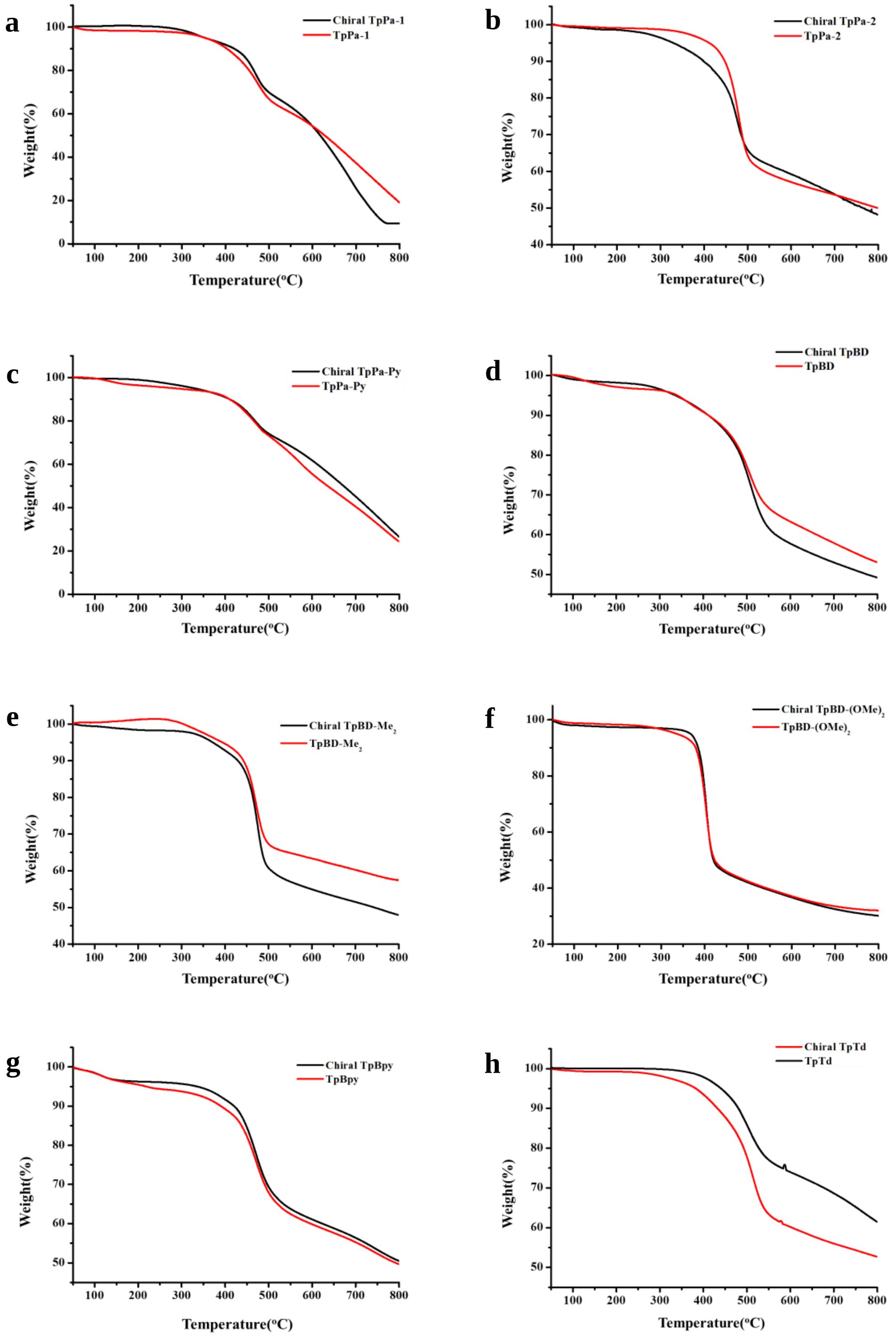
a
Chiral TpPa-1
TpPa-1
Weight(%)
Temperature(°C)
b
Chiral TpPa-2
TpPa-2
Weight(%)
Temperature(°C)
c
Chiral TpPa-Py
TpPa-Py
Weight(%)
Temperature(°C)
d
Chiral TpBD
TpBD
Weight(%)
Temperature(°C)
e
Chiral TpBD-Me$_2$
TpBD-Me$_2$
Weight(%)
Temperature(°C)
f
Chiral TpBD-(OMe)$_2$
TpBD-(OMe)$_2$
Weight(%)
Temperature(°C)
g
Chiral TpBpy
TpBpy
Weight(%)
Temperature(°C)
h
Chiral TpTd
TpTd
Weight(%)
Temperature(°C)

i

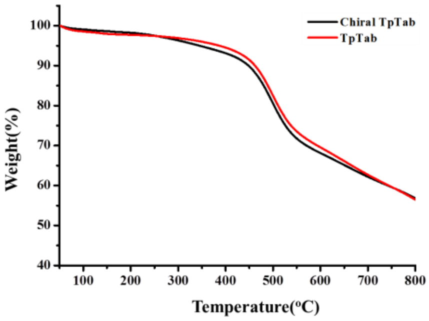

Supplementary Figure 11. TGA of achiral and chiral COFs. TGA of chiral and achiral TpPa-1, TpPa-2, TpPa-Py, TpBD, TpBD-Me_2, TpBD-$(OMe)_2$, TpBpy ,TpTd and TpTab, respectively.

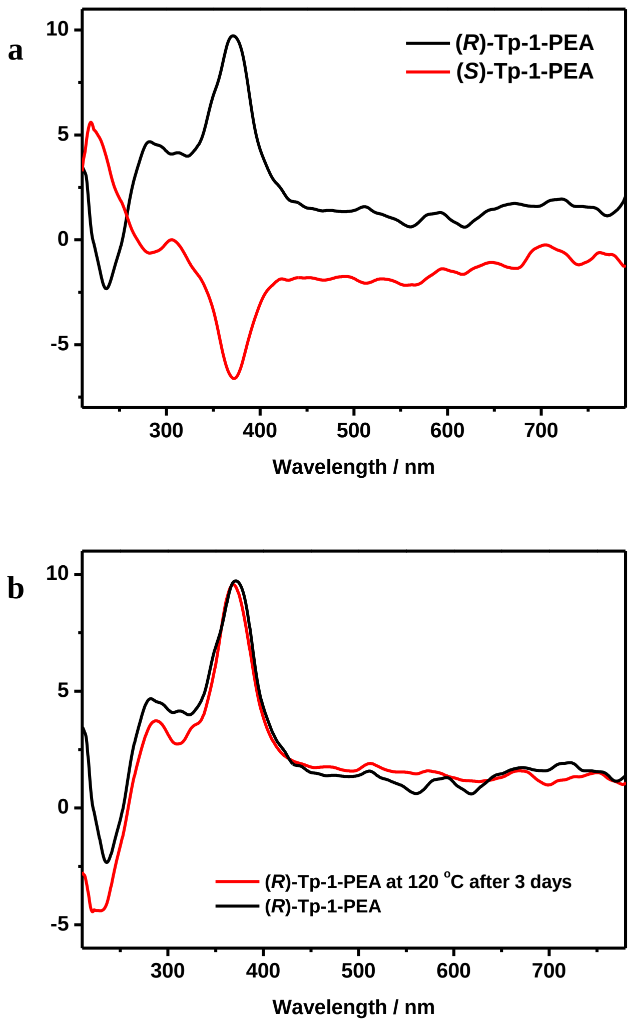

Supplementary Figure 12. The solid-state CD spectra. a, (*R*/*S*)-Tp-1-PEA. **b,** (*R*)-Tp-1-PEA at 120 °C after 3 days.

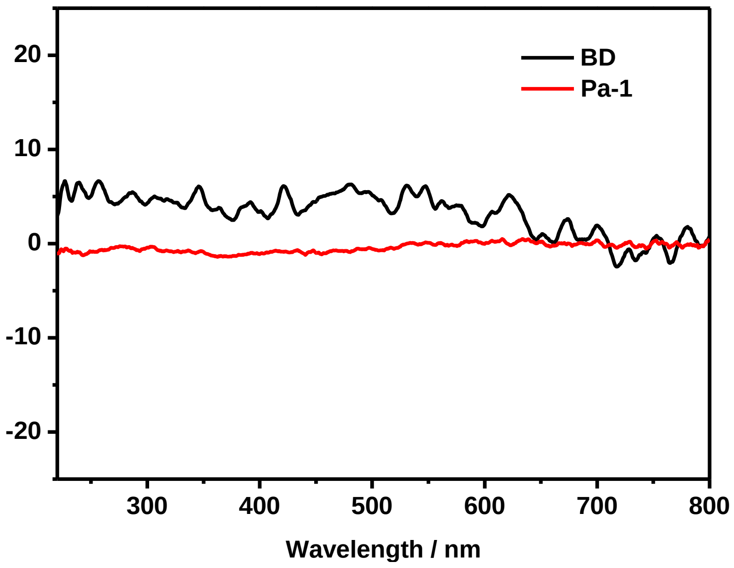

Supplementary Figure 13. The solid-state CD spectra of achiral COFs that synthesized from 1, 3, 5-triformylbenzene and Pa-1 or BD in the presence of (*R*)-1-PEA.

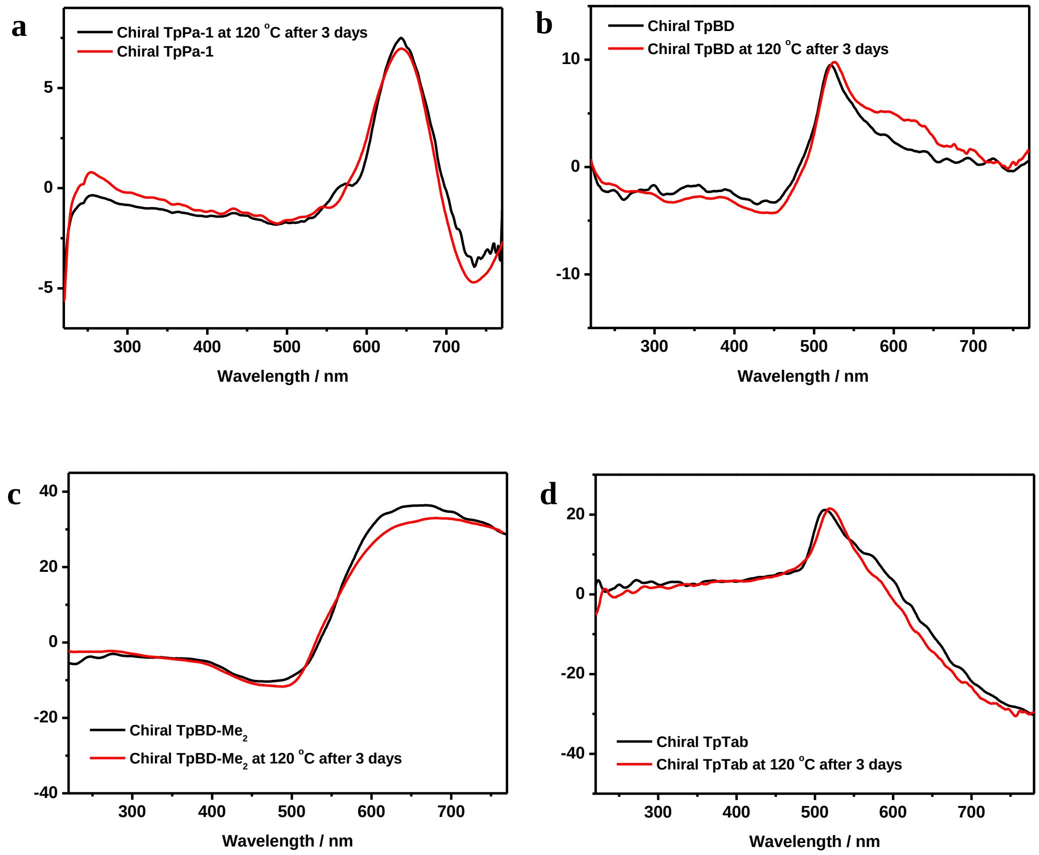

Supplementary Figure 14. The solid-state CD spectra of chiral COFs at 120 °C after 3 days. a, Chiral TpPa-1; **b**, Chiral TpBD; **c**, Chiral TpBD-Me_2; **d**, Chiral TpTab.

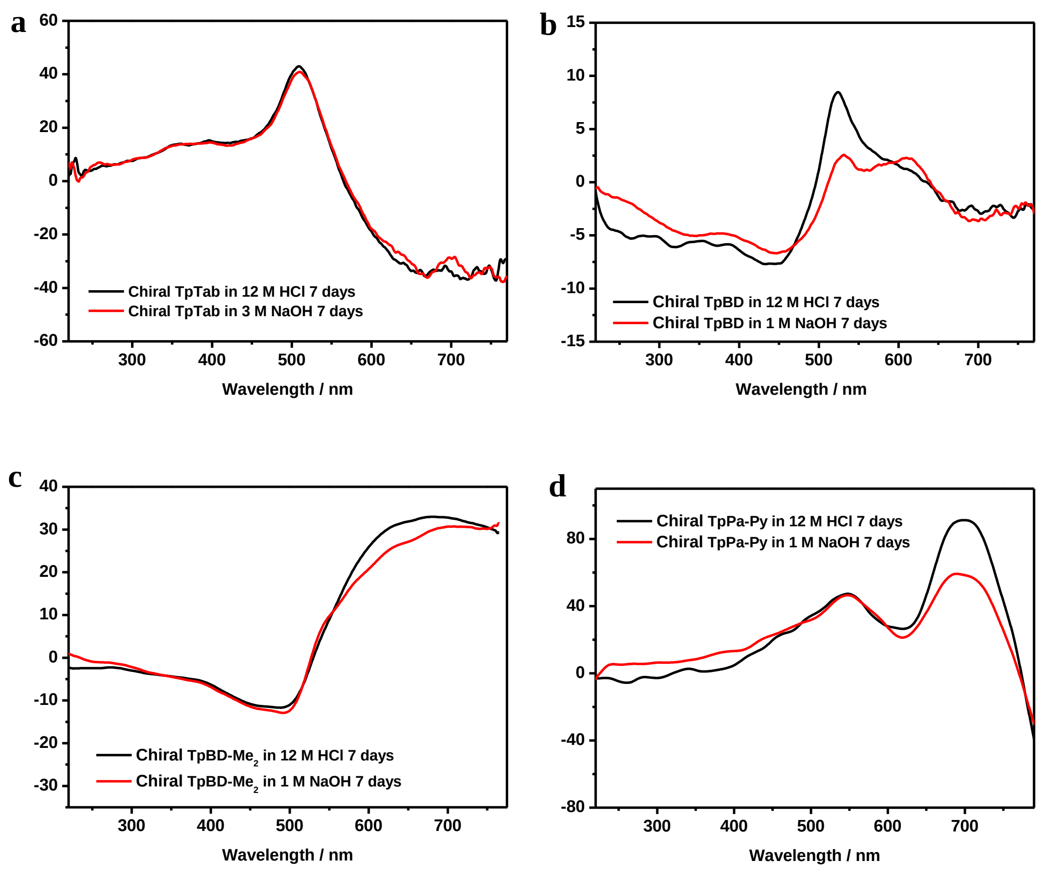

Supplementary Figure 15. The solid-state CD spectra of chiral COFs after treated by 12M HCl or 1 M NaOH 7 days. **a**, Chiral TpTab; **b**, Chiral TpBD; **c**, Chiral TpBD-Me_2; **d**, Chiral TpPa-Py.

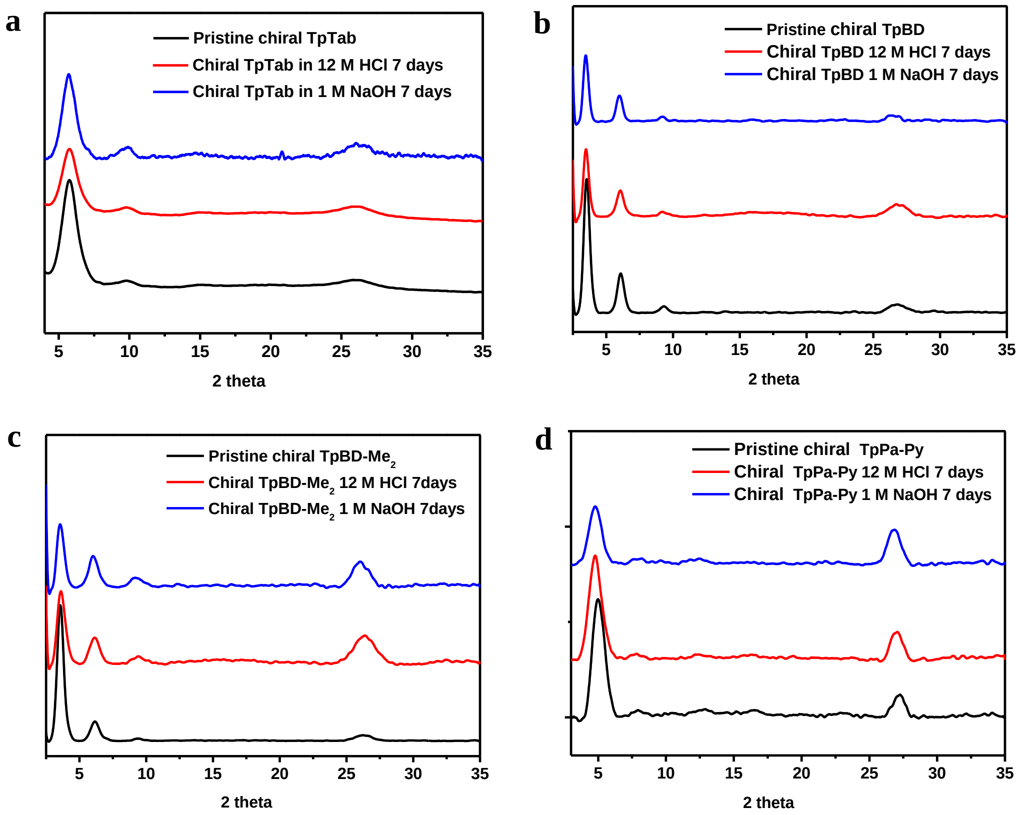

Supplementary Figure 16. The PXRD of chiral COFs after treated by 12M HCl or 1 M NaOH 7 days. **a**, Chiral TpTab; **b**, Chiral TpBD; **c**, Chiral TpBD-Me_2; **d**, Chiral TpPa-Py.

Supplementary Table 1. The optical rotation of (*R*)-1-PEA at 120 ^{o}C after 3 days.

Run	1	2	3	4	5	mean value	[a] / deg cm^{3} dm^{-1} g^{-1}
(*R*)-1-PEA	0.827^{o}	0.826^{o}	0.824^{o}	0.826^{o}	0.830^{o}	0.827^{o}	34.17
(*R*)-1-PEA after 120 ^{o}C 3 days	0.819^{o}	0.818^{o}	0.813^{o}	0.817^{o}	0.816^{o}	0.817^{o}	33.76

[a] = 100 a / l * c   T = 23 ^{o}C

c = 2.42 g/100mL

Supplementary Table 2. Calculated Total energies for the COFs structures by Forcite Tools.

	Chiral structure (kcal / mol)	Achiral structure (kcal / mol)
**TpPa-1**	110.3	146.1
**TpPa-2**	251.2	315.0
**TpPa-Py**	94.3	163.4
**TpBD**	218.3	299.8
**TpBD-Me_2**	464.7	1933.2
**TpBD-$(OMe)_2$**	705.0	1229.9
**TpBpy**	153.5	177.0
**TpTd**	202.5	233.1
**TpTab**	222.7	270.52

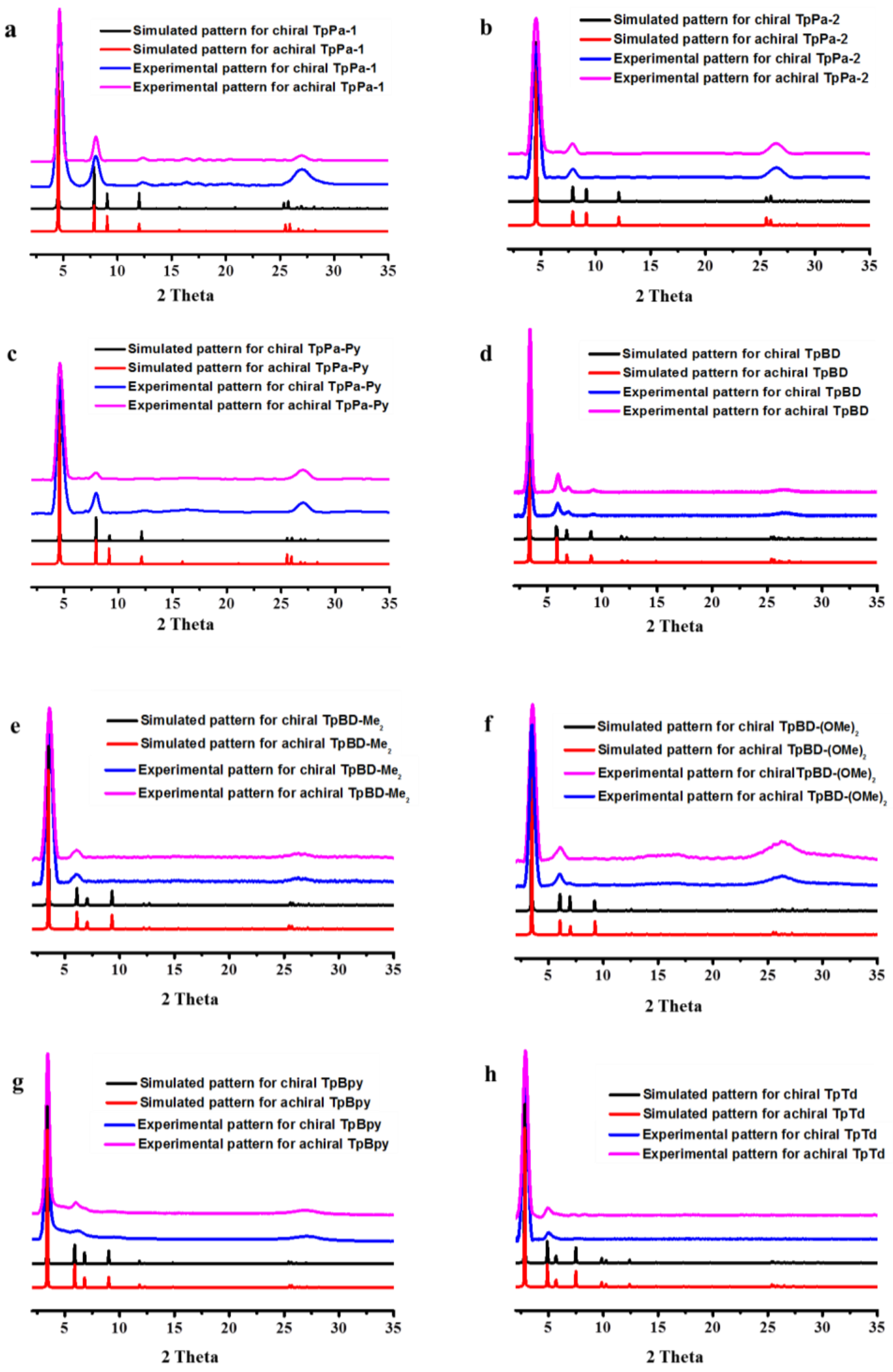
a
Simulated pattern for chiral TpPa-1
Simulated pattern for achiral TpPa-1
Experimental pattern for chiral TpPa-1
Experimental pattern for achiral TpPa-1
2 Theta
b
Simulated pattern for chiral TpPa-2
Simulated pattern for achiral TpPa-2
Experimental pattern for chiral TpPa-2
Experimental pattern for achiral TpPa-2
2 Theta
c
Simulated pattern for chiral TpPa-Py
Simulated pattern for achiral TpPa-Py
Experimental pattern for chiral TpPa-Py
Experimental pattern for achiral TpPa-Py
2 Theta
d
Simulated pattern for chiral TpBD
Simulated pattern for achiral TpBD
Experimental pattern for chiral TpBD
Experimental pattern for achiral TpBD
2 Theta
e
Simulated pattern for chiral TpBD-Me2
Simulated pattern for achiral TpBD-Me2
Experimental pattern for chiral TpBD-Me2
Experimental pattern for achiral TpBD-Me2
2 Theta
f
Simulated pattern for chiral TpBD-(OMe)2
Simulated pattern for achiral TpBD-(OMe)2
Experimental pattern for chiralTpBD-(OMe)2
Experimental pattern for achiral TpBD-(OMe)2
2 Theta
g
Simulated pattern for chiral TpBpy
Simulated pattern for achiral TpBpy
Experimental pattern for chiral TpBpy
Experimental pattern for achiral TpBpy
2 Theta
h
Simulated pattern for chiral TpTd
Simulated pattern for achiral TpTd
Experimental pattern for chiral TpTd
Experimental pattern for achiral TpTd
2 Theta

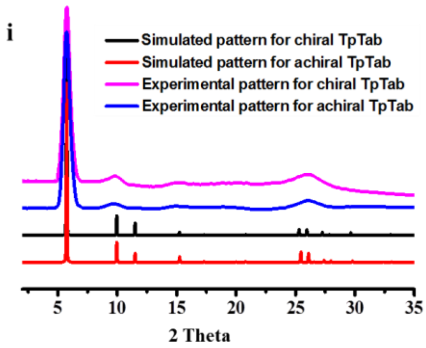

Supplementary Figure 17. The simulated and experimental PXRD of chiral and achiral COFs. a-i, TpPa-1, TpPa-2, TpPa-Py, TpBD, TpBD-Me_2, TpBD-$(OMe)_2$, TpBpy ,TpTd and TpTab, respectively.

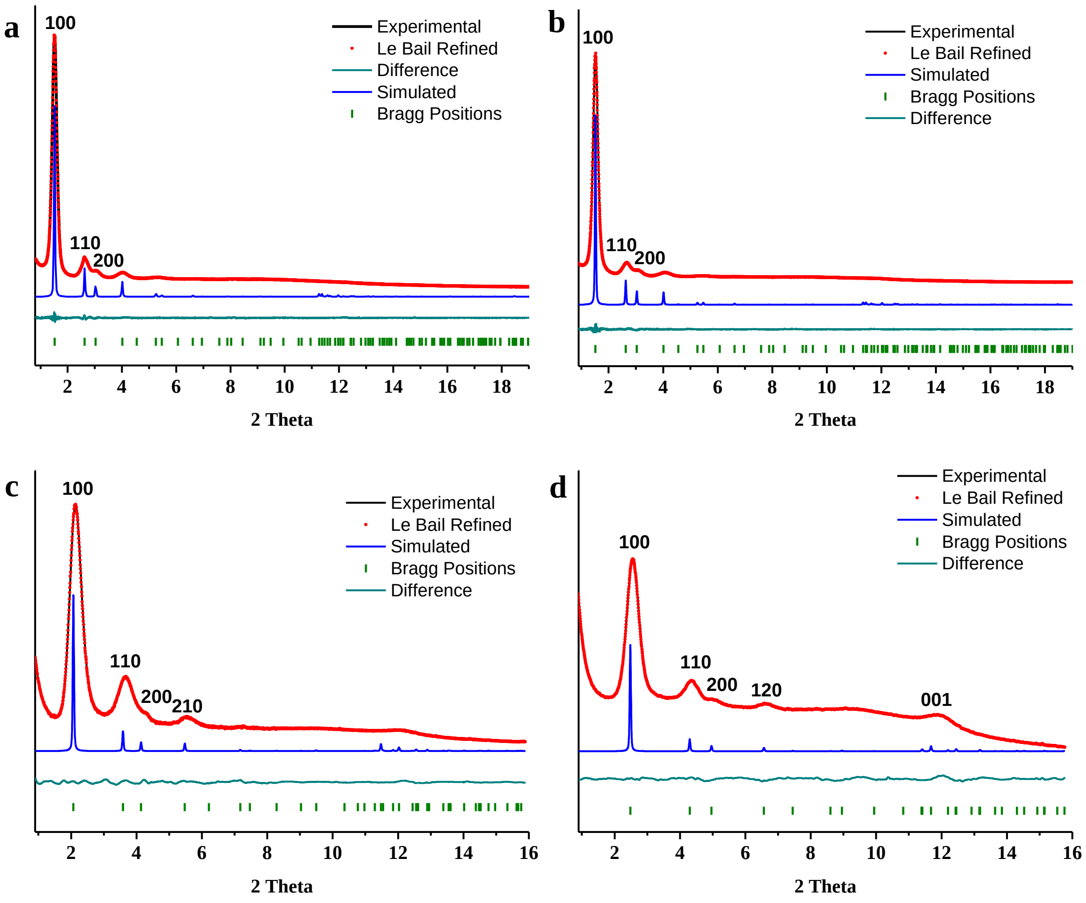

Supplementary Figure 18. Synchrotron PXRD patterns and Le Bail refinement results of chiral COFs.

Supplementary Table 3. The unit cell parameters via Le Bail refinement.

Chiral TpBD	Space group : *P*6	a = b = 29.78 Å, c = 3.50 Å<br>α = 90, β =90, γ = 120	R_p = 0.574 %; R_{wp} = 0.773 %; R_{exp} = 0.175 %
Chiral TpBD-Me_2	Space group : *P*6	a = b = 29.55 Å, c = 3.47 Å<br>α = 90, β =90, γ = 120	R_p = 0.480 %; R_{wp} = 0.660 %; R_{exp} = 0.280 %
Chiral TpPa-1	Space group : *P*3	a = b = 22.02 Å, c = 3.50 Å<br>α = 90, β =90, γ = 120	R_p = 0.418 %; R_{wp} = 0.559 %; R_{exp} = 0.185 %
Chiral TpTab	Space group : *P*3	a = b = 18.37 Å, c = 3.47 Å<br>α = 90, β =90, γ = 120	R_p = 0.546 % ; R_{wp} = 0.687 %; R_{exp} = 0.204 %

Supplementary Table 4.Atomistic coordinates for the refined unit cell parameters for (Λ)-TpBD via Rietveld refinement.

(Λ)-TpBD			
**N1**	0.5812(17)	1.1873(30)	0.5469(105)
**C2**	0.6308(9)	1.2249(10)	0.5644(11)
**C3**	0.5608(29)	1.1361(7)	0.5109(96)
**C4**	0.5083(32)	1.1056(5)	0.6044(71)
**C5**	0.4845(19)	1.0521(4)	0.5895(104)
**C6**	0.5119(1)	1.0272(10)	0.4800(29)
**C7**	0.5641(2)	1.0584(22)	0.3756(194)
**C8**	0.5884(11)	1.1120(21)	0.3888(228)
**C9**	0.6467(4)	1.2783(1)	0.5882(1)
**O10**	0.2665(6)	0.7047(7)	0.6021(11)
**C11**	0.2974(3)	0.6868(4)	0.5944(1)
**H12**	0.5563(3)	1.2031(52)	0.5695(121)
**H13**	0.6615(26)	1.2150(13)	0.5556(18)
**H14**	0.4868(46)	1.1244(16)	0.6998(147)
**H15**	0.4440(23)	1.0290(13)	0.6773(233)
**H16**	0.5858(17)	1.0403(33)	0.2726(297)
**H17**	0.6286(7)	1.1350(31)	0.2976(357)

Supplementary Table 5.Atomistic coordinates for the refined unit cell parameters for (*Λ*)-TpBD-Me$_2$ via Rietveld refinement.

(*Λ*)-TpBD-Me$_2$			
**N1**	0.5942(9)	1.1867(37)	0.5667(6)
**C2**	0.6426(3)	1.2273(14)	0.5725(13)
**C3**	0.5744(32)	1.1355(13)	0.5737(29)
**C4**	0.5225(42)	1.1116(11)	0.6976(163)
**C5**	0.4927(26)	1.0571(2)	0.7083(152)
**C6**	0.5149(4)	1.0272(11)	0.5932(53)
**C7**	0.5668(4)	1.0525(25)	0.4701(242)
**C8**	0.5973(3)	1.1065(26)	0.4550(238)
**C9**	0.6523(13)	1.2796(4)	0.5674(1)
**O10**	0.2584(19)	0.6952(25)	0.5654(5)
**C11**	0.2931(10)	0.6813(14)	0.5667(1)
**H12**	0.5670(13)	1.2010(63)	0.5657(43)
**H13**	0.6767(15)	1.2226(15)	0.5874(50)
**H14**	0.5063(60)	1.1358(21)	0.7907(320)
**H15**	0.4527(33)	1.0382(12)	0.8146(301)
**H16**	0.5833(22)	1.0289(35)	0.3647(398)
**C17**	0.6517(8)	1.1296(41)	0.2986(448)
**H18**	0.5006(26)	0.3385(11)	0.0998(403)
**H19**	0.4437(17)	0.3441(30)	0.1419(562)
**H20**	0.4632(58)	0.3182(8)	0.5250(550)

Supplementary Table 6.Atomistic coordinates for the refined unit cell parameters for (*Λ*)-TpPa-1 via Rietveld refinement.

(*Λ*)-TpPa-1			
**N1**	0.4023(20)	0.8620(39)	0.7560(389)
**C2**	0.4214(22)	0.8130(4)	0.8036(538)
**C3**	0.3774(11)	0.7417(2)	0.8578(538)
**O4**	0.7500(28)	0.2737(31)	0.1255(880)
**C5**	0.7114(15)	0.3016(17)	0.1293(800)
**C6**	0.5096(3)	0.0721(10)	0.3956(181)
**C7**	0.5470(2)	0.0370(17)	0.4594(108)
**C8**	0.5127(9)	0.9679(27)	0.5800(79)
**H9**	0.3472(21)	0.8358(40)	0.7954(477)
**H10**	0.4774(20)	0.8322(14)	0.7752(538)
**H11**	0.6035(1)	0.0621(1)	0.4350(1)
**H12**	0.5431(13)	0.9419(32)	0.6285(131)
**N13**	0.5413(11)	0.1419(1)	0.2777(368)
**C14**	0.6078(32)	0.1870(4)	0.1937(880)
**C15**	0.6354(16)	0.2583(2)	0.1380(88)
**O16**	0.2743(25)	0.7505(22)	0.8683(538)
**C17**	0.3019(13)	0.7117(12)	0.8657(538)
**C18**	0.4400(12)	0.9319(29)	0.6393(198)
**C19**	0.4027(7)	0.9671(22)	0.5761(126)
**C20**	0.4370(1)	0.0362(13)	0.4551(61)
**H21**	0.5120(14)	0.1678(4)	0.2398(421)
**H22**	0.6445(43)	0.1676(17)	0.2232(88)
**H23**	0.3462(9)	0.9394(25)	0.6218(218)
**H24**	0.4081(10)	0.0625(10)	0.4175(10)

Supplementary Table 7.Atomistic coordinates for the refined unit cell parameters for (*Λ*)-TpTab via Rietveld refinement.

(*Λ*)-TpTab			
**O1**	0.5051(3)	1.7610(35)	0.7438(1)
**N2**	0.4889(7)	1.8973(34)	0.5882(421)
**C3**	0.4253(2)	1.7174(19)	0.7382(1)
**C4**	0.3737(20)	1.7564(2)	0.7291(1)
**C5**	0.4091(26)	1.8445(28)	0.6863(82)
**C6**	0.5243(4)	1.9799(9)	0.5107(227)
**C7**	0.6093(33)	2.0329(3)	0.5954(241)
**C8**	0.4773(36)	2.0139(28)	0.3565(424)
**C9**	0.6332(3)	2.2458(1)	0.3930(382)
**C10**	0.7199(3)	2.3004(3)	0.3919(38)
**C11**	0.5977(6)	2.1544(18)	0.4144(217)
**C12**	0.5139(41)	2.0996(32)	0.3097(243)
**C13**	0.6448(29)	2.1188(1)	0.5506(456)
**H14**	0.5241(21)	1.8661(43)	0.5772(82)
**H15**	0.3660(20)	1.8685(19)	0.7227(537)
**H16**	0.6459(64)	2.0063(17)	0.7142(426)
**H17**	0.4125(65)	1.9731(40)	0.2663(82)
**H18**	0.7616(5)	2.2744(6)	0.3991(382)
**H19**	0.4768(75)	2.1244(49)	0.1799(431)
**H20**	0.7098(58)	2.1592(11)	0.6391(81)

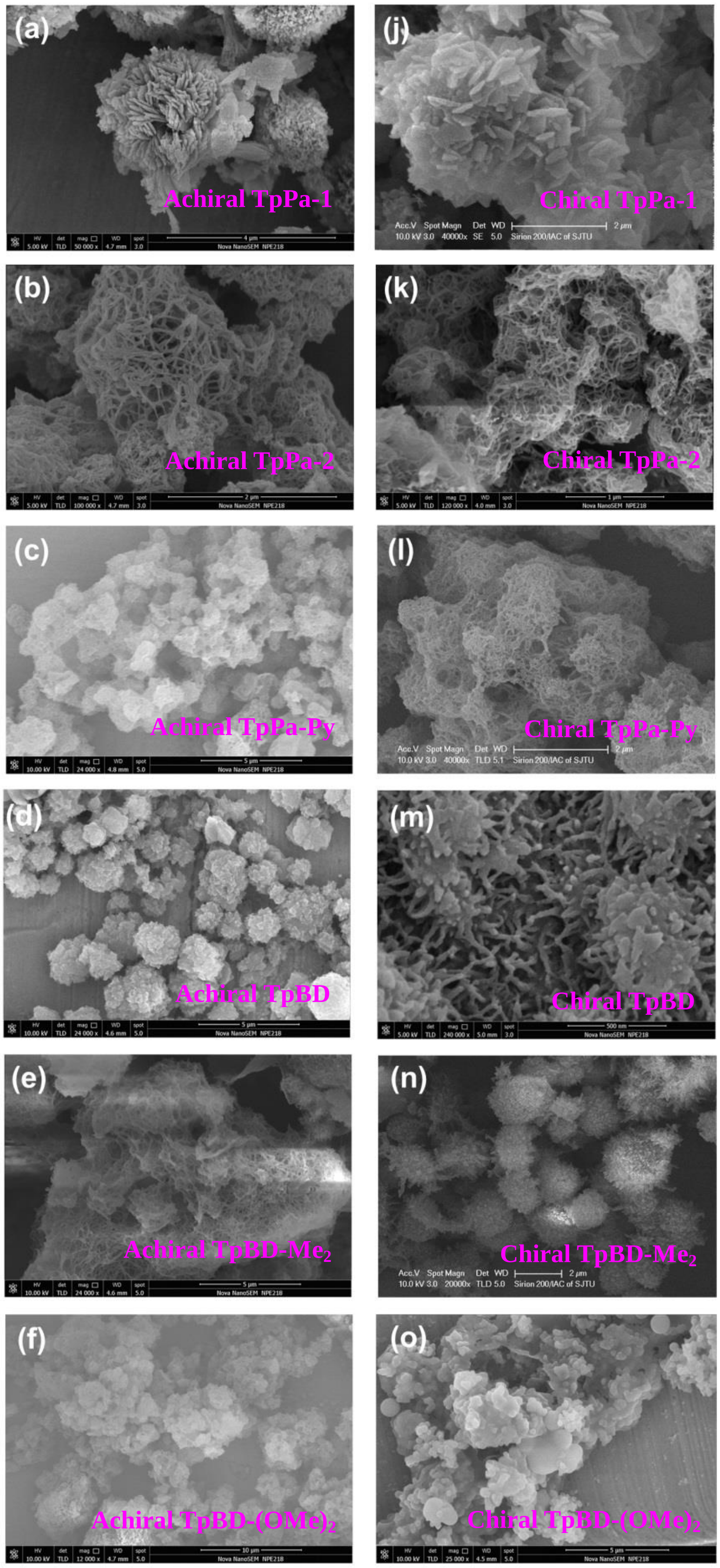
(a)
Achiral TpPa-1
(j)
Chiral TpPa-1
(b)
Achiral TpPa-2
(k)
Chiral TpPa-2
(c)
Achiral TpPa-Py
(l)
Chiral TpPa-Py
(d)
Achiral TpBD
(m)
Chiral TpBD
(e)
Achiral TpBD-Me2
(n)
Chiral TpBD-Me2
(f)
Achiral TpBD-(OMe)2
(o)
Chiral TpBD-(OMe)2

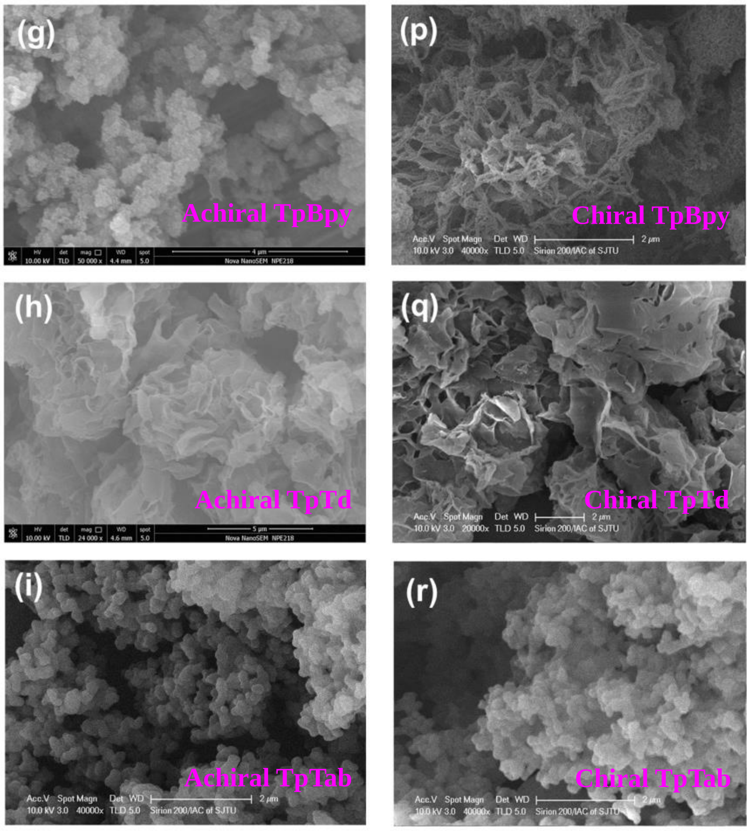

Supplementary Figure 19. The SEM images. a-i, Achiral TpPa-1, TpPa-2, TpPa-Py, TpBD, TpBD-Me_2, TpBD-$(OMe)_2$, TpBpy ,TpTd and TpTab, respectively. **j-r**, Chiral TpPa-1, TpPa-2, TpPa-Py, TpBD, TpBD-Me_2, TpBD-$(OMe)_2$, TpBpy ,TpTd and TpTab, respectively.

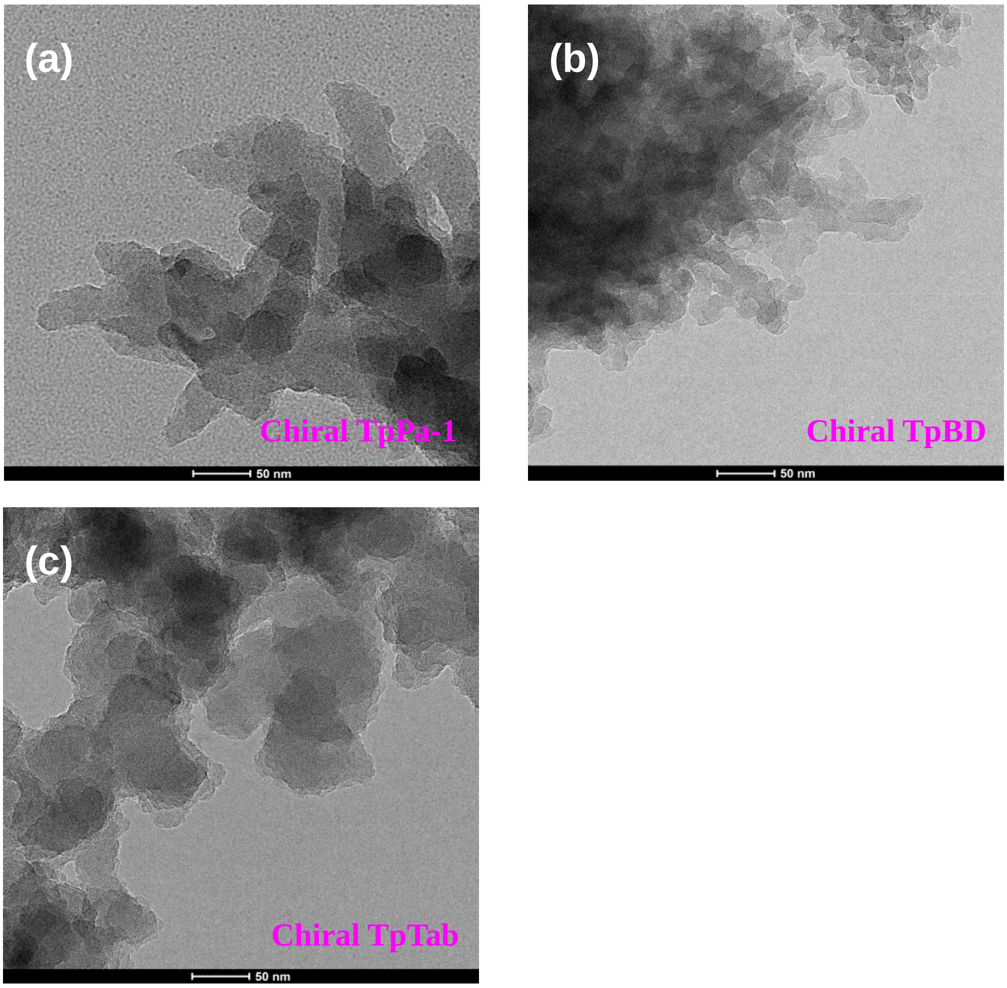

Supplementary Figure 20. The TEM images. a-c, Chiral TpPa-1, TpBD, and TpTab, respectively.

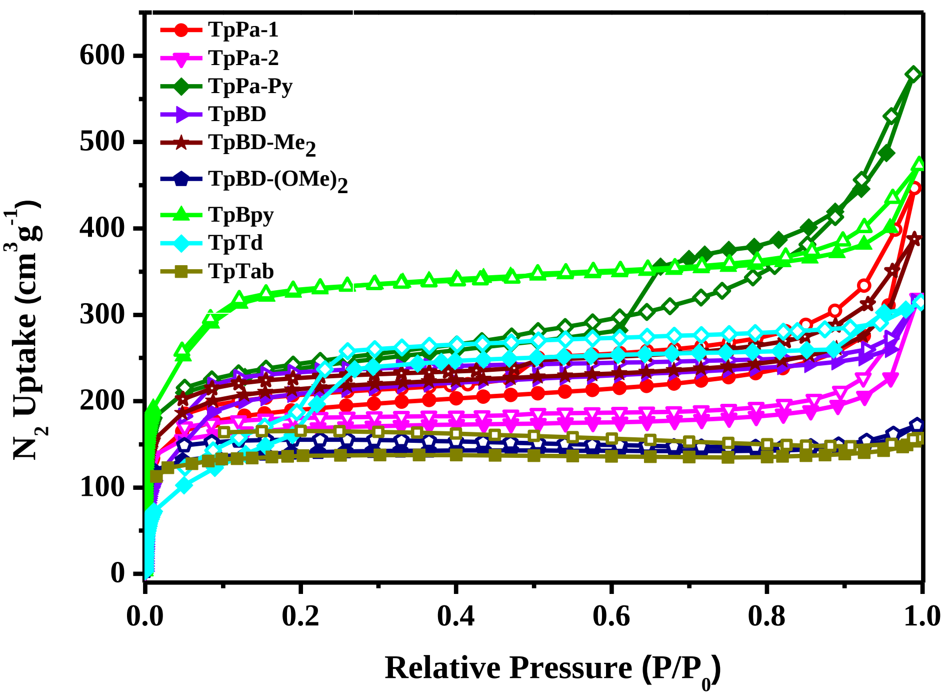

Supplementary Figure 21. N_2 adsorption isotherms of achiral COFs.

Supplementary Table 8. Surface areas of achiral and chiral COFs.

A comparison BET surface areas of achiral and chiral COFs		
	Achiral COFs (cm^3 g^{-1})	Chiral COFs (cm^3 g^{-1})
**TpPa-1**	533.3	832.4
**TpPa-2**	520	1077.4
**TpPa-Py**	759	1178
**TpBD**	674.4	849.3
**TpBD-Me_2**	671.2	878.1
**TpBD-$(OMe)_2$**	431.8	602.8
**TpBpy**	1040.3	1073.4
**TpTd**	857.2	1217.8
**TpTab**	461.4	482.6

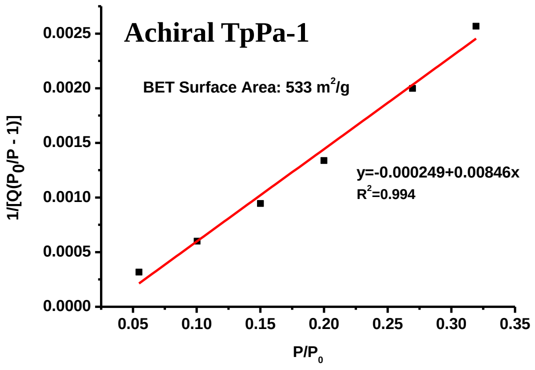
Achiral TpPa-1
BET Surface Area: 533 m2/g
y=-0.000249+0.00846x
R2=0.994
1/[Q(P0/P - 1)]
P/P0

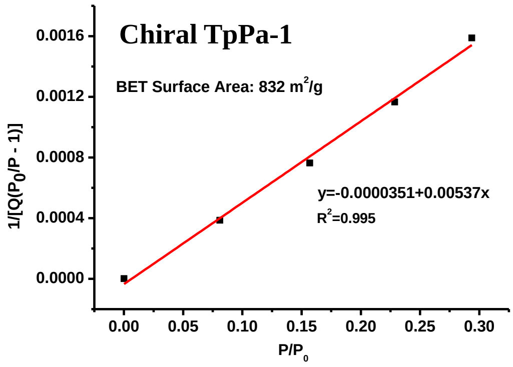
Chiral TpPa-1
BET Surface Area: 832 m2/g
y=-0.0000351+0.00537x
R2=0.995
1/[Q(P0/P - 1)]
P/P0

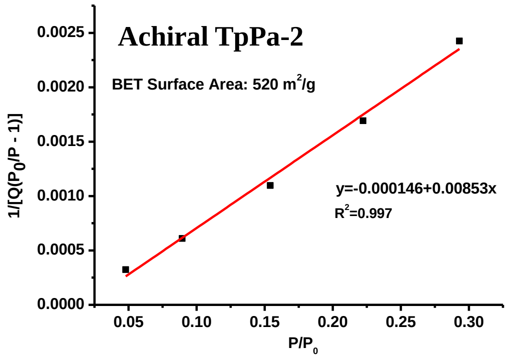
Achiral TpPa-2
BET Surface Area: 520 m2/g
y=-0.000146+0.00853x
R2=0.997
1/[Q(P0/P - 1)]
P/P0

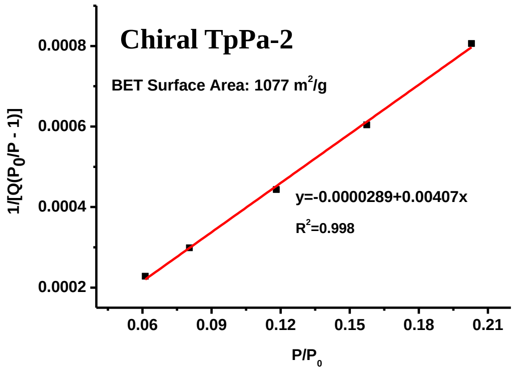
Chiral TpPa-2
BET Surface Area: 1077 m2/g
y=-0.0000289+0.00407x
R2=0.998
1/[Q(P0/P - 1)]
P/P0

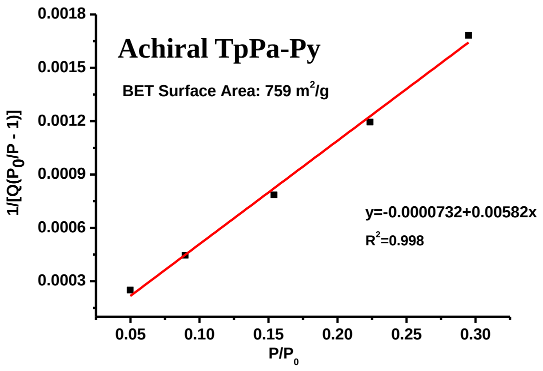
Achiral TpPa-Py
BET Surface Area: 759 m2/g
y=-0.0000732+0.00582x
R2=0.998
1/[Q(P0/P - 1)]
P/P0

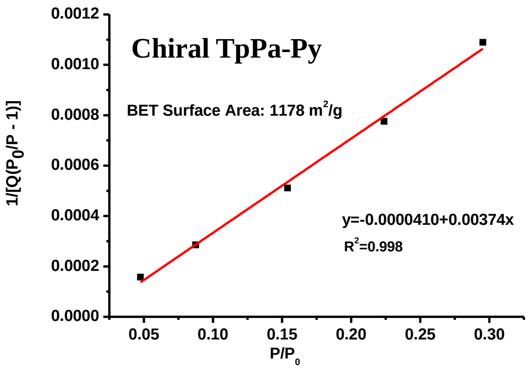
Chiral TpPa-Py
BET Surface Area: 1178 m2/g
y=-0.0000410+0.00374x
R2=0.998
1/[Q(P0/P - 1)]
P/P0

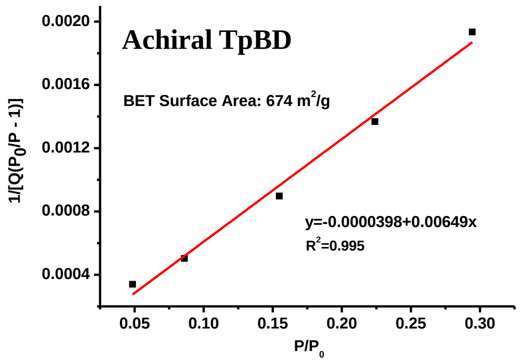
Achiral TpBD
BET Surface Area: 674 m2/g
y=-0.0000398+0.00649x
R2=0.995
1/[Q(P0/P - 1)]
P/P0

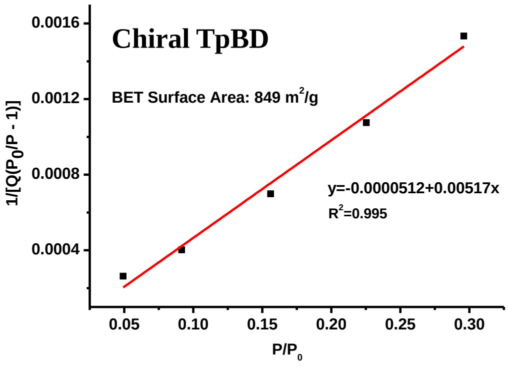
Chiral TpBD
BET Surface Area: 849 m2/g
y=-0.0000512+0.00517x
R2=0.995
1/[Q(P0/P - 1)]
P/P0

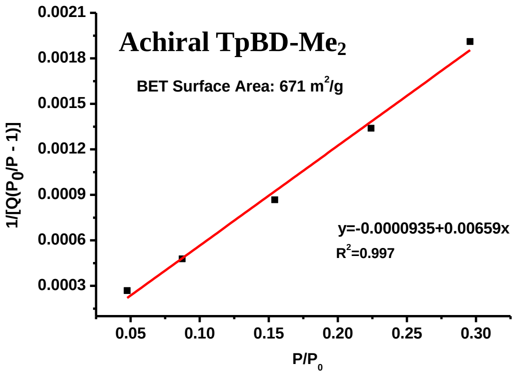
Achiral TpBD-Me2
BET Surface Area: 671 m2/g
y=-0.0000935+0.00659x
R2=0.997
1/[Q(P0/P - 1)]
P/P0

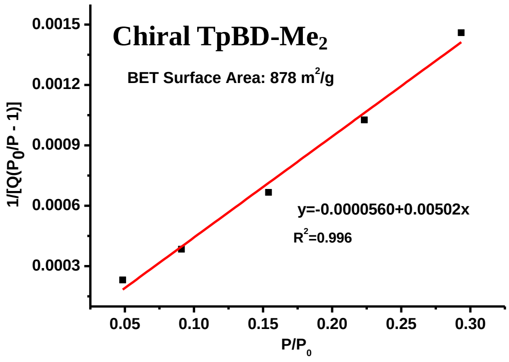
Chiral TpBD-Me2
BET Surface Area: 878 m2/g
y=-0.0000560+0.00502x
R2=0.996
1/[Q(P0/P - 1)]
P/P0

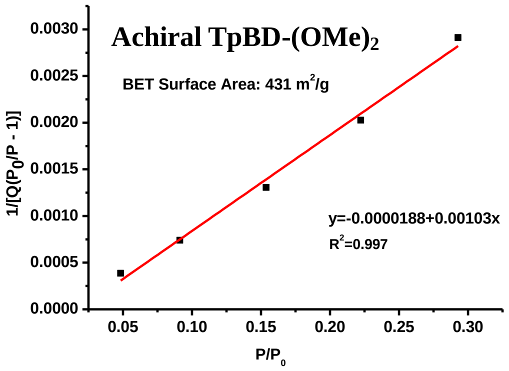
Achiral TpBD-(OMe)2
BET Surface Area: 431 m2/g
y=-0.0000188+0.00103x
R2=0.997
1/[Q(P0/P - 1)]
P/P0

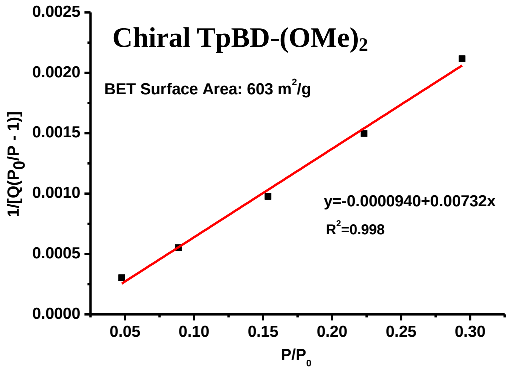
Chiral TpBD-(OMe)2
BET Surface Area: 603 m2/g
y=-0.0000940+0.00732x
R2=0.998
1/[Q(P0/P - 1)]
P/P0

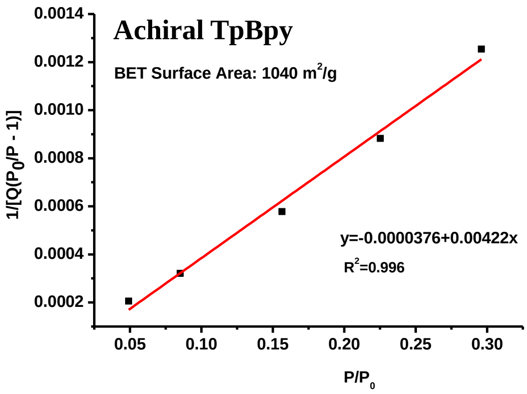
Achiral TpBpy
BET Surface Area: 1040 m2/g
y=-0.0000376+0.00422x
R2=0.996
1/[Q(P0/P - 1)]
P/P0

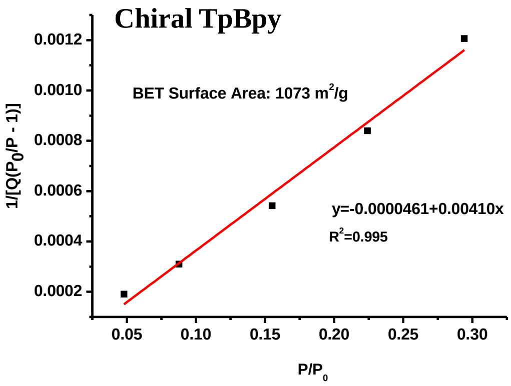
Chiral TpBpy
BET Surface Area: 1073 m2/g
y=-0.0000461+0.00410x
R2=0.995
1/[Q(P0/P - 1)]
P/P0

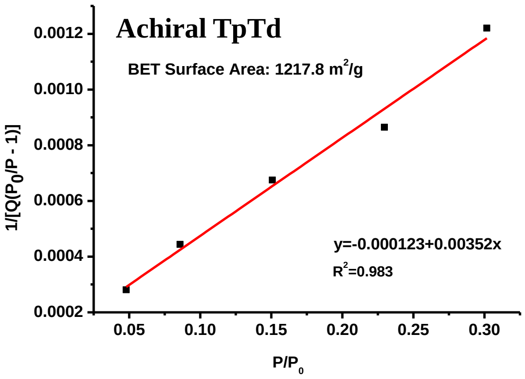
Achiral TpTd
BET Surface Area: 1217.8 m2/g
y=-0.000123+0.00352x
R2=0.983
1/[Q(P0/P - 1)]
P/P0

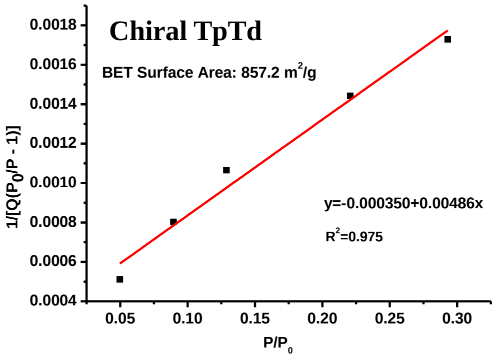
Chiral TpTd
BET Surface Area: 857.2 m2/g
y=-0.000350+0.00486x
R2=0.975
1/[Q(P0/P - 1)]
P/P0

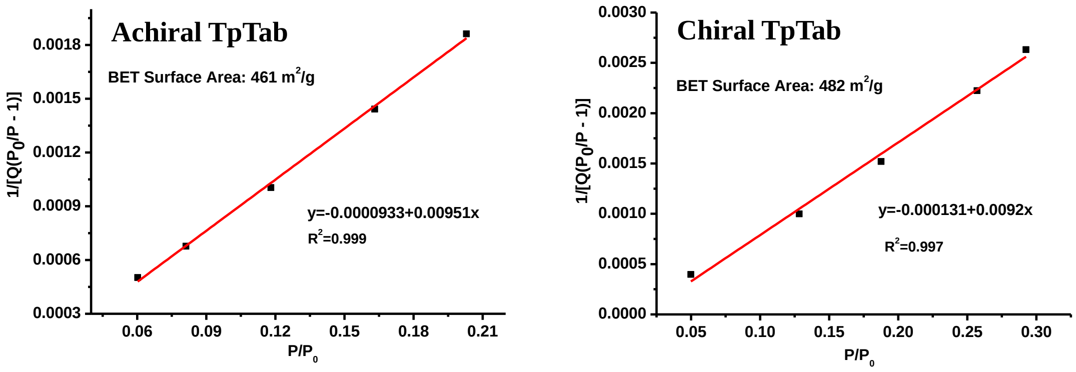

Supplementary Figure 22. BET plots of achiral and chiral COFs.

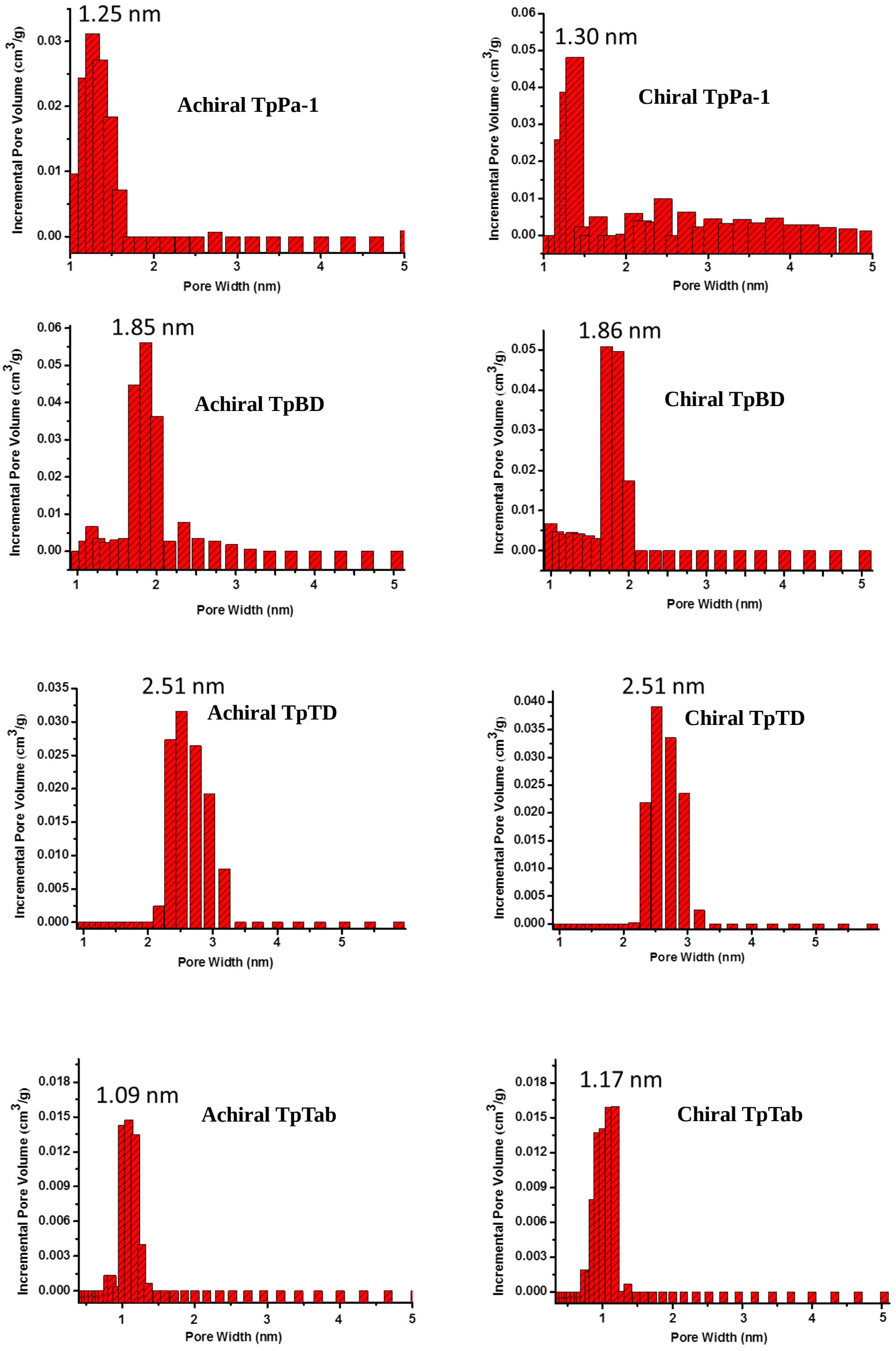

Supplementary Figure 23. The pore size distributions of achiral and chiral COFs.

Supplementary Table 9. SV constants of (*Δ*)- or (*Λ*)-TpTab with different chiral carbohydrates quenchers.

D-glucose	**$K_{SV}(\Delta)$,** M^{-1}	5323 ±500	D-cellobiose	**$K_{SV}(\Delta)$,** M^{-1}	3806 ±200
	**$K_{SV}(\Lambda)$,** M^{-1}	8302 ±800		**$K_{SV}(\Lambda)$,** M^{-1}	13086 ±1000
	**QR[a]**	1.56 ±0.2		**QR[a]**	3.44 ±0.26
D-mannitol	**$K_{SV}(\Delta)$,** M^{-1}	4333 ±400	D-sucrose	**$K_{SV}(\Delta)$,** M^{-1}	5373 ±340
	**$K_{SV}(\Lambda)$,** M^{-1}	9427 ±1200		**$K_{SV}(\Lambda)$,** M^{-1}	13102 ±600
	**QR[a]**	2.18 ±0.4		**QR[a]**	2.43 ±0.16
D-lactose	**$K_{SV}(\Delta)$,** M^{-1}	5435 ±400	D-maltose	**$K_{SV}(\Delta)$,** M^{-1}	3952.1 ±100
	**$K_{SV}(\Lambda)$,** M^{-1}	10599 ±1400		**$K_{SV}(\Lambda)$,** M^{-1}	14293 ±253
	**QR[a]**	1.59 ±0.25		**QR[a]**	3.62 ±0.16
D-sorbitol	**$K_{SV}(\Delta)$,** M^{-1}	4856 ±300	*D*-lactobionic acid	**$K_{SV}(\Delta)$,** M^{-1}	2429 ±460
	**$K_{SV}(\Lambda)$,** M^{-1}	10913 ±440		**$K_{SV}(\Lambda)$,** M^{-1}	4851 ±300
	**QR[a]**	2.24 ±0.19		**QR[a]**	2.00 ±0.30
D-fructose	**$K_{SV}(\Delta)$,** M^{-1}	4218 ±100	D-glucuronic acid	**$K_{SV}(\Delta)$,** M^{-1}	7347 ±1000
	**$K_{SV}(\Lambda)$,** M^{-1}	10514 ±550		**$K_{SV}(\Lambda)$,** M^{-1}	9690 ±1300
	**QR[a]**	2.49 ±0.21		**QR[a]**	1.32 ±0.41
D-gentiobiose	**$K_{SV}(\Delta)$,** M^{-1}	3579 ±300	D-gluconic acid	**$K_{SV}(\Delta)$,** M^{-1}	5239 ±300
	**$K_{SV}(\Lambda)$,** M^{-1}	9549 ±440		**$K_{SV}(\Lambda)$,** M^{-1}	10681 ±610
	**QR[a]**	2.66 ±0.10		**QR[a]**	2.03 ±0.28

[a]QR=$K_{SV}(\Lambda)/K_{SV}(\Delta)$

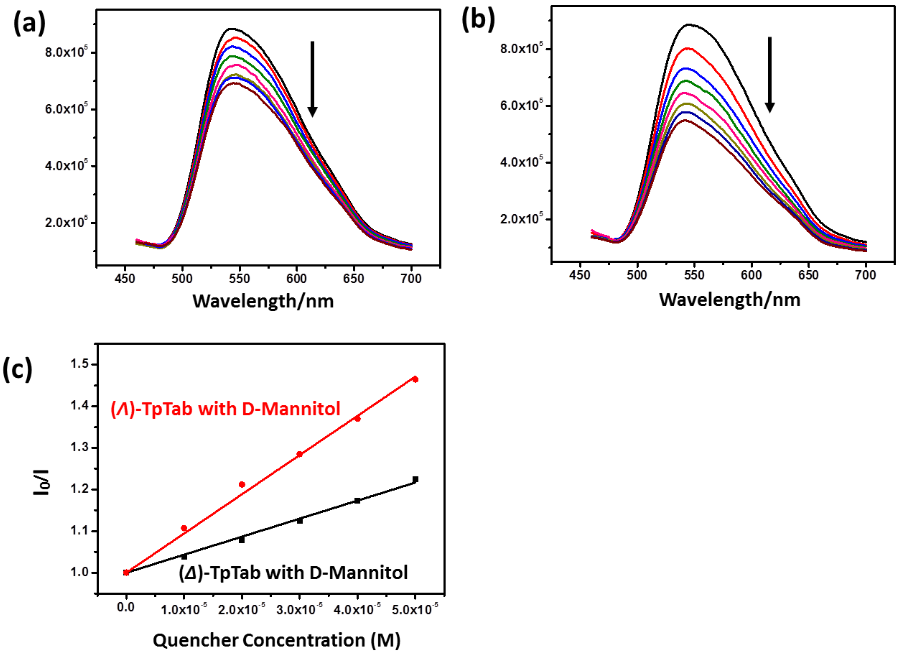

Supplementary Figure 24. (a) and (b) Fluorescence emission spectra of (*Δ*)- and (*Λ*)-TpTab with increasing concentration of D-mannitol (0, 1, 2, 3, 4, 5, 6 and 7×10^{-5} M from top to bottom). (c) The corresponding SV plots.

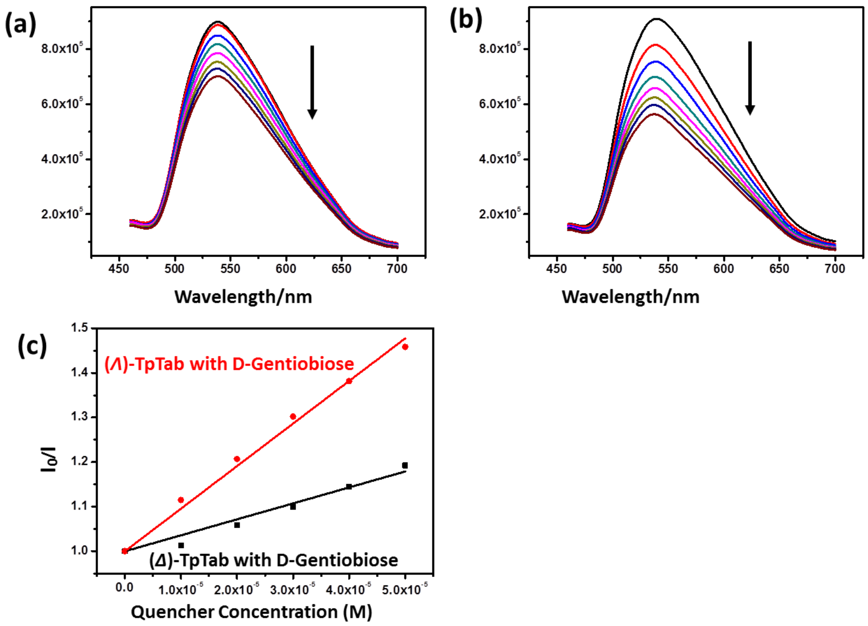

Supplementary Figure 25. (a) and (b) Fluorescence emission spectra of (*Δ*)- and (*Λ*)-TpTab with increasing concentration of D-gentiobiose (0, 1, 2, 3, 4, 5, 6 and 7×10^{-5} M from top to bottom). (c) The corresponding SV plots.

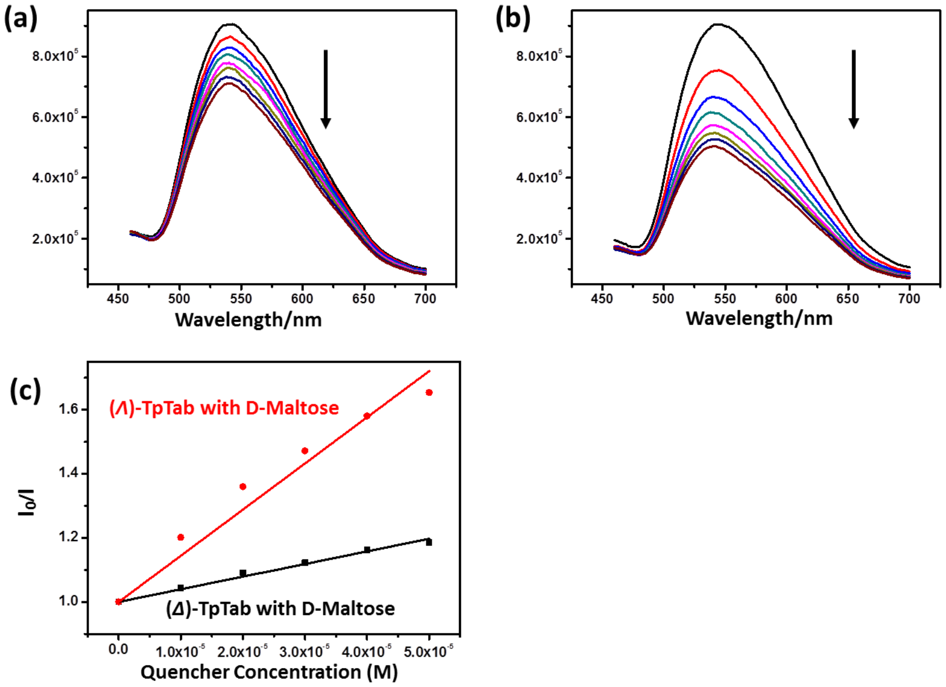

Supplementary Figure 26. (a) and (b) Fluorescence emission spectra of (*Δ*)- and (*Λ*)-TpTab with increasing concentration of D-maltose (0, 1, 2, 3, 4, 5, 6 and 7 $\times 10^{-5}$ M from top to bottom). (c) The corresponding SV plots.

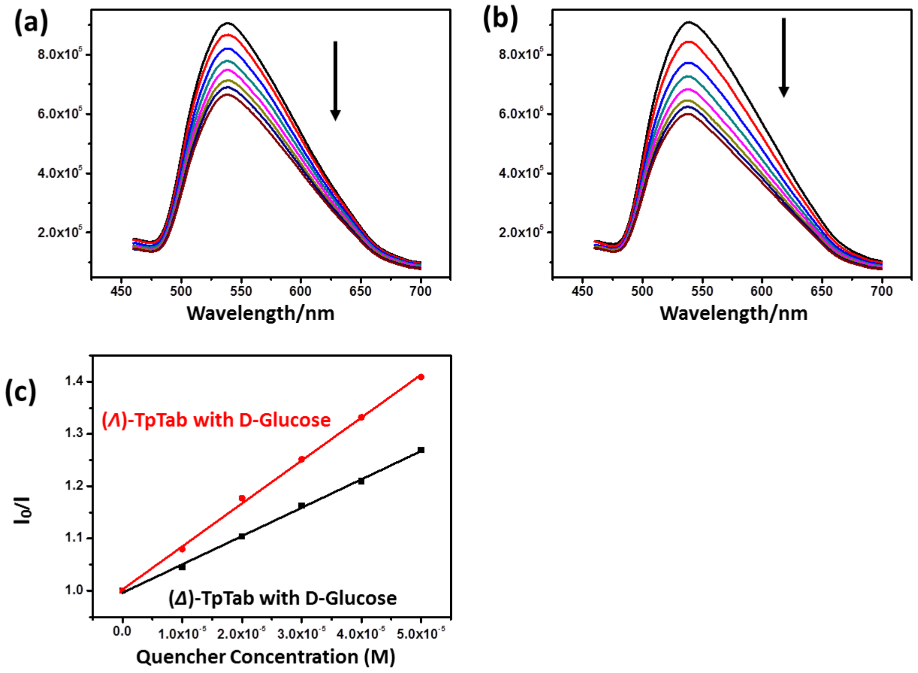

Supplementary Figure 27. (a) and (b) Fluorescence emission spectra of (*Δ*)- and (*Λ*)-TpTab with increasing concentration of D-glucose (0, 1, 2, 3, 4, 5, 6 and 7×10^{-5} M from top to bottom). (c) The corresponding SV plots.

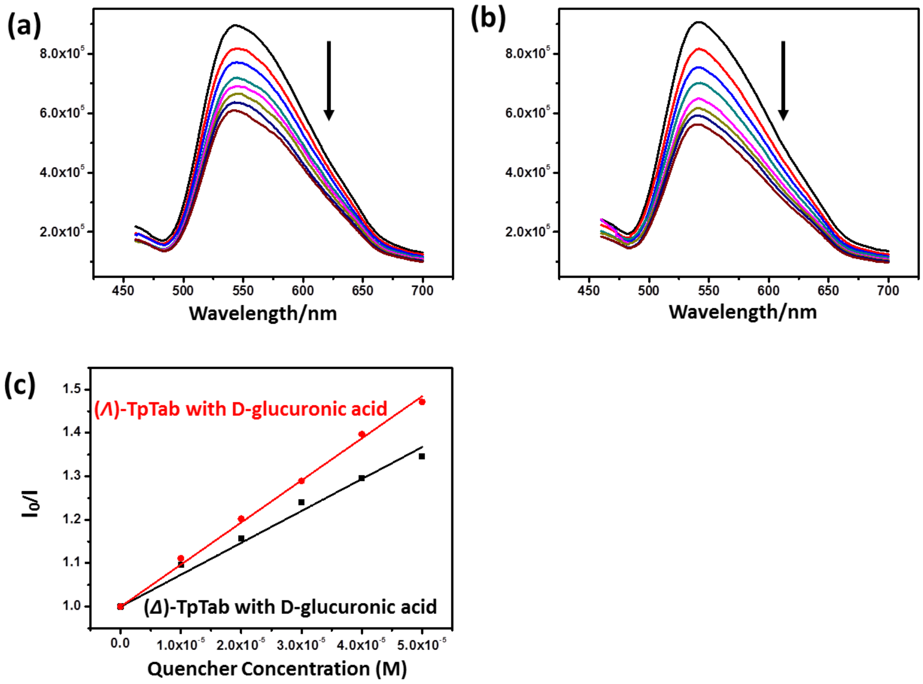

Supplementary Figure 28. (a) and (b) Fluorescence emission spectra of (*Δ*)- and (*Λ*)-TpTab with increasing concentration of D-glucuronic acid (0, 1, 2, 3, 4, 5, 6 and 7×10^{-5} M from top to bottom). (c) The corresponding SV plots.

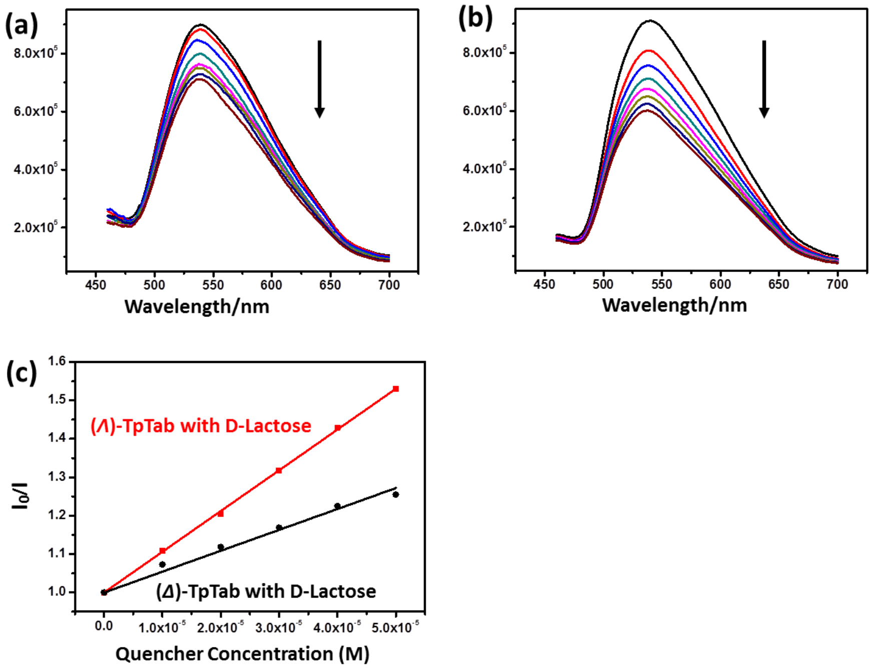

Supplementary Figure 29. (a) and (b) Fluorescence emission spectra of (*Δ*)- and (*Λ*)-TpTab with increasing concentration of D-lactose (0, 1, 2, 3, 4, 5, 6 and 7×10^{-5} M from top to bottom). (c) The corresponding SV plots.

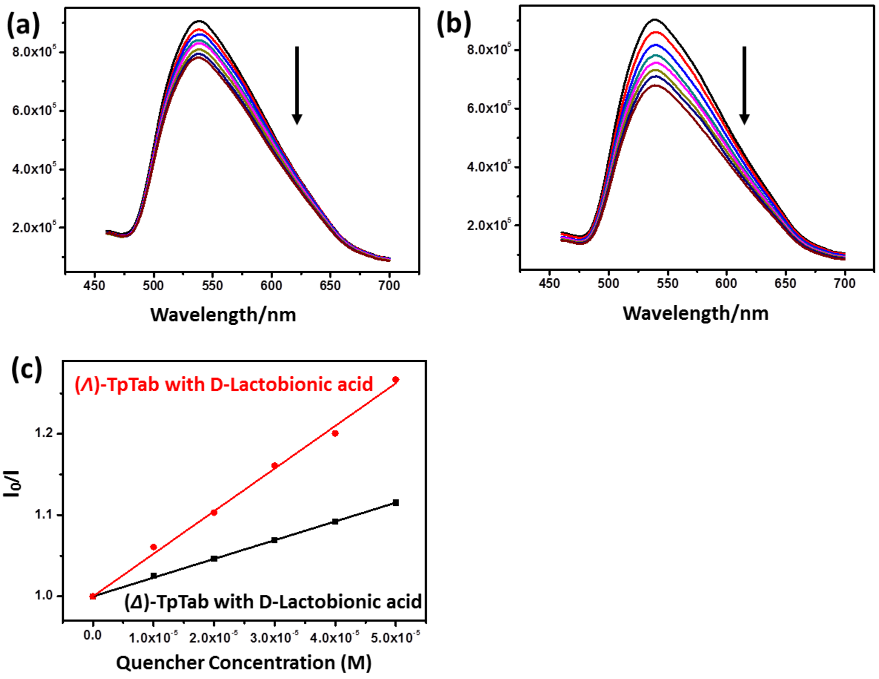

Supplementary Figure 30. (a) and (b) Fluorescence emission spectra of (*Δ*)- and (*Λ*)-TpTab with increasing concentration of D-lactobionic acid (0, 1, 2, 3, 4, 5, 6 and 7×10^{-5} M from top to bottom). (c) The corresponding SV plots.

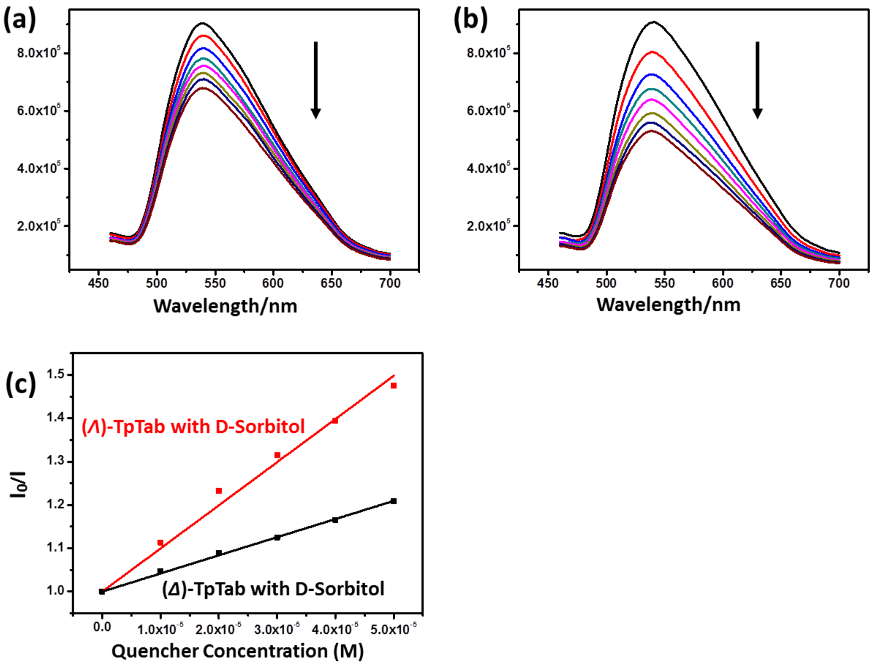

Supplementary Figure 31. (a) and (b) Fluorescence emission spectra of (*Δ*)- and (*Λ*)-TpTab with increasing concentration of D-Sobitol (0, 1, 2, 3, 4, 5, 6 and 7×10^{-5} M from top to bottom). (c) The corresponding SV plots.

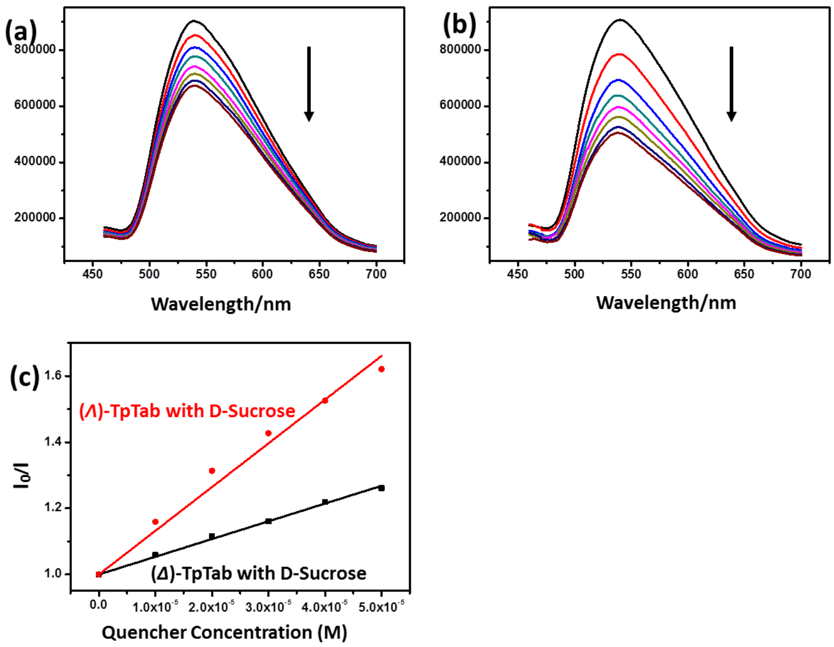

Supplementary Figure 32. (a) and (b) Fluorescence emission spectra of (*Δ*)- and (*Λ*)-TpTab with increasing concentration of D-sucrose (0, 1, 2, 3, 4, 5, 6 and 7×10^{-5} M from top to bottom). (c) The corresponding SV plots.

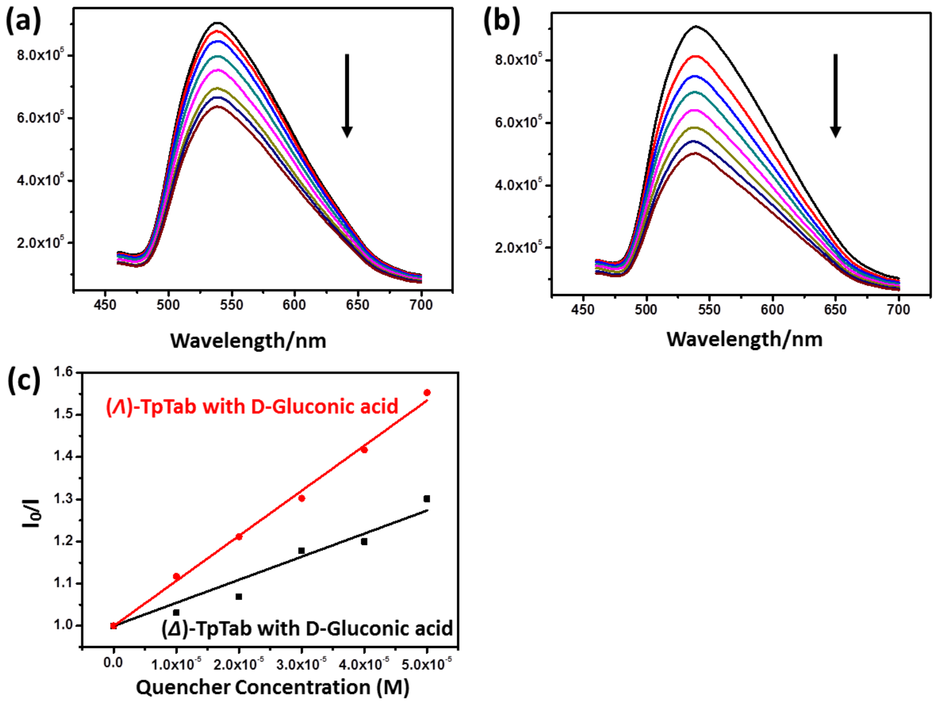

Supplementary Figure 33. (a) and (b) Fluorescence emission spectra of (*Δ*)- and (*Λ*)-TpTab with increasing concentration of D-gluconic acid (0, 1, 2, 3, 4, 5, 6 and 7×10^{-5} M from top to bottom). (c) The corresponding SV plots.

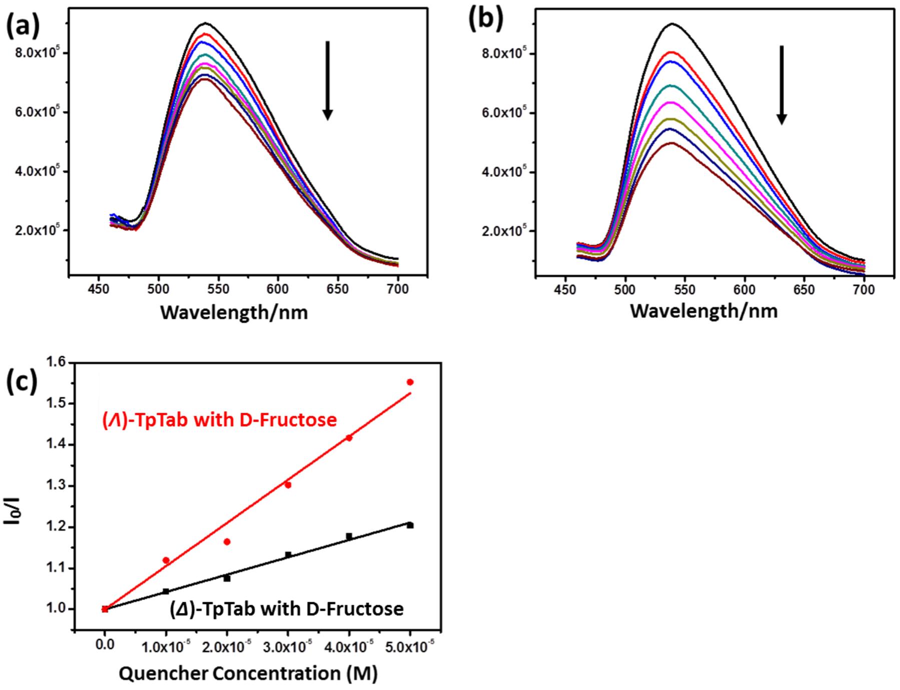

Supplementary Figure 34. (a) and (b) Fluorescence emission spectra of (*Δ*)- and (*Λ*)-TpTab with increasing concentration of D-fructose (0, 1, 2, 3, 4, 5, 6 and 7×10^{-5} M from top to bottom). (c) The corresponding SV plots.

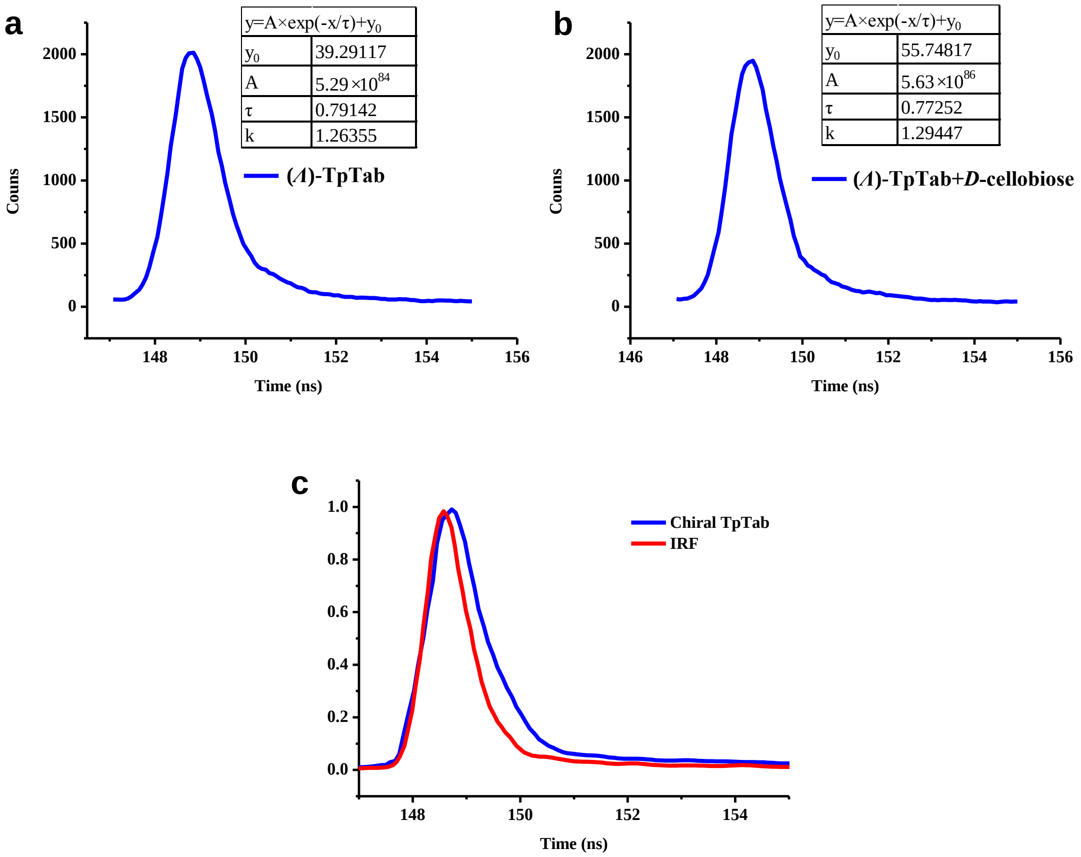

Supplementary Figure 35. Emission decay traces at λ = 540 nm of (*Λ*)-TpTab in the presence of the *D*-cellobiose quencher (The lifetime profile is one component). a, Emission decay of (*Λ*)-TpTab; **b**, (*Λ*)-TpTab in the presence of 0.07 mM of *D*-cellobiose; **c**, Instrument Response Function (IRF) was measured at the emission wavelength of λ = 387 nm under the same condition as other measurements.

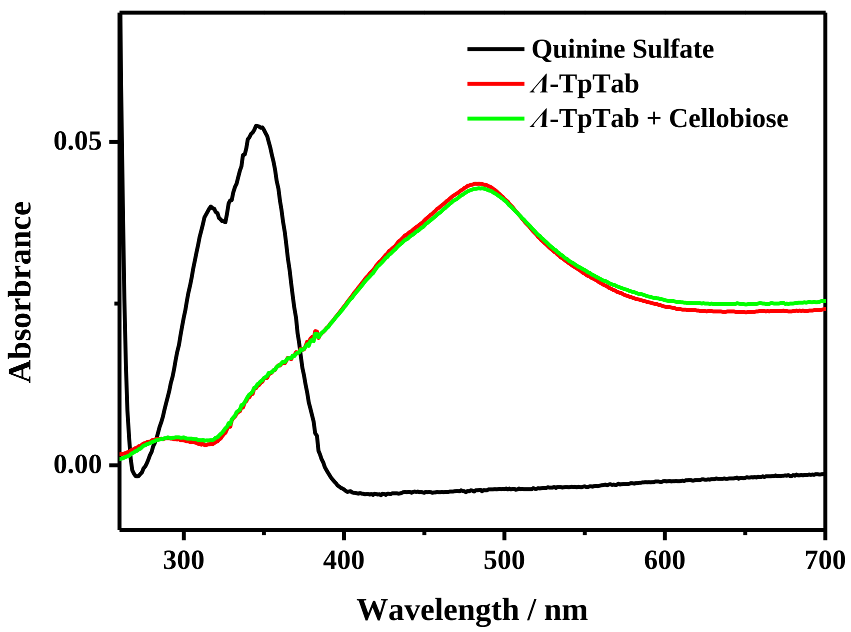

Supplementary Figure 36. UV/Vis absorption spectra of Quinine Sulfate, (*Λ*)-TpTab before and after addition *D*-cellobiose (1.0 $\times 10^{-3}$ mol/L).

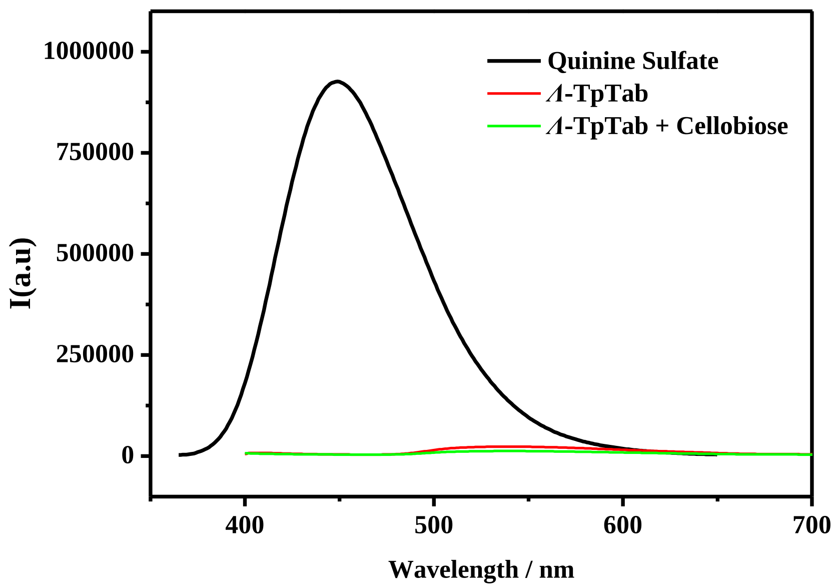

Supplementary Figure 37. Fluorescence spectra of Quinine Sulfate：(*Λ*)-TpTab before and after addition *D*-cellobiose (1.0×10^{-3} mol/L).

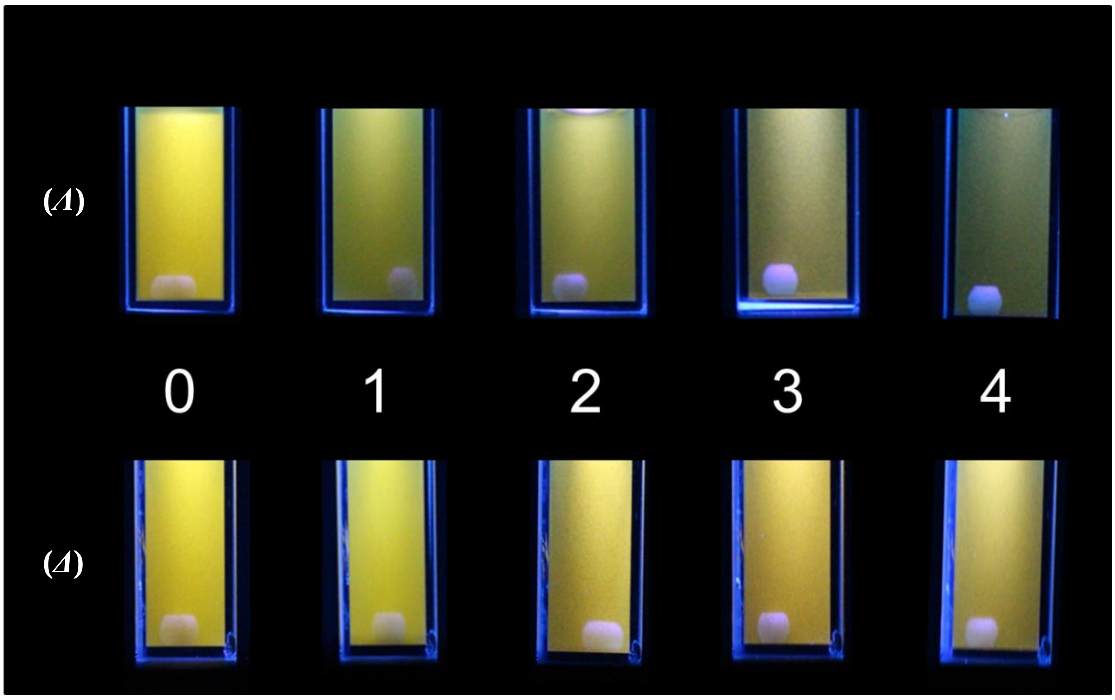

Supplementary Figure 38. The photos of Fluorescence quenching of (*Δ*)- or (*Λ*)-TpTab with increasing concentration of the *D*-cellobiose quencher in solution: 0, 1, 2, 3, 4 $\times 10^{-5}$ M under the 365 nm UV light.

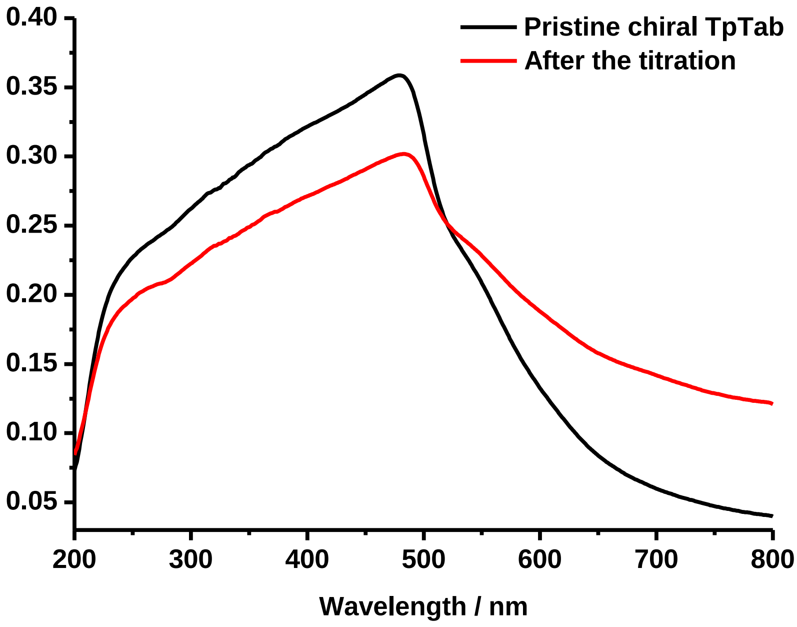

Supplementary Figure 39. The solid-state UV spectra of CCOF-TpTab before and after titration with *D*-cellobiose.

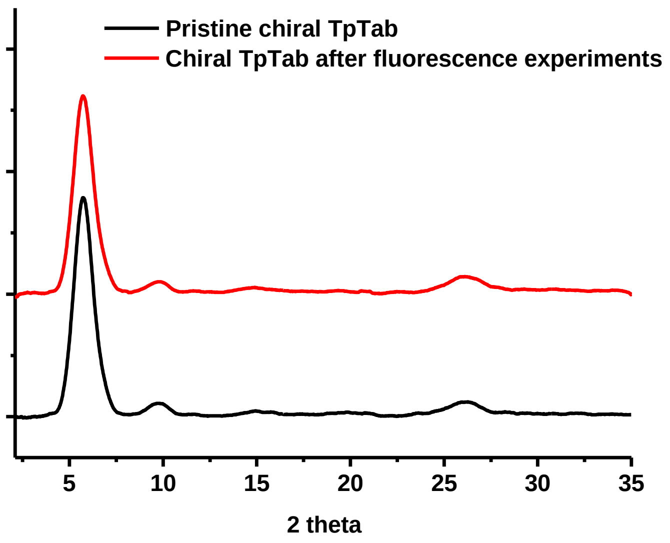

Supplementary Figure 40. PXRD of CCOF TpTab before and after fluorescence sensing experiments.

Supplementary Table 10. Elemental analysis of chiral TpTab after soaking in D-Cellobiose solution. Elemental analysis suggested the formation of the host–guest complex, which can be formulated as [TpTab cellobiose] (based on C:N ratios).

		Weight [mg]	N [%]	C [%]
Before	Run-1	5.528	7.78	73.35
	Run-2	5.203	7.79	73.42
	Average		7.79	73.39
After	Run-1	4.435	5.21	64.79
	Run-2	4.983	5.23	64.66
	Average		5.22	64.73

$$\mathbf{Molar\ ratio} = \frac{\mathbf{n(chiral\ TpTab)}}{\mathbf{n(D-Cellobiose)}} = \mathbf{1:0.9}$$

(*R*)-1-(4-bromophenyl)-2-nitroethanol.

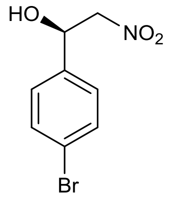

Trace conversion, but 34.5% ee by HPLC analysis (Chiralcel OD-H column, 1 mL/min, 15% *i*PrOH in hexane, 230 nm), (*R*) t_R=11.2 (major) and (*S*) t_R=14.9 (minor). ^{1}H NMR (400 MHz, $CDCl_3$) δ7.54–7.51(m, 2H), 7.33–7.27(m, 2H), 5.43 (dd, J = 9.3, 2.9 Hz, 1H), 4.57 (dd, J = 13.5, 9.3 Hz, 1H), 4.49 (dd, J = 13.5, 3.2 Hz, 1H), 3.07 (s, 1H).

a

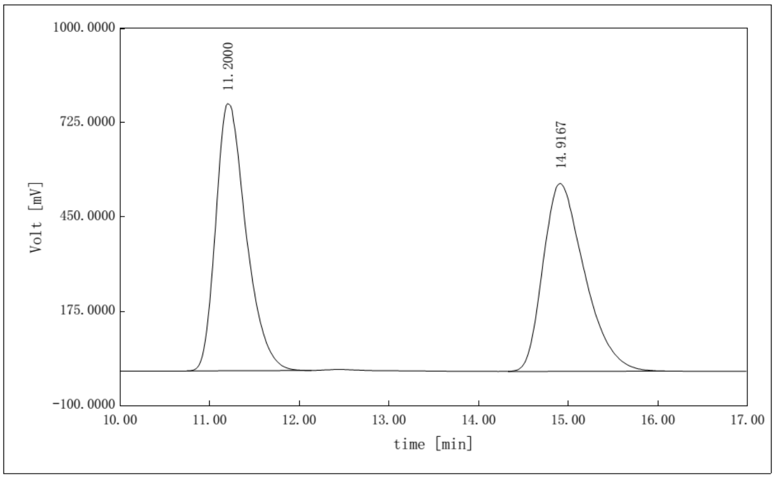

b

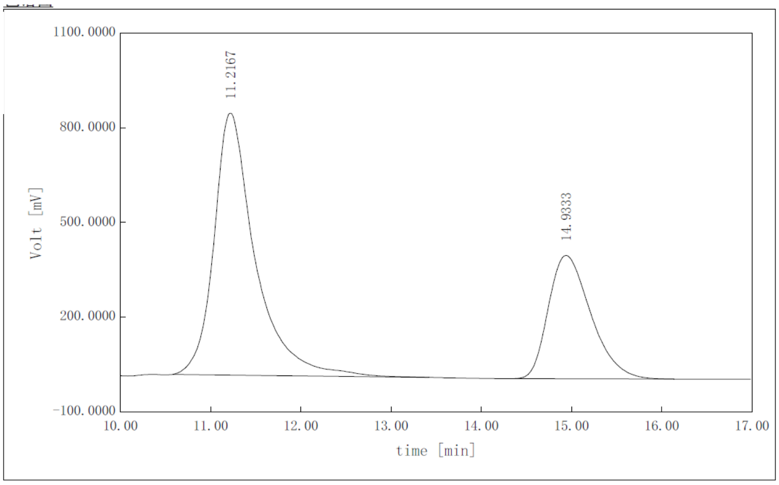

c

Serial Number	Retention Time[min]	Area[mAbs*s]	Type	Area%
1	11.2000	18348.8314	BB	50.9958
2	14.9127	17632.2558	BB	49.0042
The Total		35981.0872		

Supplementary Figure 41. HPLC spectra for (*R*)-1-(4-bromophenyl)-2-nitroethanol
a, Racemic standard. **b**, After reaction. **c**, The detail of integration.

a

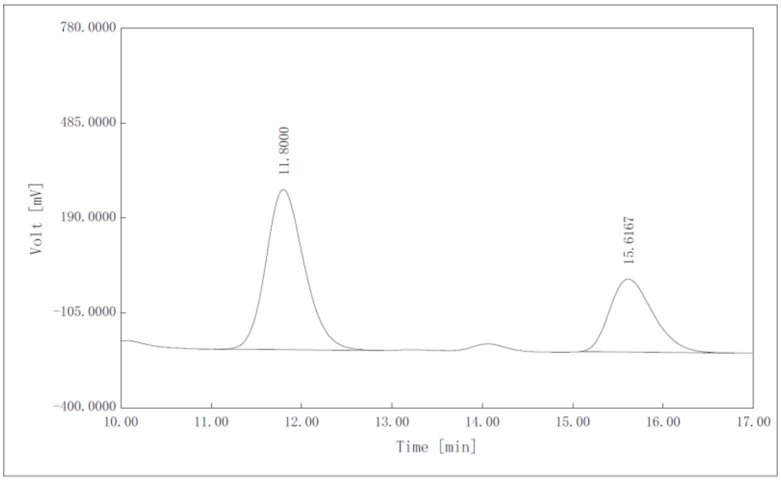

b

Serial Number	Retention Time[min]	Area[mAbs*s]	Type	Area%
1	11.8500	9060.5837	BB	67.9867
2	15.8667	4266.4037	BB	32.0133
The Total		13326.9874		

Supplementary Figure 42. HPLC spectra for the second run. a, After reaction. **b**, The detail of integration.

a

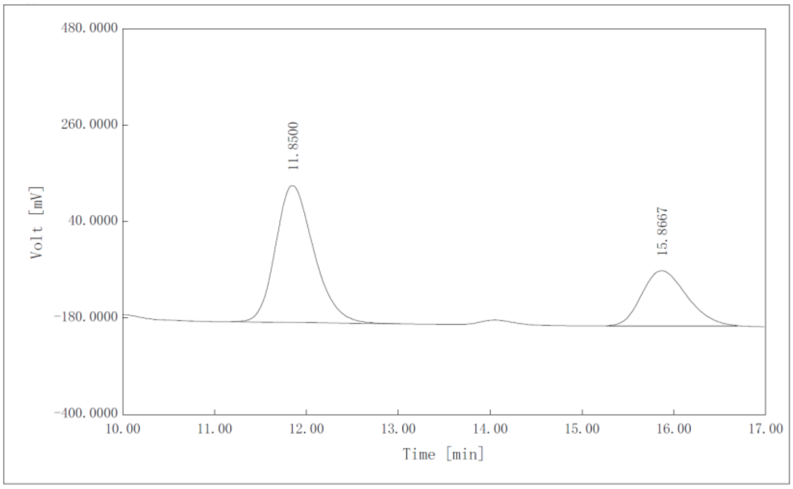

b

Serial Number	Retention Time[min]	Area[mAbs*s]	Type	Area%
1	11.8000	14735.2524	BB	66.0945
2	15.6167	7558.9610	BB	33.9055
The Total		22294.2134		

Supplementary Figure 43. HPLC spectra for the third run. a, After reaction. **b**, The detail of integration.

a

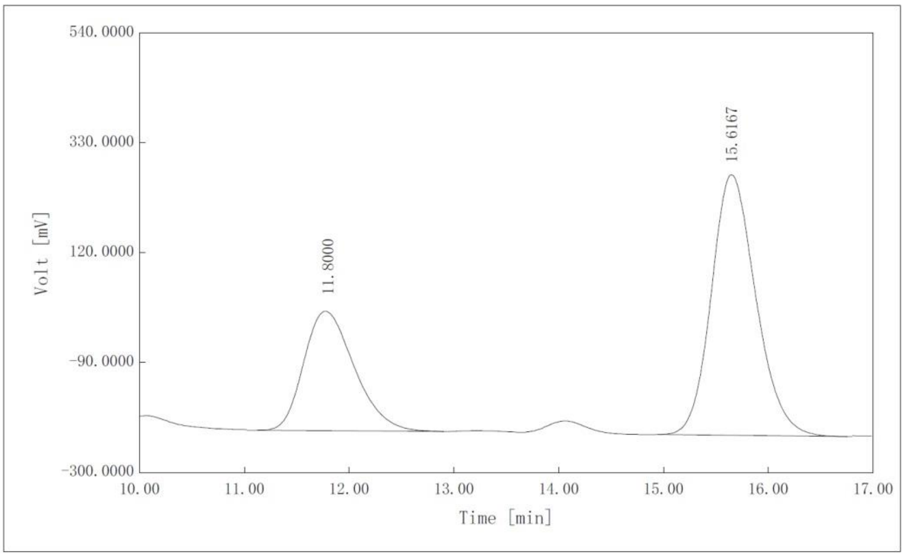

b

Serial Number	Retention Time[min]	Area[mAbs*s]	Type	Area%
1	11.8000	7639.0579	BB	34.1670
2	15.6167	14718.9636	BB	65.8330
The Total		22358.0215		

Supplementary Figure 44. HPLC of 1-(4-bromophenyl)-2-nitroethanol catalysed by (*Λ*)-TpTab-Cu. a, After reaction. **b**, The detail of integration.

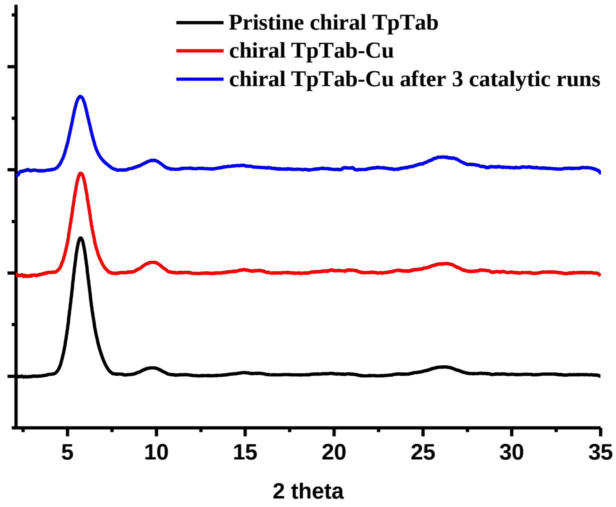

Supplementary Figure 45. PXRD of chiral TpTab-Cu after three catalytic runs.

Supplementary Table 11. Henry reaction catalyzed by (*Δ*)-TpTab-Cu.[a]

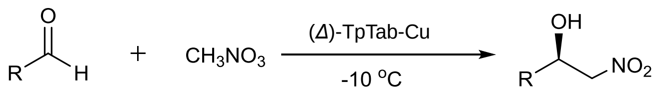

Entry[a]	R	conv (%)	ee (%)
1	*p*-BrC_6H_4	10 (90)[b]	35 (5)[b]
2	*p*-$NO_2C_6H_4$	13 (95)[b]	nd
3	*p*-MeC_6H_4	5 (52)[b]	nd
4	coronenyl	trace (trace)[c]	nd

[a]All reactions were carried out with stirring for 48 h at -10 °C in mesitylene: 10 mol% loading of (*Δ*)-TpTab-Cu, 0.4 eqiv. DIEA, 2.0 eqiv. CH_3NO_2. [b]2.4 eqiv. DIEA was used for the reaction. [c]4.0 eqiv. DIEA was used for the reaction.

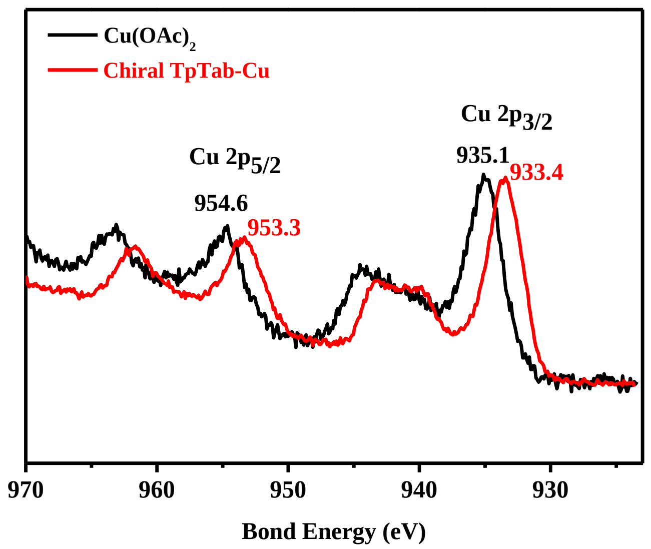

Supplementary Figure 46. The X-ray photoelectron spectra of the $Cu(OAc)_2$ and chiral TpTab-Cu.

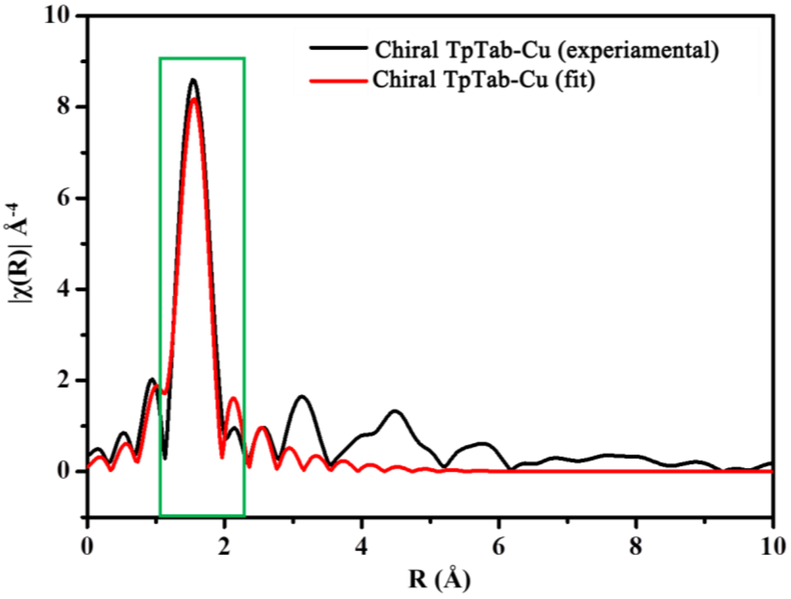

Sample	Cu-O		D. W.	ΔE_0 (eV)
	R (Å)	CN		
Cu-N	1.96±0.02	0.9±0.4	0.003	10.9±1.3
Cu-O	1.97±0.02	3.0±0.4	0.003	11.9±2.8

Supplementary Figure 47. Experimental XAFS spectra in chiral TpTab-Cu (black) and fits (red).

Supplementary References.

1. Chong, J.H., Sauer, M., Patrick, B.O. & MacLachlan, M.J. Highly Stable Keto-Enamine Salicylideneanilines. *Org. Lett.* **5**, 3823-3826 (2003).
